# Secretome analysis of *Trypanosoma cruzi* by proteomics studies

**DOI:** 10.1371/journal.pone.0185504

**Published:** 2017-10-03

**Authors:** Jean-Yves Brossas, Julián Ernesto Nicolás Gulin, Margarita Maria Catalina Bisio, Manuel Chapelle, Carine Marinach-Patrice, Mallaury Bordessoules, George Palazon Ruiz, Jeremy Vion, Luc Paris, Jaime Altcheh, Dominique Mazier

**Affiliations:** 1 Centre d'Immunologie et des Maladies Infectieuses, INSERM U1135, Paris, France; 2 Sorbonne Universités, UPMC Univ Paris 06, Paris, France; 3 Service de Parasitologie-Mycologie, Hôpital Pitié-Salpêtrière, AP-HP, Paris, France; 4 Servicio de Parasitología y Enfermedad de Chagas, Hospital de Niños “Dr. Ricardo Gutiérrez”, Buenos Aires, Argentina; 5 Instituto de Investigaciones en Patologías Pediátricas, CONICET, Buenos Aires, Argentina; 6 Bruker Daltonics, Champs-sur Marne, France; New York University School of Medicine, UNITED STATES

## Abstract

**Background:**

Chagas disease is a debilitating often fatal disease resulting from infection by the protozoan parasite *Trypanosoma cruzi*. Chagas disease is endemic in 21 countries of the Americas, and it is an emerging disease in other countries as a result of migration. Given the chronic nature of the infection where intracellular parasites persist for years, the diagnosis of *T*. *cruzi* by direct detection is difficult, whereas serologic tests though sensitive may yield false-positive results. The development of new rapid test based on the identification of soluble parasitic antigens in serum would be a real innovation in the diagnosis of Chagas disease.

**Methods:**

To identify new soluble biomarkers that may improve diagnostic tests, we investigated the proteins secreted by *T*. *cruz*i using mass spectrometric analyses of conditioned culture media devoid of serum collected during the emergence of trypomastigotes from infected Vero cells. In addition, we compared the secretomes of two *T*. *cruzi* strains from DTU Tc VI (VD and CL Brener).

**Results:**

Analysis of the secretome collected during the emergence of trypomastigotes from Vero cells led to the identification of 591 *T*. *cruzi* proteins. Three hundred sixty three proteins are common to both strains and most belong to different multigenic super families (*i*.*e*. TcS, GP63, MASP, and DGF1). Ultimately we have established a list of 94 secreted proteins, common to both DTU Tc VI strains that do not belong to members of multigene families.

**Conclusions:**

This study provides the first comparative analysis of the secretomes from two distinct *T*. *cruzi* strains of DTU TcVI. This led us to identify a subset of common secreted proteins that could potentially serve as serum markers for *T*. *cruzi* infection. Their potential could now be evaluated, with specific antibodies using sera collected from patients and residents from endemic regions.

## Introduction

*Trypanosoma cruzi* is a protozoan parasite of the order Kinetoplastide and the aetiological agent of Chagas disease a vector-borne infection with a high prevalence in Central and South America. According to recent estimates, nearly 6 million people are infected with this neglected disease in Latin American countries [1]. Moreover, Chagas disease is now an increasing global health problem because of increased migration of infected persons to non-endemic regions [2].

*T*. *cruzi* reproduction is mostly clonal, with occasional events of genetic exchange leading to the emergence of hybrid genotypes [3]. These features led to a complex population structure, showing remarkable genetic diversity [4]. Biochemical and genetic typing schemes developed throughout the last decades converged in the delineation of six major *T*. *cruzi* evolutionary *lineages* or discrete typing units (DTUs) termed TcI to TcVI [5]. However the remarkable genetic heterogeneity of *T*.*cruzi* could partially account for their wide range of biological features, eco-epidemiological traits, and the large spectrum of clinical manifestations of Chagas disease [6]. No clear correlation between serodiagnostic test reactivity and molecular diversity of T. cruzi has been observed. Instead, discrepancies between serologic test’s sensitivity may reflect adaptive immune responses to parasite antigens [7]. In human parasite transmission occurs by vectorial route when metacyclic trypomastigotes present in blood-sucking triatomine bug faeces penetrate the skin or mucous membranes. However, humans can also become infected via blood transfusion or organ transplantation [8], through the ingestion of tainted food and fluids [9], or via vertical transmission from mother-to-child during pregnancy or delivery [10][11].

The clinical course of the *T*. *cruzi* infectionhas three phases: acute, chronic without symptoms, and chronic with symptoms [12]. The initial acute phase, lasting for about 2 months, is characterised by high levels of trypomastigotes (the circulating form of *T*. *cruzi*) in the blood. This is followed by a chronic phase (with or without symptoms) characterized by low trypomastigotes levels but with many intra-cellular parasites (amastigotes) present in target tissues (cardiac and smooth muscle) that can lead to severe morbidity. During this stage diagnosis is essentially based on the detection for classical (not lytic) anti-*T cruzi* antibodies in patient serum. At present, there are no diagnostic criteria for treatment response because anti-*T*. *cruzi* antibodies can persist many years after parasitological cure. Therefore, there is an urgent need to find a means to improve diagnosis in order to evaluate treatment efficacy.

A biomarker is defined as a parameter that can be objectively measured as an indicator of normal or pathogenic biological processes, or as an indicator of pharmacological responses to therapeutic interventions[13] [14]. In infectious diseases, there are two main types of markers: markers from the host and markers from the pathogen[15,16]. Only the latter markers provide evidence for the presence of the pathogen. Such markers can be of different types (carbohydrate, peptide and proteins or chemical product, lipids, sugar or nucleic acid) and from different locations (secreted in the serum of infected patients or present inside infected cells). The detection of circulating parasite excreted-secreted antigens (TESA) in chronically infected persons could serve as suitable biomarkers for the infection and treatment follow-up [17,18]. Proteomic approaches present a unique opportunity for identifying new parasite proteins that are potentially detectable in infected patients. We were particularly interested in extracellular proteins (secreted or released by the parasite), as these are often key mediators of host–parasite interactions involved in immune regulation, signalling, or invasion. Many proteomic analysis of *T*. *cruzi* secretome are already published [19,20]. These analyses made it possible to obtain a better understanding of the diverse pathways of secretion used by different parasite stages (epimastigote or metacyclic forms) to release many proteins into the extracellular medium and many secreted proteins could be identified. However, these studies have been carried out with parasite stages in the absence of vertebrate host cells, and where secretion was artificially induced. We propose here to complement these studies by analysing the secretome of Vero cells infected with two strains of *T*. *cruzi* DTU TcVI [18, 19] CL Brener, the strain used for the first genomic sequence of *T*. *cruzi* [21], with preferential tropism for heart and muscle cells [19] and VD, a strain isolated from a case of congenital Chagas disease, with preferential tropism for plancenta [18].

The main objective was to characterize as comprehensively as possible parasitic proteins secreted by *T*. *cruzi* (TcVI)-infected cells. The ultimate aim of this first step is to establish a list of antigens with potential usefulness as antigenic markers in the diagnosis and treatment follow-up of Chagas disease.

## Materials and methods

### Vero cells culture

Vero cells (normal kidney epithelial cells of *Cercopithecus aethiops*) were obtained from the Virology Laboratory of the Pitié Salpêtrière Hospital (Paris, France). At late exponential growth phase, trypsin-treated Vero cells were subcultured every seven days in RPMI-1640 medium (Life technologies) supplemented with streptomycin/penicillin (Life technologies) and 5% heat-inactivated foetal bovine serum (FBS) (Life technologies). Subcultures were maintained at 37°C in a humidified atmosphere of 5% CO_2_.

### *Trypanosoma cruzi* culture and stocks

CL Brener strain (collection number: MNHN-CEU- 2016–0159) was a gift from Pr. P. Grellier of the Muséum National d’Histoire Naturelle (Paris, France). VD strain (Tc VI) was isolated from a congenital case of Chagas disease diagnosed at the Parasitology and Chagas Disease Service of the “Dr. Ricardo Gutiérrez Children’s’ Hospital” (Buenos Aires, Argentina.) Both strains were maintained in CF-1 (Non “Swiss” albino mice from Charles Rivers laboratory) mice prior to their use.

Large-scale trypomastigote production (CL Brener or VD strains) was achieved as follow. At the end of the exponential phase, Vero cells were harvested from subcultures and seeded into 75 cm^2^ flasks with 10^4^ cells per cm^2^, followed by 3 days of incubation at 37°C in 5% CO_2_ in air. The cells were then infected with *T*. *cruzi* trypomastigotes at a parasite-cell-ratio 1:5. After 24 hours, the flasks were washed with HBSS buffer in order to remove non-attached cells and free parasites, and were subsequently incubated in RPMI-1640 medium. On day 4 (VD strain) or day 5 (CL Brener strain) post-infection, trypomastigotes were released from the cells. The culture medium was removed and transferred to a centrifuge tube. Attached infected cells were washed with 15 mL of HBSS buffer. The culture medium and wash containing trypomastigotes were mixed and centrifuged at 200 g for 10 minutes at room temperature to remove host cells and their debris. Subsequently, trypomastigotes were collected by supernatant centrifugation at 2000 g for 10 minutes, resuspended in 10 mL and counted in a haemocytometer using a light microscope. The recovered parasites served for the secretome analysis.

### Determining the secretome

The parasite suspension was added to each flask containing Vero cells at 7.10^4^ cells per cm^2^, in a 5:1 parasite-cell ratio. After 24 hours, the cells were washed once with HBSS buffer (without Ca^2+^ and Mg^2+^) to remove free parasites from the culture media and they were then incubated for 2 days (VD strain) or 3 days (CL Brener strain) in RPMI-1640 medium-5%SBF. After this, the cells were washed five times with HBSS buffer (with Ca^2+^ and Mg ^2+^) to remove all traces of foetal bovine serum (FBS) and then incubated for 24 hours in a medium used for hybridoma culture (PFHM II),.a synthetic culture medium that contains no proteins. After the release of the trypomastigotes into the culture supernatant, the medium was transferred to a 50 mL centrifuge tube and centrifuged at 2000 g for 10 minutes at room temperature to remove any contamination with host cells, *T*. *cruzi* and other debris. The supernatant was collected and filtered on a 0.22 μm membrane to remove any residual cells or parasites, and then dialyzed and concentrated 200 -fold on a Vivaspin® 15R centrifugal concentrator (Vivaspin) at 4°C. The resultant concentrate was conserved at -80°C until its use.

### One-dimensional electrophoretic analysis

Equal aliquots (40 µg) of proteins obtained from infected Vero cells supernatant were submitted to a sodium dodecyl sulfate-polyacrylamide gel electrophoresis (12% SDS-PAGE). Proteins were denaturized by incubating samples with Laemlli buffer (0.5 M Tris–HCl, pH 6.8, 50% glycerol, 10% SDS, 5% β-mercaptoethanol, and 0.05% bromophenol blue) at room temperature for 16 hours before electrophoresis. Electrophoresis was carried out running buffer (50 mM Tris, 192 mM glycine and 0.1% SDS). After protein separation, gels were stained with Instant Blue (Expedeon Ltd., Harston, U.K.) for 20 minutes and transferred to distilled water for direct use.

### In-gel digestion

Each track was cut into 9 gel bands using a sterile scalpel and these were individually transferred to 1.5 mL Eppendorf sterile tubes. The gel was covered with 200 μL of destaining solution (50% acetonitrile, 25 mM ammonium bicarbonate in Milli-Q-H_2_O) and incubated under stirring at room temperature for 10 minutes. This step was repeated until the blue colour was removed. The gel pieces were then dehydrated using 200 μL of acetonitrile for 5 min at room temperature and air dried for 30 min.

Subsequently, 100 μL of buffer (10mM dithiothreitol (DTT), 100 mM ammonium bicarbonate) were added to reduce proteins for 30 minutes at 56°C. Next, 100 μL of 50 mM iodoacetamide buffer was added at room temperature for 15 min. Excess solution was removed and 200 μL of acetonitrile were added to dehydrate gel pieces for 5 min at room temperature until white coloration appeared. Excess acetonitrile was removed and the gel was further air dried until complete dryness (30 min, in general).

Trypsin reagent was prepared by adding 1 mL of ice-cold 50mM ammonium bicarbonate to 20 μg of trypsin (Promega) (final concentration 20 ng/ μL) and the solution was kept on ice. Then, 30 μL of trypsin solution was added to samples to rehydrate gel pieces for 10 min on ice while gently mixing. A total of 30 μL of 50 mM ammonium bicarbonate was added to gel pieces and trypsin digestion was carried out overnight at 37°C. Excess solution was removed and transferred into a new 1.5 mL tube.

30 μL of extraction buffer (50% acetonitrile, 1% tri-fluoro-acetic acid (TFA) in Milli-Q-H2O), was sequentially added to gel pieces for incubation for 10 minutes and transferred into the same tube. After a last incubation with extraction buffer, the samples were completely dried in a vacuum centrifuge and then resuspended in 30 μL of buffer A (98% Milli-Q-H2O, 2% acetonitrile, 0.1% TFA).

### Mass spectrometry analysis

#### Nano LC–MS/MS

Protein digests were analyzed by a Q-TOF mass spectrometer (Impact II, Bruker Daltonik GmbH; Bremen, Germany) using a Captive Spray source, interfaced with a nano-HPLC RSLC System (Ultimate 3000 Thermo Scientific). Samples were concentrated on a pre-column (Thermo Scientific, C18 PepMap100, 2 cm × 100 μm ID, 5 μm particle size, 100 A) at a flow rate of 10 μL/min using 0.1% tri-fluoro acetic acid. After pre-concentration, peptides were separated on a Thermo Scientific C18 PepMap100 UHPLC column (50 cm x 75 μm ID, 2 μm particles, 100A) at a flow rate of 400 nL/ min using a 90 min linear gradient (buffer A: 2% ACN in 0.1% FA; buffer B 100% ACN in 0.1% FA). Acquisition is data dependent InstantExpertise™ mode (Bruker Daltonik GmbH; Bremen, Germany) for precursor selection based on fixed time (1 MS every 2 sec at 0.25 sec/scan) for MS and as many as possible MSMS in between (1.75 sec) at acquisition time from 0.0625s to 0.25 s/scan based on precursor intensity.

#### Protein identification with reference to databases

MS/MS raw data were processed using the Data Analysis software (Bruker Daltonik GmbH; Bremen, Germany) to generate the peak lists. The resulting mgf (Mascot Generic File Format) were submitted to Mascot server version 2.5 (MatrixScience–London, UK) through Proteinscape platform version 4.0 (Bruker Daltonics). Files were then searched against a home-built database (38685 entries) made of compiled Chlorocebus sabeus protein database from UniProtKb (19441 entries) and *T*. *cruzi* (strain CL Brener) protein databases from UniProtKb (19244 entries) using trypsin as hydrolysis enzyme. Mass tolerances were set at 10 ppm and 0.01 Da for precursor and product ions, respectively. Two missed cleavages per peptide were allowed considering both tryptic and semi-tryptic cleavages, while the search included fixed carbamidomethylation of Cysteine, and the following variable modifications: N-terminal acetylation, deamidation of asparagines and glutamines and, methionine oxidation. Searches were filtered using both a minimum peptide ion score of 20 and a false discovery rate (FDR) <1% calculated using Percolator.

## Results

We opted to analyse all the proteins secreted from Vero cells infected with trypomastigotes of either one of two strains, CL Brener and VD, as a means to derive a list of proteins that could be potential biomarkers for a *T*. *cruzi* infection.

In our *in vitro* infection model, following the multiplication of the intracellular amastigotes and their differentiation into trypomastigotes, the parasites are released from the cells in the culture medium. The culture supernatant is defined in this study as the "secretome", and it includes: 1) soluble proteins from Vero cells, 2) parasite proteins secreted into Vero cells cytoplasm that are then released into the culture media, and 3) all the trypomastigote proteins that have been directly secreted into the culture supernatant.

### Comparing the secretome of two *T*. *cruzi* strains

To increase the validity of the results we selected either proteins whose LC-MSMS score was over 35 and with at least two peptides, or proteins with a score over 50 but with only one peptide identified. In this way, we identified 441 parasites proteins (Additional File 1) from the secretome derived from Vero cells infected with the CL Brener strain, and, a total of 515 proteins (Additional File 2) from Vero cells infected with the VD strain. Since Vero lineage was isolated from kidney epithelial cells extracted from an African green monkey and this study focuses on parasite proteins, those derived from the Vero cells were excluded from further analyses.

**Fig 1:**
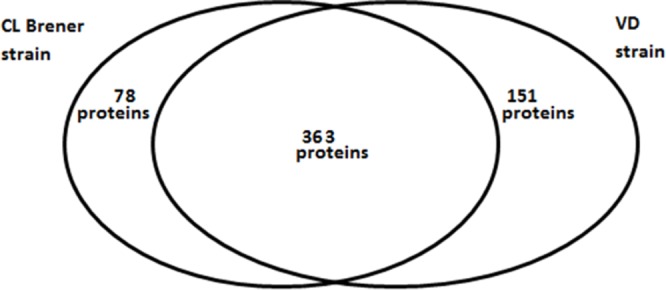
Overlap between secretomes of two different *T*. *cruzi* strains DTU Tc VI. A total of 591 proteins of *T*. *cruzi* were identified. We note that 78 proteins are specific to the CL Brener strain whereas 151 proteins are specific to VD strain. However, 363 proteins are common to both strains.

A total of the 591 *T*. *cruzi* proteins were found in the secretomes of the 2 strains, 363 were common to both strains (additional file 3), while 78 were exclusive to CL Brener strain and 151 were detected only from the VD strain (see Fig 1).

**Fig 2:**
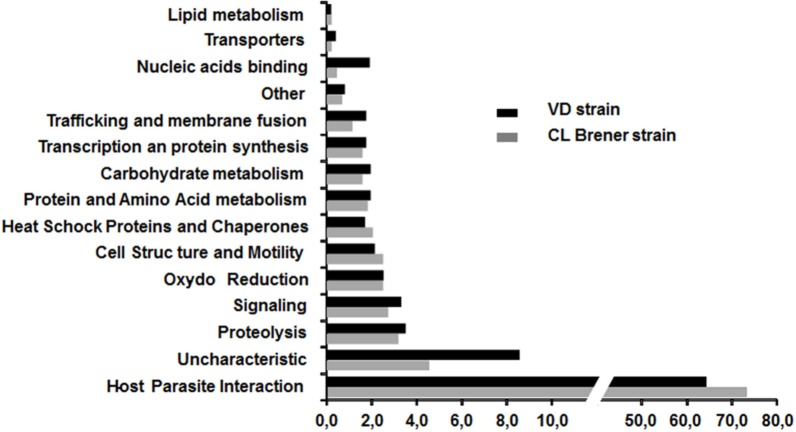
Functional categories classification of *T*. *cruzi* proteins from CL Brener and VD strains. Proteins were classified into 15 functional categories using literature and UniprotKB annotation. Y-axis, categories are indicated. X-axis shows the percentage of each category for each strain.

In order to ascertain the possible role of these secreted proteins, the 363 specific proteins were assigned to 15 functional groups based on information from the literature (Mainly the work of E. Bayer santos *et al*) and UniprotKB annotation (see Fig 2).

For both strains, approximately 70% of the proteins belong to the host-parasite interaction category that plays a very important role in virulence. A significant difference is remarkable; the number of identified proteins in the secretome with unknown function for the VD strain (44 proteins) is slightly more twice that for the CL Brener strain (20 proteins). For the VD strain, this subset represents more than 8% of the total proteins identified. A similar difference was noticed for the proteins involved in nucleic acid binding, but with a much lower number of proteins (10 proteins versus 2 proteins, respectively). For the other categories, there was no significant difference between the two strains. The set of *T*. *cruzi* proteins identified for each of the strains is provided in Additional Files 1 and 2.

### Multigenic superfamilies identified in the secretomes

Among the 591 proteins identified in both *T*. *cruzi* secretomes, 379 (64%) correspond to those encoded by different multigenic superfamilies. Trans-sialidase proteins (TcS) and trans-sialidase like proteins are the major families founded in the secretomes of the two strains (315 proteins for TcS, 18 proteins for DGF1, 15 proteins for Masps and 31 proteins for GP63 surface proteins). A total of 315 TcS proteins from the *T*. *cruzi* secretomes have been identified for the 2 strains. Among these proteins, 46 are fragments of TcS. We can remark that 184 complete TcS proteins were common to both strains. This implies that 42 proteins are exclusively found in CL Brener strain and 43 for VD strain.

**Fig 3:**
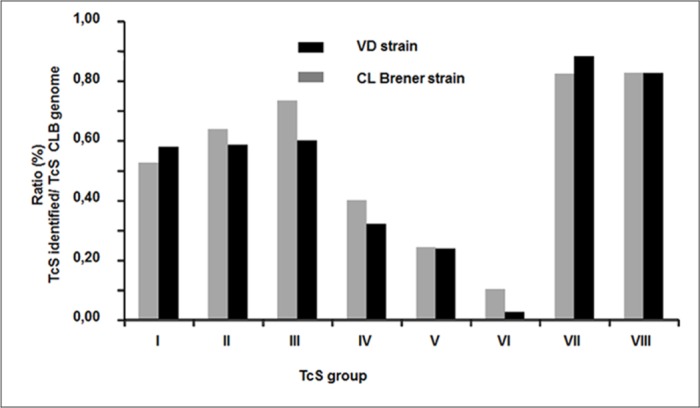
Analyse of trans-sialidase (TcS) proteins found in secretome of 2 strains. Classification of TcS proteins for each strain into 8 group previously described [22]. Groups IV, V and VI are less than I, II, III groups VII and VIII for two strains. On the x-axis, the number of group is indicated. The Y-axis shows the percentage of each TcS identified in our analyses.

Since, previous studies have shown the presence of 508 complete TcS genes that can be classified into 8 Groups [22], we analysed the expression profile of these TcS in the two secretomes and classified these TcS proteins into groups I to VIII, according to previously described classes. For a better description of the expression of different TcS groups, we derived the ratio between the number of TcS identified in our analysis, and that in each group (see Fig 3).

## Discussion

A reliable biomarker for the *T*. *cruzi* infection could be of different nature (protein, lipid, sugar or nucleid acid) but it must be specific to *T*. *cruzi* and ubiquitous in the different *T*. *cruzi* genotypes. The objective of this work was to obtain and compare the secretomes of two *T*. *cruzi* strains infecting Vero celIs, as a way to focus on protein biomarkers for Chagas disease.

For this study we chose to work with two *T*. *cruzi* strains of the same DTU (TcVI). The genetic intra-DTU variability in the parasite was poorly studied. The *T*. *cruzi* CL Brener genome (TcVI) was published in 2005 [21]. Other genomes, such as that of the Sylvio X10/1 strain and DM28c (TcI), have also been published [23,24]. The VD strain was isolated from a case of congenital Chagas disease and its genome is not sequenced. The comparison of two strains of TcVI genome but with different tropism increases the degree of selection of the most important secreted proteins and eliminated the strain-dependent proteins.

### Secretome comparison

We have identified 515 proteins for VD strain compared to 441 proteins for the CL Brener strain. The result of the comparison shows a high proportion (more than 60%) of protein secreted common to both *T*. *cruzi* strains. It would seem that gene expression in both *T*. *cruzi* strain of the same DTU is relatively homogeneous. This high degree of homology makes possible to identify proteins secreted by all strains of *T*. *cruzi* and thus potential markers. This result is very different from that obtained by Geiger *et al*. who analyzed the secretome of *T*. *brucei* and found a small number of proteins common to the two strains of the same group (36 proteins common to the Feo strain and the strain OK out of a total of 270 identified proteins [25].

After functional classification of the parasite proteins, we noted that the proportion of identified proteins involved in proteolysis, signalling, oxydo-reduction, cell structure and motilities was quite different for the two strains. This result suggests that proteins involved in metabolic pathways of stress response, protein folding, proteolysis, and oxidoreductase activity, along with proteins that can interact with different signalling pathways, are important for *T*. *cruzi*’s capacity to infect its host cell, and that these are probably more conserved in all *T*. *cruzi* strains. Furthermore, proteins involved in DNA binding are also more numerous in the secretome of the VD strain as compared to that of the CL Brener strain. This class of proteins plays an essential role in the control of gene expression in *T*. *cruzi*, modulating RNA processing stability, turnover, and translation [26].

The VD strain secretome contained more proteins with unknown functions than the CL Brener strain. The VD strain was isolated from a pediatric patient with congenital infection. As previously noted from *in vivo* investigations [27,28], in our study the VD strain seemed more virulent than the CL Brener strain due the faster emergence from infected Vero cells. The hypothetical proteins of unknown function could be involved in virulence, and also be a source of biomarkers candidates for strain discrimination and for further studies to understand the underlying mechanisms of pathogenicity.

However, more than 70% of the proteins that have been identified in the secretome of the two strains belong to the host-parasite interaction category, and these play a very important role as virulence factors. In this class there are essentially four major multigenic proteins families. A remarkable feature of the *T*. *cruzi* genome is the massive expansion of these multigenes family that encodes polymorphic surface proteins such as trans-sialidase (TcS), mucin and mucin-associated surface proteins (MASPs), dispersed gene family 1 (DGF-1), and gp63 peptidases. The gene families coding for these proteins represent about 10% to 30% of the *T*. *cruzi* genome [29]. The high sequence variability of these different gene families could confer upon the parasite the ability to invade a significant number of cell types, as well as to have pleiotropic tissue tropisms, as described previously in *in vitro*, and in *in vivo* and clinical studies [30] where a wide variety of syndromes associated with *T*. *cruzi* infection were described.

The trans-sialidase and tran-sialidase like gene (TcS) super family represents the largest *T*. *cruzi* gene family, with more than 1,400 genes. However, only half of these genes are apparently functional [21]. The TcS superfamily is composed of glycosylphosphatidylinositol proteins anchored on *T*. *cruzi* surface, but they are also released to the extracellular space. The TcS gene family is highly polymorphic and only a few members have critical residues necessary for catalytic activity [31]. The very large number of different TcS proteins found in both secretomes is not unexpected. It has been shown that TcS expression is highly induced when cells become full of parasites and when amastigotes transform to trypomastigotes [32]. Thus, it would be possible to correlate the presence of large amounts of TcS into culture medium with cell host rupture. Finally, Affranchino JL *et al*. reported that Vero cells infected with trypomastigotes release vesicles carrying some protein members of the trans-sialidase multigene family [33]. However, our study provides a more detailed description (number and identification) of the TcS members expressed during the life cycle of *T*. *cruzi* (DTU TcVI). Recently, a comparative analysis of TcS sequences (508 complete genes) in the *T*. *cruzi* CL Brener strain genome led to the differentiation of eight different groups [22]. These authors further showed that the majority of TcS transcripts of all groups are present both in trypomastigotes and amastigotes forms. However, some transcripts are highly expressed in trypomastigote stage whereas other transcripts are expressed in amastigotes. It is interesting to note that Freitas LM and their colleagues found a very low level of expression for two genes from TcS group V in all the developmental stages [22]. The protein structure of the TcS groups V and VI are very similar. Trans-sialidases of these two groups have one signal peptide, a single asp box, a canonical VTV motif and a GPI anchor [22]. Our study appears to confirm these observations since we observed that more than half of the TcS proteins belonging to groups I, II and III, are expressed (19, 117 and 15 proteins, respectively). This percentage increases up to 80% for the VII and VIII groups (17 and 46 sequences, respectively). While for group IV the expression of TcS is less than 40%, and for groups V and VI less than 25% (227 and 39, respectively). This observation is noteworthy, because groups V and VI represent more than 60% of the trans-sialidases (TcS).

Finally, antibodies against two repeated epitopes, SAPA epitope (PVDSSAHG/STPST) and TcD epitope (PKPAE) of trans-sialidases were found in the sera of patients with Chagas' disease in acute and chronic phase [34]. Our sequence analysis shows trans-sialidases that possess the SAPA epitope (Group I) and the TcD epitope (Group IV) in the secretome of both analysed strains. There is a high homology in the TcS group expressed in both strains from same DTU (TcVI), we could suppose that some variations in their expression might be related to the genetic difference between strains. Numerous proteomic studies have revealed that the *T*. *cruzi* secretomes contain many proteins. The first secretome analysis has been made from ‘reservosome’ fraction belonging to Dm28c strain (TcI) epimastigotes [19], suggesting that there are several possible pathways for the secretion of proteins from *T*. *cruzi*. In another study [25] the authors identified *T*. *brucei* proteins from purified microvesicles extracted from infected rats sera.

In the latest and most comprehensive study [20], the authors identified two types of microvesicles released by epimastigotes and metacyclic forms of Dm28c *T*. *cruzi* strain. All these studies pointed to the use of microvesicles to deliver cargo of many proteins into host cells. The role of the proteins released from microvesicles is fundamental in Chagas disease. For example, Cestari *et al*. observed microvesicles release from infected cells into bloodstream that could allow immune evasion by inhibiting C3 convertase [35]. The protein content of the microvesicles was found to be ubiquitous, contributing to virulence, infectivity and immunity in many pathogens such as *Trypanosoma spp*. and other related parasites such as *Leishmania spp*. [36] and *Plasmodium* spp. [37], but also from bacteria [38] or virus [39].

Nevertheless, all these studies were carried out without the host cell context. In the present study, we analyzed the secretome of *T*. *cruzi* in the presence of host cells and particularly at the time when trypomastigotes are released from the cell.

In our model we have identified a set of parasite proteins that have been released from the host cells by secretion, by microvesicles, or directly from the cell cytoplasm to the culture medium. These proteins are linked to biochemical processes that enable the transformation of amastigotes in trypomastigotes, the multiplication of amastigotes, the release of trypomastigotes from host cells and finally invasion of new cells. In the culture medium, we had access to all the proteins secreted or excreted by the parasite.

Our data show that the majority of the proteins identified for both strains were found in the microvesicles of the epimastigote and the metacyclic forms of *T*.*cruzi*. However, we have also identified some proteins that were previously found only in microvesicles from epimastigotes, such as the 10 KDa heat shock protein [20]. This result suggests that there are slight differences in the secretome of trypomastigote and amastigote forms of *T*. *cruzi*. Although these parasitic proteins are not required for cell invasion (metacyclic form) they may have a role in survival within host cell cytoplasm.

### Short list of parasite proteins as a potential marker of infection with *T*. *cruzi*

Although the protein superfamilies (TcS, GP63, MASP, and DGF1) are highly represented in the secretome of both strains, they cannot be used as biomarkers for *T*. *cruzi* infection. The sequence identity of the TcS is low and is often limited to common motifs (Asp-box, GPI anchor, VTV and FRIP box). These motifs include a maximum of ten amino acids. In addition it should be noted that these proteins have sequences that are fairly variable between the various *T*. *cruzi* strains. Pattern searches by the protein BLAST algorithm from National Center for Biotechnology Information (NCBI)show that the TcD epitope is repeated 430 times in the genome of *T*. *cruzi* strain CL Brener while it is identified only 5 times in strain DM28C. Similarly we did not find the SAPA epitope In the DM28C strain whereas in the strain CL Brener this pattern is repeated 80 times. So we propose a selected list of proteins (see Table 1) that could potentially serve as markers of an infection with *T*. *cruzi*. Indeed, these proteins are secreted, clearly produced abundantly, and are common to at least two strains of TcVI.

**Table 1:** List of secreted proteins that are common in both *T*.*cruzi* strains without of multigene proteins.

Proteins	UNIPROT Accession number		MW (Kda)	
10 kDa heat shock protein, putative	Q4DFA8		10.7	
40S ribosomal protein S15a, putative	Q4E0N6		14.7	
40S ribosomal protein S18, putative	Q4E093		17.5	
Actin, putative	Q4D7A6		38.1	
Adenosylhomocysteinase	Q4D455		48.4	
ADP-ribosylation factor 1, putative	Q4D7Y8		20.7	
Alpha tubulin, putative	Q4CLA1		49.8	
Arginine kinase, putative	Q4CWA5		40.2	
ATP synthase subunit beta	Q4DTX7		55.7	
Beta tubulin, putative	Q4DQP2		49.7	
Calcium-binding protein, putative	Q4D1Q2		19.6	
Calmodulin, putative (Fragment)	Q4D2S5		9.5	
Calpain-like cysteine peptidase, putative	Q4D066		12.8	
Calreticulin, putative	Q4DDX3		46.2	
Chaperonin HSP60, mitochondrial	Q4DYP5		59.1	
Complement regulatory protein, putative	Q4DQ07		113.7	
Cysteine peptidase C	Q4DQB0		36.7	
Dynein light chain, putative	Q4E4N7		10.4	
Elongation factor 1-alpha (Fragment)	Q4CXI2		42.8	
Elongation factor 2, putative	Q4D3T1		94.1	
Enolase, putative	Q4DZ98		46.4	
Glucose-regulated protein 78, putative	Q4D620		71.3	
Glutamate dehydrogenase	Q4D5C2		45	
Glutathione peroxidase	Q4DEJ5		19.7	
Glyceraldehyde 3-phosphate dehydrogenase, putative	Q4D3Y9		14.7	
Glyceraldehyde 3-phosphate dehydrogenase, putative	Q4DHF0		39	
Heat shock 70 kDa protein, mitochondrial, putative	Q4CVR9		70.9	
Heat shock 70 kDa protein, putative (Fragment)	Q4CU95		40.8	
Heat shock 70 kDa protein, putative (Fragment)	Q4DAZ6		30.1	
Heat shock protein 70 (HSP70), putative	Q4DTM9		70.9	
Heat shock protein 85, putative	Q4CQS6		80.7	
IgE-dependent histamine-releasing factor, putative	Q4CW52		19.6	
Isocitrate dehydrogenase [NADP]	Q4E4L7		46.8	
Lysosomal alpha-mannosidase, putative	Q4DXL4		111.2	
Malate dehydrogenase (Fragment)	Q4D4A0		31.5	
Microtubule-associated protein, putative (Fragment)	Q4CMT2		85.2	
NAD/FAD dependent dehydrogenase, putative	Q4CVH0		43	
Neutral sphingomyelinase activation associated protein	Q4DSD4		90.5	
Nucleoside diphosphate kinase	Q4E256		16.9	
Peptidyl-prolyl cis-trans isomerase	Q4E4L9		18.8	
Peptidyl-prolyl cis-trans isomerase	Q4DPB9		21.9	
Phosphoglycerate kinase	Q4D193		44.4	
Proteasome regulatory ATPase subunit 1, putative	Q4D9J1		48.5	
Proteasome regulatory ATPase subunit 2, putative	Q4D0B9		49	
Rab7 GTP binding protein, putative	Q4E4T4		23.9	
Ras-related protein rab-2a, putative (Fragment)	Q4DM40		10.5	
Ras-related protein rab-5, putative (Fragment)	Q4D504		20.4	
S-adenosylmethionine synthase	Q4CSC4		43.5	
Serine carboxypeptidase S28, putative	Q4DM56		72.1	
Serine/threonine-protein phosphatase	Q4D9Y4		35	
Seryl-tRNA synthetase, putative (Fragment)	Q4CW46		25.7	
Small GTP-binding protein Rab1, putative	Q4CZR0		22.8	
Superoxide dismutase	Q4D5A6		21.9	
Transitional endoplasmic reticulum ATPase, putative	Q4DWB5		86.1	
tRNA synthetase, putative (Fragment)	Q4E397		78.8	
Tryparedoxin peroxidase, putative	Q4CM56		22.4	
Tryparedoxin peroxidase, putative	Q4CX87		25.5	
Ubiquitin-activating enzyme E1, putative	Q4DYM1		114.3	
Ubiquitin-conjugating enzyme E2, putative	Q4CTN0		17.5	
Cofilin/actin depolymerizing factor, putative	Q4D8D3	Q4CVE9	15.7	15.7
14-3-3 protein, putative	Q4DJB6	Q4DRH6	29.9	29.9
Cysteine peptidase inhibitor	Q4DH32	Q4DY71	12	12.1
Cysteine peptidase, putative	Q4DW02	Q4E0J7	49,9	49.8
Cytochrome c, putative	Q4D480	Q4CV48	12.2	12.2
Fructose-bisphosphate aldolase	Q4D0Q0	Q4D4R9	40.8	40.8
Serine carboxypeptidase (CBP1), putative	Q4CMQ4	Q4DTP7	59,5	59.5
Uncharacterized protein	Q4D3H5	Q4DNJ6	16.7	16.8
Uncharacterized protein	Q4CNH1		66	
Uncharacterized protein	Q4CPM9		112.1	
Uncharacterized protein	Q4CPX4		23.1	
Uncharacterized protein	Q4CUB2		36.5	
Uncharacterized protein	Q4CUQ4		23	
Uncharacterized protein	Q4CVJ1		16.4	
Uncharacterized protein	Q4CW22		13	
Uncharacterized protein	Q4CW23		13.5	
Uncharacterized protein	Q4D1D9		323	
Uncharacterized protein	Q4D6D8		21.1	
Uncharacterized protein	Q4DPV6		24.4	
Uncharacterized protein	Q4DT54		23.6	
Uncharacterized protein	Q4DUX0		22.3	
Uncharacterized protein (Fragment)	Q4CQX1		41.3	
Uncharacterized protein (Fragment)	Q4CTF0		21.3	
Uncharacterized protein (Fragment)	Q4DHN4		38.1	

The list includes 94 proteins of which 8 are represented by two isoforms and 18 have no known function. These various proteins might also be future therapeutic targets and/or markers of Chagas disease. The protein Q4CVJ1 was not identified in the various secretomes published for *T*.*cruzi*, but it has been found in high-throughput screening and is highly expressed and immunogenic; moreover, 68% of sera from patients with Chagas disease recognize this antigen [40]. Finally, the proteins with unknown function, Q4CTF0 and Q4CUB2, have been described as part of the last multigenic family (about 40 members in Cl Brener) subdivided into 3 subfamilies TcTASV-A, B and C. Q4CTF0 is part of the subgroup family TcTASV-A while Q4CUB2 is part of the TcTASV-C family. This subfamily (8 proteins) was identified on the surface of trypomastigotes of *T*. *cruzi* and in (strain CL Brener, Sylvio and RA). About 30% of human sera infected by *T*. *cruzi* reacted with TcTASV-C [41].

We note that for some proteins in this list, Microtubule-associated protein, Heat shock proteins (HSP70), Complement regulatory protein and Flagellar Calcium-binding protein, specific antibodies can be found in the acute and chronic phase sera of patients with Chagas' disease [38]–[42].

The EMBRARIO laboratory (Brazil) offers a confirmatory test (HBK 740 Imunoblot Linhas) based on a multi-epitope recombinant peptide, including the TC-24 epitope (Flagellar calcium-binding protein) and the MAP repeated epitope (Microtubule-associated protein) [43,44].

Some proteins have already been identified as potential markers in other infections. For example, the fructose biphosphate aldolase enzyme, a key enzyme in the glycolytic pathway, was found in plasma of patients infected with *P*. *falciparum* [45]).

The abundance of the parasite proteins and their relative stability in serum are two criteria to help in the identification of suitable markers for *T*. *cruzi* infection. In our study the strategy adopted to obtain the *T*. *cruzi* secretome may eliminate the most labile proteins since the presence of proteases in the culture supernatant is likely to degrade many of the secreted proteins.

## Conclusion

The development and availability of a simple, inexpensive, easy to handle, sensitive and specific method to effectively demonstrate the presence of *T*. *cruzi* from a blood sample is a major challenge for the diagnosis of Chagas disease. Identification of one or more proteins (biomarkers) in the patient's serum will be clear evidence for the presence of the parasite.

This study provides the first comparative overview of the secretomes of cells infected by the *T*. *cruzi* CL Brenner and the VD strains. Not surprisingly, the majority of excreted proteins belong to the four multigenic gene families (TcS, MASPs, DGF1, and gp63 surface protein), that unfortunately cannot be used as infection marker because of their very high, inter-species, degree of variability.

Some of the proteins identified had not been studied previously, particularly from the VD strain, and some may be new potential therapeutic targets. We have thus established a list of 94 secreted proteins, common to both strains and that do not belong to members of multigene families. These proteins are secreted in large quantities and are relatively stable in presence of many proteases. These conditions are necessary to identify good serum markers. This descriptive study provides the first step toward the identification of potential diagnostic markers in the sera of *T*. *cruzi*-infected persons.

## Supporting information

S1 Table Secreted parasites proteins identified in T. cruzi CL Brener strains separated on 1D gel and their classification according to functional categories (informations from literature and UniprotKB annotation). The proteins secreted have selected with LCMSMS score was over 35 and with at least two peptides identified, or with a score over 50 but with only one peptide identified. For each protein, the number of matched proteins and peptides and the highest score are described.
(PDF)

S2 Table Secreted parasites proteins identified in *T*. *cruzi* VD strains separated on 1D gel and their classification according to functional categories (informations from literature and UniprotKB annotation). The proteins secreted have selected with LCMSMS score was over 35 and with at least two peptides identified, or with a score over 50 but with only one peptide identified. For each protein, the number of matched proteins and peptides and the highest score are described.
(PDF)

S3 Table Secreted parasites proteins identified in *T*. *cruzi* VD and CL Brener strains (common proteins) separated on 1D gel. For each protein, the number of matched proteins and peptides and the highest score for CL Brener (white box) and VD (gray box) strain are described.
(PDF)
