# Supplementary material for: Secretome analysis of Trypanosoma cruzi by proteomics studies
Source: PLoS One. 2017 Oct 3;12(10):e0185504. doi: 10.1371/journal.pone.0185504 (PMC5626432; doi:10.1371/journal.pone.0185504)
Supplement: S1 Table — The proteins secreted have selected with LCMSMS score was over 35 and with at least two peptides identified, or with a score over 50 but with only one peptide identified. For each protein, the number of matched proteins and peptides and the highest score are described. (PDF) [file pone.0185504.s001.pdf]

S1 Table

Protein description	Accession N°		MW [kDa]	pI	Matching Proteins	Matching Peptides	Highest Scores
	ID Gene	Uniprot					
Host-Parasite Interaction							
Calreticulin, putative	Tc00,1047053509011,40	Q4DDX3	46,2	4,8	2	2	38
Complement regulatory protein, putative	Tc00,1047053509217,160	Q4DQ07	113,7	4,9	2	10	573,8
Dispersed gene family protein 1 , putative	Tc00,1047053507083,20	Q4DPN1	354,1	7,3	150	28	942
Dispersed gene family protein 1 (DGF-1), putative	Tc00,1047053506415,10	Q4CZ77	354,4	7,2	1	24	802,5
Dispersed gene family protein 1 , putative	Tc00,1047053509657,20	Q4DH83	360,3	6	1	3	223,6
Dispersed gene family protein 1 (DGF-1), putative	Tc00,1047053510465,10	Q4DVF3	360,8	6	3	2	212,3
Dispersed gene family protein 1 , putative	Tc00,1047053511767,20	Q4DKA8	359,7	5,6	1	2	195,3
Dispersed gene family protein 1 , putative	Tc00,1047053510455,10	Q4CQI0	169,4	7,1	4	3	158
Dispersed gene family protein 1 , putative	Tc00,1047053507521,150	Q4DNC0	361,8	6	1	4	142,7
Dispersed gene family protein 1 , putative	Tc00,1047053507845,10	Q4CNC8	85,7	8	1	1	127,5
Dispersed gene family protein 1 (DGF-1), putative	Tc00,1047053510367,10	Q4D6V4	359,9	6	1	2	90,4
Dispersed gene family protein 1 (DGF-1), putative	Tc00,1047053503861,80	Q4DSH9	362	5,8	1	1	58,7
Mucin TcMUCI, putative	Tc00,1047053504167,20	Q4CSC0	17,7	9,1	1	2	86,1
Mucin TcMUCII, putative	Tc00,1047053508495,70	Q4DSN9	20,5	4,8	1	1	152,8
Mucin TcMUCII, putative	Tc00,1047053506741,110	Q4DN88	38	4,4	1	2	36,4
Mucin TcMUCII, putative	Tc00,1047053508747,50	Q4CVU9	37,9	4,4	1	2	35
Mucin-associated surface protein (MASP), putative	Tc00,1047053506763,260	Q4DZB7	48,6	5,2	6	3	459,4
Mucin-associated surface protein (MASP), putative	Tc00,1047053508539,160	Q4DI88	27,5	4,1	1	1	152,5
Mucin-associated surface protein (MASP), putative	Tc00,1047053510377,350	Q4E3U8	46,1	4,4	6	1	137,6
Mucin-associated surface protein (MASP), putative	Tc00,1047053511173,24	Q4E446	55,6	5,4	3	2	77,1
Mucin-associated surface protein (MASP), putative	Tc00,1047053510477,100	Q4D951	27,4	4,7	3	2	67,3
Mucin-associated surface protein (MASP), putative	Tc00,1047053506877,60	Q4CVH5	27,4	4,4	1	1	56,3
Mucin-associated surface protein (MASP), putative	Tc00,1047053511401,100	Q4DDR2	45	4,5	5	1	55,5
Mucin-associated surface protein (MASP), putative	Tc00,1047053508873,10	Q4E519	37,8	4,4	2	2	46,8
Mucin-associated surface protein (MASP), putative	Tc00,1047053504239,220	Q4E065	49,9	4,9	2	2	41,6
Mucin-associated surface protein , putative	Tc00,1047053509905,180	Q4DR50	48,7	4,6	5	3	200,5
Mucin-associated surface protein , putative	Tc00,1047053506599,100	Q4E1Z5	25,5	4,4	13	2	196,1

Mucin-associated surface protein , putative	Tc00,1047053511487,170	Q4DRZ5	40,9	4,8	1	2	180,5
Mucin-associated surface protein , putative	Tc00,1047053510363,320	Q4DXN7	35,2	4,3	2	2	56,5
Mucin-associated surface protein , putative	Tc00,1047053508221,550	Q4E5V3	44,7	4,8	3	1	54,8
Mucin-associated surface protein , putative	Tc00,1047053510979,50	Q4D2L0	54,2	4,7	2	1	50,7
Mucin-like glycoprotein, putative	Tc00,1047053508741,440	Q4E332	37,9	4,9	2	8	780
Mucin-like glycoprotein, putative	Tc00,1047053506289,94	Q4DVZ2	35,3	4,6	6	6	458,3
Mucin-like glycoprotein, putative	Tc00,1047053507359,20	Q4CRJ0	36,5	4,6	1	2	337,6
Mucin-like glycoprotein, putative	Tc00,1047053509123,10	Q4CU27	36,5	4,5	1	3	239,2
Mucin-like glycoprotein, putative	Tc00,1047053511257,110	Q4DGK1	34	4,6	2	3	226,4
Mucin-like glycoprotein, putative	Tc00,1047053508737,10	Q4DTI3	36,7	4,9	1	3	220,5
Mucin-like glycoprotein, putative	Tc00,1047053503655,30	Q4D8Y3	39,3	4,7	2	2	58,4
Neutral sphingomyelinase activation associated factor-like protein	Tc00,1047053509767,10	Q4DSD4	90,5	5,9	1	9	750,8
Neutral sphingomyelinase activation associated factor-like protein	Tc00,1047053509601,10	Q4DK85	90,2	7,5	1	9	739,9
Peptidyl-prolyl cis-trans isomerase	Tc00,1047053506925,300	Q4E4L9	18,8	8,4	1	7	975,6
Peptidyl-prolyl cis-trans isomerase	Tc00,1047053508577,140	Q4DPB9	21,1	9,1	2	6	569,8
Surface protease GP63, putative	Tc00,1047053511153,40	Q4DHC2	82,4	6,5	9	27	3320,9
Surface protease GP63, putative	Tc00,1047053508071,50	Q4D5G1	82,7	5,2	11	38	3221,5
Surface protease GP63, putative	Tc00,1047053507559,110	Q4DAG2	70,5	6,1	1	25	3090,7
Surface protease GP63, putative	Tc00,1047053510303,20	Q4DZ17	68,3	6,3	2	24	3022,2
Surface protease GP63, putative	Tc00,1047053505567,20	Q4CNY8	82,7	5,2	1	30	2692,5
Surface protease GP63, putative	Tc00,1047053510303,140	Q4DZ07	81,1	6,3	1	24	2923,8
Surface protease GP63, putative	Tc00,1047053509259,40	Q4DM64	82,8	5	1	35	2791,8
Surface protease GP63, putative	Tc00,1047053511117,50	Q4D292	99,4	5,9	2	29	2659,3
Surface protease GP63, putative	Tc00,1047053509037,20	Q4CWX0	81,5	7	1	24	2397,9
Surface protease GP63, putative	Tc00,1047053506779,180	Q4DMD2	80,9	6,5	1	20	2096,1
Surface protease GP63, putative	Tc00,1047053506855,220	Q4E1S2	60,2	6,1	1	21	1904,1
Surface protease GP63, putative	Tc00,1047053503479,60	Q4D6Z1	59,9	6,2	1	22	1783,9
Surface protease GP63, putative	Tc00,1047053510139,20	Q4D273	90,8	5,7	1	13	1423,5
Surface protease GP63, putative	Tc00,1047053510307,10	Q4E0N3	90,4	5,6	3	16	1303,4
Surface protease GP63, putative	Tc00,1047053511257,70	Q4DGK5	85,3	5,3	10	12	978,6
Surface protease GP63, putative	Tc00,1047053506289,140	Q4DVY9	88,4	5,2	3	12	873,7

Surface protease GP63, putative	Tc00,1047053509369,50	Q4DCQ9	48,4	8,9	1	8	843,7
Surface protease GP63, putative	Tc00,1047053511257,100	Q4DGK2	85,3	5,7	1	10	841,9
Surface protease GP63, putative	Tc00,1047053506289,170	Q4DVY6	76,7	5,3	1	11	596,8
Surface protease GP63, putative	Tc00,1047053510503,100	Q4DN56	72,6	5,4	14	4	503,8
Surface protease GP63, putative	Tc00,1047053508365,100	Q4DWF5	83,5	5,7	1	5	494,5
Surface protease GP63, putative	Tc00,1047053507637,170	Q4DMQ1	75,7	5,1	1	4	393,2
Surface protease GP63, putative	Tc00,1047053510477,10	Q4D958	38,6	4,6	1	2	183,2
Surface protease GP63, putative	Tc00,1047053510935,20	Q4CMC3	45,1	4,8	1	1	153,1
Surface protease GP63, putative	Tc00,1047053508163,330	Q4E479	77,3	5,3	1	3	115,8
Surface protease GP63, putative	Tc00,1047053510233,10	Q4CUU3	11,8	4,2	1	3	110,1
Surface protease GP63, putative (Fragment)	Tc00,1047053511703,19	Q4CMY9	32,9	5,9	3	15	1784,7
Surface protease GP63, putative (Fragment)	Tc00,1047053507559,10	Q4DAH2	32,3	5	1	14	1593,8
Surface protease GP63, putative (Fragment)	Tc00,1047053509325,4	Q4CNB7	21,3	7,5	1	9	1116,7
Surface protease GP63, putative (Fragment)	Tc00,1047053509373,39	Q4CP23	24,4	8,9	1	9	1029,4
Surface protease GP63, putative (Fragment)	Tc00,1047053504755,10	Q4CM26	40	4,6	3	11	1012,9
Surface protease GP63, putative (Fragment)	Tc00,1047053506057,101	Q4D148	22,1	8,4	1	12	965,7
Surface protease GP63, putative (Fragment)	Tc00,1047053508057,11	Q4CNJ0	20,9	8,6	1	5	725,8
Trans-sialidase, putative	Tc00,1047053508139,240	Q4E1E4	83,9	6,4	2	47	5304
Trans-sialidase, putative	Tc00,1047053510635,10	Q4D232	91	5,4	2	45	5240,6
Trans-sialidase, putative	Tc00,1047053506427,10	Q4CSV1	106,5	4,8	2	49	5043,9
Trans-sialidase, putative	Tc00,1047053504427,230	Q4DYM3	108,6	4,8	1	52	4973,7
Trans-sialidase, putative	Tc00,1047053508627,40	Q4D1A3	113,8	4,9	3	48	4719,9
Trans-sialidase, putative	Tc00,1047053509629,10	Q4CSG0	101,3	4,8	2	51	4628,2
Trans-sialidase, putative	Tc00,1047053506975,90	Q4DGV8	112,5	4,9	1	42	4503,5
Trans-sialidase, putative	Tc00,1047053509157,170	Q4DTD3	107,3	4,9	2	46	4447,4
Trans-sialidase, putative	Tc00,1047053506537,200	Q4DZD4	120,1	5,2	1	42	4240,4
Trans-sialidase, putative	Tc00,1047053511911,60	Q4DQV8	113,6	4,9	1	37	3942,8
Trans-sialidase, putative	Tc00,1047053509265,90	Q4DGT3	86,2	6,1	3	42	3882
Trans-sialidase, putative	Tc00,1047053509843,20	Q4CYS5	95	5,7	1	34	3805,2
Trans-sialidase, putative	Tc00,1047053511129,40	Q4D8H5	113,3	4,9	1	37	3777,9
Trans-sialidase, putative	Tc00,1047053507427,10	Q4CSI1	88,8	5,3	1	34	3676,4
Trans-sialidase, putative	Tc00,1047053506961,25	Q4DVJ1	107,8	4,7	1	30	3626,7
Trans-sialidase, putative	Tc00,1047053511875,20	Q4DA40	99	5,1	1	39	3491,6

Trans-sialidase, putative	Tc00,1047053509495,30	Q4CZ79	99,4	6	7	39	3286,1
Trans-sialidase, putative	Tc00,1047053506331,130	Q4DV16	80,9	6,4	1	29	3262,8
Trans-sialidase, putative	Tc00,1047053503907,10	Q4CRT3	99,6	4,9	2	37	3234,8
Trans-sialidase, putative	Tc00,1047053506217,40	Q4CVZ4	113,6	5	3	28	3181,3
Trans-sialidase, putative	Tc00,1047053508717,60	Q4D9H3	86,1	5,8	1	35	3110,9
Trans-sialidase, putative	Tc00,1047053510787,10	Q4CVS5	124,8	5,8	4	38	3060,9
Trans-sialidase, putative	Tc00,1047053511551,10	Q4CYA8	85,9	5,2	4	26	3013,4
Trans-sialidase, putative	Tc00,1047053510713,30	Q4DUP3	90,3	5,5	3	33	2946,4
Trans-sialidase, putative	Tc00,1047053507085,30	Q4DQ76	94,9	6,3	1	37	2918,6
Trans-sialidase, putative	Tc00,1047053506683,110	Q4DYD0	113,7	4,8	2	36	2891,5
Trans-sialidase, putative	Tc00,1047053506841,20	Q4CTX4	85,9	5,6	1	35	2877,9
Trans-sialidase, putative	Tc00,1047053509513,10	Q4CSG5	81,6	8,7	1	34	2870,4
Trans-sialidase, putative	Tc00,1047053510307,240	Q4E0M8	113,5	4,9	1	31	2847
Trans-sialidase, putative	Tc00,1047053506471,120	Q4DKM3	101,7	5	8	31	2823,7
Trans-sialidase, putative	Tc00,1047053398477,10	Q4CPW8	85,6	8,2	2	32	2748,6
Trans-sialidase, putative	Tc00,1047053506961,150	Q4DVI7	114,1	4,7	1	32	2687,5
Trans-sialidase, putative	Tc00,1047053510483,360	Q4E1F4	92,1	5,4	2	30	2641,8
Trans-sialidase, putative	Tc00,1047053507759,10	Q4CRZ9	113	4,8	2	25	2596,3
Trans-sialidase, putative	Tc00,1047053506757,60	Q4DQA2	87,2	5,8	9	30	2537,1
Trans-sialidase, putative	Tc00,1047053506885,210	Q4E4H9	106,2	5	2	30	2497,9
Trans-sialidase, putative	Tc00,1047053511587,90	Q4DFH1	112,9	4,8	2	30	2417,2
Trans-sialidase, putative	Tc00,1047053506717,80	Q4DX60	113,7	5,1	1	34	2411,4
Trans-sialidase, putative	Tc00,1047053506757,120	Q4DQA0	87,5	5,6	25	27	2410,5
Trans-sialidase, putative	Tc00,1047053507875,70	Q4DWV4	94,4	5,3	1	25	2391,4
Trans-sialidase, putative	Tc00,1047053509529,40	Q4DDZ1	94,2	5,3	1	25	2374,1
Trans-sialidase, putative	Tc00,1047053510643,40	Q4DWU9	113	4,9	1	26	2370,5
Trans-sialidase, putative	Tc00,1047053507479,20	Q4DCG7	89	5,5	2	28	2368,6
Trans-sialidase, putative	Tc00,1047053506723,20	Q4CZC3	93,3	5,8	1	25	2361,6
Trans-sialidase, putative	Tc00,1047053507255,20	Q4D2I1	85,3	6,2	1	28	2332,3
Trans-sialidase, putative	Tc00,1047053506813,140	Q4DY88	89,5	5,5	1	23	2328,4
Trans-sialidase, putative	Tc00,1047053509905,200	Q4DR49	82	5,5	2	20	2314,7
Trans-sialidase, putative	Tc00,1047053506537,80	Q4DZD8	85,6	5,5	2	27	2313,8
Trans-sialidase, putative	Tc00,1047053505993,30	Q4D0D9	111,9	4,8	4	26	2279,4

Trans-sialidase, putative	Tc00,1047053510847,10	Q4DF05	108,5	4,7	1	31	2222,5
Trans-sialidase, putative	Tc00,1047053510907,10	Q4CPF9	103,3	5	3	22	2206,2
Trans-sialidase, putative	Tc00,1047053507121,20	Q4D110	89,1	5,4	2	35	2201,1
Trans-sialidase, putative	Tc00,1047053511779,60	Q4CXY5	101,1	4,9	1	28	2198
Trans-sialidase, putative	Tc00,1047053509549,10	Q4DLR5	85,4	6,6	6	22	2191,1
Trans-sialidase, putative	Tc00,1047053510331,10	Q4CUZ0	106,2	5	1	29	2152,1
Trans-sialidase, putative	Tc00,1047053510125,20	Q4D0T2	113,6	4,8	1	23	2143,9
Trans-sialidase, putative	Tc00,1047053504425,10	Q4DFP2	108	4,8	2	26	2102
Trans-sialidase, putative	Tc00,1047053511833,10	Q4CRY9	112,5	4,8	1	20	2099,4
Trans-sialidase, putative	Tc00,1047053463279,20	Q4CPX0	80,7	6,3	2	25	2070,1
Trans-sialidase, putative	Tc00,1047053510569,10	Q4CPY4	49,7	5,5	1	18	2035,5
Trans-sialidase, putative	Tc00,1047053507839,40	Q4CXS5	80,8	8,4	1	32	2000,6
Trans-sialidase, putative	Tc00,1047053509581,10	Q4D6R7	124,2	5	1	22	1974,4
Trans-sialidase, putative	Tc00,1047053510411,10	Q4D2I7	91,9	5,2	3	22	1957,2
Trans-sialidase, putative	Tc00,1047053509377,20	Q4CUE8	85,7	5,2	1	20	1920,1
Trans-sialidase, putative	Tc00,1047053505365,60	Q4DEB7	113,3	5	1	34	1905,6
Trans-sialidase, putative	Tc00,1047053510163,60	Q4CXW0	83,3	8,8	2	19	1902,7
Trans-sialidase, putative	Tc00,1047053508581,40	Q4CZ95	75,4	6,8	2	21	1902,2
Trans-sialidase, putative	Tc00,1047053506345,90	Q4DCE1	91,6	5	4	24	1890,7
Trans-sialidase, putative	Tc00,1047053508355,80	Q4E3C3	101,8	4,9	1	22	1864,4
Trans-sialidase, putative	Tc00,1047053507611,170	Q4E3G9	85,4	6,1	1	19	1848,7
Trans-sialidase, putative	Tc00,1047053507357,100	Q4DCY7	85,7	8,6	3	21	1828
Trans-sialidase, putative	Tc00,1047053509931,20	Q4CTU4	113,5	4,8	2	17	1827,2
Trans-sialidase, putative	Tc00,1047053507233,10	Q4DEP9	112,3	5	1	22	1767,8
Trans-sialidase, putative	Tc00,1047053506577,80	Q4DR84	99,1	5	1	18	1701,6
Trans-sialidase, putative	Tc00,1047053508521,20	Q4DE13	112,4	4,9	2	22	1693,1
Trans-sialidase, putative	Tc00,1047053511835,10	Q4CPQ2	110,5	5	2	25	1684,8
Trans-sialidase, putative	Tc00,1047053507753,10	Q4CZ80	113,8	5,1	1	19	1668,1
Trans-sialidase, putative	Tc00,1047053506341,50	Q4CYW2	112,2	4,8	2	21	1620,3
Trans-sialidase, putative	Tc00,1047053507313,20	Q4CW78	112,8	5	1	22	1618,6
Trans-sialidase, putative	Tc00,1047053509905,170	Q4DR51	113,1	5	1	19	1615
Trans-sialidase, putative	Tc00,1047053507047,40	Q4DPT3	82	8,6	8	21	1607,4
Trans-sialidase, putative	Tc00,1047053503993,10	Q4CQZ7	76,6	8,7	2	17	1546

Trans-sialidase, putative	Tc00,1047053511669,10	Q4CRD4	113	4,8	2	18	1532,1
Trans-sialidase, putative	Tc00,1047053507473,30	Q4CWN6	81,3	5,2	2	19	1531,8
Trans-sialidase, putative	Tc00,1047053508521,100	Q4DE10	119,9	4,9	1	19	1530,8
Trans-sialidase, putative	Tc00,1047053503759,10	Q4CUS9	81,7	8,9	1	20	1493,6
Trans-sialidase, putative	Tc00,1047053509765,50	Q4DBF1	112,9	4,9	1	23	1490,5
Trans-sialidase, putative	Tc00,1047053504183,20	Q4CR00	88,3	5,4	3	15	1487,2
Trans-sialidase, putative	Tc00,1047053509411,10	Q4CRJ5	89,4	5,4	3	17	1461,4
Trans-sialidase, putative	Tc00,1047053472357,10	Q4CQY9	88	7,5	10	15	1448
Trans-sialidase, putative	Tc00,1047053505207,10	Q4D308	88,6	5,6	1	17	1444,1
Trans-sialidase, putative	Tc00,1047053508045,120	Q4DIN5	108,8	4,7	1	17	1419,7
Trans-sialidase, putative	Tc00,1047053508451,60	Q4D4B5	85,8	5,3	3	15	1416,3
Trans-sialidase, putative	Tc00,1047053507907,20	Q4D625	79	6,2	1	17	1375,5
Trans-sialidase, putative	Tc00,1047053509387,10	Q4CUT1	112,3	5,1	1	21	1361,9
Trans-sialidase, putative	Tc00,1047053510021,120	Q4DUY7	86,9	5,7	1	20	1360,3
Trans-sialidase, putative	Tc00,1047053508061,20	Q4DKB6	89,8	5,4	1	14	1338,7
Trans-sialidase, putative	Tc00,1047053506455,30	Q4CUA7	81,9	6,9	4	18	1321
Trans-sialidase, putative	Tc00,1047053509663,50	Q4DE15	112,4	4,7	1	18	1297,5
Trans-sialidase, putative	Tc00,1047053511885,20	Q4D6K2	84,6	5,6	2	15	1253
Trans-sialidase, putative	Tc00,1047053507949,210	Q4DS03	81,9	8,2	1	16	1237,4
Trans-sialidase, putative	Tc00,1047053511861,50	Q4DMZ9	114,2	4,7	1	13	1223,5
Trans-sialidase, putative	Tc00,1047053510377,330	Q4E3V0	86,9	5,4	8	18	1207,3
Trans-sialidase, putative	Tc00,1047053510729,280	Q4DWW2	105,8	4,8	2	14	1182,1
Trans-sialidase, putative	Tc00,1047053506975,80	Q4DGV9	78	5,1	1	16	1179,5
Trans-sialidase, putative	Tc00,1047053504479,10	Q4CSZ4	93,4	5,3	6	15	1173,5
Trans-sialidase, putative	Tc00,1047053506603,40	Q4D349	82,4	8,5	8	17	1169,8
Trans-sialidase, putative	Tc00,1047053511173,130	Q4E440	98,9	5,1	32	14	1148,8
Trans-sialidase, putative	Tc00,1047053509333,10	Q4CPQ3	108,9	4,8	1	18	1144
Trans-sialidase, putative	Tc00,1047053503861,40	Q4DSI1	112,8	4,8	1	18	1141,8
Trans-sialidase, putative	Tc00,1047053509875,80	Q4E0D0	81,7	8,9	3	16	1135,5
Trans-sialidase, putative	Tc00,1047053509265,120	Q4DGT1	80,5	5,5	7	11	1131,9
Trans-sialidase, putative	Tc00,1047053504491,20	Q4CQ65	56,7	4,8	1	11	1128,9
Trans-sialidase, putative	Tc00,1047053510093,20	Q4CQ85	113,7	4,9	1	15	1128
Trans-sialidase, putative	Tc00,1047053510377,10	Q4E3W2	83,3	8,6	4	15	1123,5

Trans-sialidase, putative	Tc00,1047053509075,50	Q4D8V4	80,2	5,6	1	16	1104,8
Trans-sialidase, putative	Tc00,1047053506763,110	Q4DZC4	114,9	4,8	4	21	1101,5
Trans-sialidase, putative	Tc00,1047053506021,20	Q4CTT0	85,7	5,9	2	14	1097,9
Trans-sialidase, putative	Tc00,1047053508285,60	Q4D825	85,1	5,4	4	24	1094,3
Trans-sialidase, putative	Tc00,1047053507125,10	Q4CTA6	85,8	8,7	1	20	1073,1
Trans-sialidase, putative	Tc00,1047053510979,40	Q4D2L1	91,8	5,9	2	23	1065,1
Trans-sialidase, putative	Tc00,1047053506331,90	Q4DV17	83,2	5,5	1	12	1064,5
Trans-sialidase, putative	Tc00,1047053506597,40	Q4D371	82,8	8,5	5	14	1044,6
Trans-sialidase, putative	Tc00,1047053511173,470	Q4E424	98,7	5	57	11	1037,5
Trans-sialidase, putative	Tc00,1047053506171,60	Q4D5B9	74	5,4	1	9	991,4
Trans-sialidase, putative	Tc00,1047053503601,10	Q4CSS7	85,3	8,7	21	15	988,3
Trans-sialidase, putative	Tc00,1047053507979,30	Q4CZE6	89,4	8,4	3	16	985,1
Trans-sialidase, putative	Tc00,1047053510033,30	Q4CTQ6	82,1	8,4	3	10	967,2
Trans-sialidase, putative	Tc00,1047053506499,170	Q4DU07	82,9	8,8	2	15	963,8
Trans-sialidase, putative	Tc00,1047053505919,20	Q4D8B3	78,6	8,8	1	9	951,9
Trans-sialidase, putative	Tc00,1047053511117,30	Q4D293	81,8	6,3	2	12	948,8
Trans-sialidase, putative	Tc00,1047053505997,80	Q4DWA9	111,4	4,9	1	15	933,7
Trans-sialidase, putative	Tc00,1047053508607,50	Q4DKL5	93,2	6,7	2	13	930,9
Trans-sialidase, putative	Tc00,1047053507819,30	Q4D6J3	85,1	5,6	1	19	928,9
Trans-sialidase, putative	Tc00,1047053504341,10	Q4CSD6	99,9	5,8	10	8	925,6
Trans-sialidase, putative	Tc00,1047053506397,10	Q4CQP5	81	5,8	1	17	910,3
Trans-sialidase, putative	Tc00,1047053508103,30	Q4CQL8	92,3	6,7	4	14	905,3
Trans-sialidase, putative	Tc00,1047053507479,70	Q4DCG5	101,5	5,9	1	9	865,5
Trans-sialidase, putative	Tc00,1047053509281,20	Q4CSS2	79,2	5,9	3	16	864,7
Trans-sialidase, putative	Tc00,1047053506471,100	Q4DKM4	89,6	5,1	1	13	861,9
Trans-sialidase, putative	Tc00,1047053508055,30	Q4D1Z3	85,2	5,9	9	7	855,2
Trans-sialidase, putative	Tc00,1047053507879,10	Q4CPT6	86,4	5,8	2	12	842,1
Trans-sialidase, putative	Tc00,1047053510005,20	Q4DC15	74,6	5,1	1	9	837,4
Trans-sialidase, putative	Tc00,1047053509533,10	Q4CXJ1	80,8	5,1	1	10	821
Trans-sialidase, putative	Tc00,1047053507997,30	Q4D5S3	89,3	5,2	2	9	809,9
Trans-sialidase, putative	Tc00,1047053505609,40	Q4D2F9	83,3	7,2	8	11	797,4
Trans-sialidase, putative	Tc00,1047053508871,10	Q4DQS8	83,5	8,1	29	10	792,2
Trans-sialidase, putative	Tc00,1047053509187,10	Q4CUU4	81,8	9,2	1	13	783,5

Trans-sialidase, putative	Tc00,1047053506609,30	Q4CZA0	86,6	6,2	1	10	777
Trans-sialidase, putative	Tc00,1047053508221,790	Q4E5U1	84,5	8,5	2	9	752,6
Trans-sialidase, putative	Tc00,1047053505779,20	Q4CSU2	80	6,2	3	10	730,1
Trans-sialidase, putative	Tc00,1047053511603,450	Q4E2A1	83,2	8,1	2	6	721,8
Trans-sialidase, putative	Tc00,1047053506459,230	Q4E199	90,2	8,8	1	10	709,6
Trans-sialidase, putative	Tc00,1047053509979,320	Q4E2C9	82,8	8,5	2	11	707,3
Trans-sialidase, putative	Tc00,1047053422867,10	Q4CR14	76,4	5,2	1	8	701,3
Trans-sialidase, putative	Tc00,1047053509785,50	Q4DEK8	87,5	6	7	10	696,9
Trans-sialidase, putative	Tc00,1047053504239,434	Q4E054	40,2	5,7	2	9	694,1
Trans-sialidase, putative	Tc00,1047053507821,130	Q4DH24	88,7	5,4	3	11	686,6
Trans-sialidase, putative	Tc00,1047053508325,230	Q4E0H9	161,1	8,2	3	13	669,8
Trans-sialidase, putative	Tc00,1047053507653,20	Q4DI36	85,8	5,5	1	13	668,9
Trans-sialidase, putative	Tc00,1047053509265,110	Q4DGT2	185,2	6,4	4	6	653,5
Trans-sialidase, putative	Tc00,1047053509097,20	Q4D2W7	83,5	8,7	2	8	644
Trans-sialidase, putative	Tc00,1047053506973,10	Q4DTW9	97,7	4,8	5	8	635,3
Trans-sialidase, putative	Tc00,1047053507875,220	Q4DWV1	86,1	5,3	1	15	632
Trans-sialidase, putative	Tc00,1047053511219,40	Q4D5K5	86,7	5,7	1	17	630,1
Trans-sialidase, putative	Tc00,1047053508581,10	Q4CZ96	90,7	5,7	1	10	614
Trans-sialidase, putative	Tc00,1047053509427,10	Q4D6S1	79,4	5,9	2	9	601,7
Trans-sialidase, putative	Tc00,1047053510403,30	Q4D980	82,2	6,1	1	11	589,4
Trans-sialidase, putative	Tc00,1047053510025,50	Q4DTC3	93,5	8,2	4	8	555,9
Trans-sialidase, putative	Tc00,1047053506683,240	Q4DYC7	80,9	6,2	1	8	540,4
Trans-sialidase, putative	Tc00,1047053511757,70	Q4DBD3	78,9	6,2	1	12	529,2
Trans-sialidase, putative	Tc00,1047053504099,50	Q4DBC9	78,1	5,5	1	7	526,1
Trans-sialidase, putative	Tc00,1047053505975,20	Q4D3K3	152,1	5,6	9	12	520,6
Trans-sialidase, putative	Tc00,1047053506751,50	Q4DDU4	80,3	5,3	1	5	520,4
Trans-sialidase, putative	Tc00,1047053511603,90	Q4E2C0	97,2	5,4	1	11	517,9
Trans-sialidase, putative	Tc00,1047053503447,20	Q4D7N9	82,7	8,9	1	10	515,6
Trans-sialidase, putative	Tc00,1047053506129,50	Q4DFT5	159,2	6,6	1	8	498,8
Trans-sialidase, putative	Tc00,1047053510279,320	Q4E373	98,5	5,4	6	7	488,9
Trans-sialidase, putative	Tc00,1047053506353,10	Q4CQ38	82,7	6,5	2	9	486,7
Trans-sialidase, putative	Tc00,1047053510095,20	Q4DGR6	83,4	6,5	1	6	476,7
Trans-sialidase, putative	Tc00,1047053510465,100	Q4DVF0	95,8	5,2	1	8	475,3

Trans-sialidase, putative	Tc00,1047053509531,10	Q4CLV7	28,2	6	1	4	471,1
Trans-sialidase, putative	Tc00,1047053511911,10	Q4DQW0	80,3	6,3	1	10	467,5
Trans-sialidase, putative	Tc00,1047053511221,60	Q4D781	97,4	4,9	4	5	464,9
Trans-sialidase, putative	Tc00,1047053506595,40	Q4DLC5	72,6	6,6	2	10	461,9
Trans-sialidase, putative	Tc00,1047053506965,170	Q4DIE5	81,2	5,4	1	11	454,3
Trans-sialidase, putative	Tc00,1047053509185,30	Q4CRZ2	85,9	6,9	2	13	450,3
Trans-sialidase, putative	Tc00,1047053506409,170	Q4DV25	98,7	4,9	1	7	449,3
Trans-sialidase, putative	Tc00,1047053505231,20	Q4CRR3	84,2	6,6	2	9	445,8
Trans-sialidase, putative	Tc00,1047053508883,48	Q4CUI1	62,8	8,5	3	6	429,6
Trans-sialidase, putative	Tc00,1047053509685,40	Q4D095	93,7	5,3	5	6	428,3
Trans-sialidase, putative	Tc00,1047053506241,30	Q4DU42	82,3	6,2	3	9	423,8
Trans-sialidase, putative	Tc00,1047053511183,430	Q4E5K1	91,9	4,8	2	5	421,1
Trans-sialidase, putative	Tc00,1047053511173,280	Q4E433	96,8	5,1	3	4	418,4
Trans-sialidase, putative	Tc00,1047053509419,90	Q4DUE4	85,3	6,4	6	5	395,7
Trans-sialidase, putative	Tc00,1047053511057,40	Q4CZ90	84	5,3	4	9	390,5
Trans-sialidase, putative	Tc00,1047053504101,50	Q4CX14	82,5	8,7	1	4	387,3
Trans-sialidase, putative	Tc00,1047053510021,220	Q4DUY0	91,4	5,3	1	8	378,9
Trans-sialidase, putative	Tc00,1047053511185,80	Q4DPD2	91,7	4,9	1	5	369,3
Trans-sialidase, putative	Tc00,1047053508523,30	Q4DL67	87,4	5,5	1	8	365,9
Trans-sialidase, putative	Tc00,1047053509755,10	Q4D649	82,5	5,7	3	8	364,7
Trans-sialidase, putative	Tc00,1047053507071,230	Q4E3D7	96,8	5,5	1	4	364
Trans-sialidase, putative	Tc00,1047053510205,40	Q4D3Q8	83,9	5,5	1	6	343,6
Trans-sialidase, putative	Tc00,1047053510197,30	Q4DWZ2	80,3	4,9	3	3	340,7
Trans-sialidase, putative	Tc00,1047053511401,30	Q4DDR4	97,3	5,3	2	3	339,7
Trans-sialidase, putative	Tc00,1047053507237,10	Q4DYY9	81,5	6,1	2	10	321,1
Trans-sialidase, putative	Tc00,1047053510713,90	Q4DUP2	93,8	5	1	5	311,5
Trans-sialidase, putative	Tc00,1047053508455,20	Q4CWC5	95,1	5,1	4	5	304,3
Trans-sialidase, putative	Tc00,1047053511173,440	Q4E426	82,5	5,4	1	10	291,8
Trans-sialidase, putative	Tc00,1047053511827,110	Q4DQA9	78,4	6,1	1	5	281,6
Trans-sialidase, putative	Tc00,1047053508977,40	Q4DD60	96,8	5,1	12	4	255,2
Trans-sialidase, putative	Tc00,1047053504193,15	Q4CWT8	29,6	9,6	1	3	240,8
Trans-sialidase, putative	Tc00,1047053510491,60	Q4D1A6	93,9	5,5	1	4	234,2
Trans-sialidase, putative	Tc00,1047053508753,20	Q4CXC1	89,9	5,4	3	3	230,7

Trans-sialidase, putative	Tc00,1047053507411,10	Q4CPU0	97,5	5,1	3	2	220,5
Trans-sialidase, putative	Tc00,1047053511873,10	Q4D070	92	5,4	1	8	216,4
Trans-sialidase, putative	Tc00,1047053505699,10	Q4CQV3	109,9	8,1	2	6	198,1
Trans-sialidase, putative	Tc00,1047053511597,10	Q4CSW3	35,3	5	2	2	196
Trans-sialidase, putative	Tc00,1047053506053,50	Q4D6Y6	98,9	6,1	1	4	176,9
Trans-sialidase, putative	Tc00,1047053506951,80	Q4D6F8	93,9	5,1	1	5	160,9
Trans-sialidase, putative	Tc00,1047053504145,10	Q4CP42	81	6,3	1	3	149,6
Trans-sialidase, putative	Tc00,1047053470827,20	Q4CQU8	46,2	8,7	3	1	122,2
Trans-sialidase, putative	Tc00,1047053511061,40	Q4CWQ4	90,5	5,4	1	3	110,4
Trans-sialidase, putative	Tc00,1047053509575,4	Q4CWK0	25,4	9,6	1	4	109,9
Trans-sialidase, putative	Tc00,1047053507163,70	Q4D5F2	97,5	5	1	3	93,2
Trans-sialidase, putative	Tc00,1047053510535,109	Q4DE82	20,1	8,7	1	3	79,4
Trans-sialidase, putative	Tc00,1047053507561,10	Q4CR58	11,4	4,5	1	1	54,8
Trans-sialidase, putative (Fragment)	Tc00,1047053508903,110	Q4DAA5	111,5	4,8	1	45	4843,2
Trans-sialidase, putative (Fragment)	Tc00,1047053507505,10	Q4CR17	74,8	4,8	1	41	3688,3
Trans-sialidase, putative (Fragment)	Tc00,1047053432997,10	Q4CLF9	67,2	5,9	1	34	3033,9
Trans-sialidase, putative (Fragment)	Tc00,1047053510981,20	Q4CQ20	81,4	4,6	2	25	2257,1
Trans-sialidase, putative (Fragment)	Tc00,1047053511105,60	Q4CX91	85,7	4,6	1	25	2143,5
Trans-sialidase, putative (Fragment)	Tc00,1047053508639,10	Q4CL30	28,9	4,5	1	30	2032,7
Trans-sialidase, putative (Fragment)	Tc00,1047053510881,9	Q4CN34	78,7	6,1	2	23	1998,9
Trans-sialidase, putative (Fragment)	Tc00,1047053421173,4	Q4CP99	89,5	5,8	1	23	1906,6
Trans-sialidase, putative (Fragment)	Tc00,1047053506895,80	Q4D266	36,4	9	4	20	1881,8
Trans-sialidase, putative (Fragment)	Tc00,1047053511831,10	Q4CPA0	110,1	4,9	1	21	1809,3
Trans-sialidase, putative (Fragment)	Tc00,1047053510411,40	Q4D2I6	62,3	4,9	1	23	1764,8
Trans-sialidase, putative (Fragment)	Tc00,1047053506253,39	Q4CSN1	60,7	5,9	1	23	1675,3
Trans-sialidase, putative (Fragment)	Tc00,1047053508061,154	Q4DKB4	66,6	8,6	3	23	1631,6
Trans-sialidase, putative (Fragment)	Tc00,1047053433733,10	Q4CKI9	37,8	5,5	1	13	1515,3
Trans-sialidase, putative (Fragment)	Tc00,1047053511757,100	Q4DBD2	62,3	8,9	2	17	1424,5
Trans-sialidase, putative (Fragment)	Tc00,1047053504593,10	Q4CPN3	80,5	4,9	1	18	1388,1
Trans-sialidase, putative (Fragment)	Tc00,1047053510233,39	Q4CUU0	75,5	8	2	17	1368,8
Trans-sialidase, putative (Fragment)	Tc00,1047053508297,7	Q4D3D0	49,5	5,5	1	16	1344,8
Trans-sialidase, putative (Fragment)	Tc00,1047053508165,430	Q4E1H2	41,2	6	11	14	1208,2
Trans-sialidase, putative (Fragment)	Tc00,1047053510049,10	Q4CN26	67,6	6,1	1	15	1206,4

Trans-sialidase, putative (Fragment)	Tc00,1047053459061,10	Q4CLG7	28,1	9,3	2	14	1189,3
Trans-sialidase, putative (Fragment)	Tc00,1047053509417,9	Q4CW35	70,8	6,6	2	15	1188,6
Trans-sialidase, putative (Fragment)	Tc00,1047053401569,10	Q4CL29	38,5	5,3	2	9	940,7
Trans-sialidase, putative (Fragment)	Tc00,1047053509765,129	Q4DBF0	81,2	6	1	14	936,4
Trans-sialidase, putative (Fragment)	Tc00,1047053507035,11	Q4DNL8	60,5	7,2	2	13	759,4
Trans-sialidase, putative (Fragment)	Tc00,1047053455171,9	Q4CKG0	44,5	5,9	2	12	759,2
Trans-sialidase, putative (Fragment)	Tc00,1047053506231,10	Q4CUC3	35,7	5	1	7	740,1
Trans-sialidase, putative (Fragment)	Tc00,1047053505155,4	Q4CPN2	27,8	8,7	2	6	685,6
Trans-sialidase, putative (Fragment)	Tc00,1047053511197,10	Q4CWB0	66,2	5,3	1	13	651,9
Trans-sialidase, putative (Fragment)	Tc00,1047053511855,10	Q4D6K1	46,7	8,9	3	11	649,5
Trans-sialidase, putative (Fragment)	Tc00,1047053506413,89	Q4CXV0	76,7	6,1	5	7	611,1
Trans-sialidase, putative (Fragment)	Tc00,1047053426675,9	Q4CKC3	29,9	7,1	2	10	578,2
Trans-sialidase, putative (Fragment)	Tc00,1047053510843,19	Q4CQ58	28,4	9,5	1	4	565,6
Trans-sialidase, putative (Fragment)	Tc00,1047053507121,11	Q4D111	78,8	8,7	8	12	514
Trans-sialidase, putative (Fragment)	Tc00,1047053506653,10	Q4CRR6	28,4	8,8	4	6	502,5
Trans-sialidase, putative (Fragment)	Tc00,1047053506003,39	Q4CQC9	66,5	7,6	1	7	475,2
Trans-sialidase, putative (Fragment)	Tc00,1047053432995,9	Q4CKL4	48,3	5,9	1	6	407,8
Trans-sialidase, putative (Fragment)	Tc00,1047053506377,10	Q4CNE7	20,1	4,2	1	6	295
Trans-sialidase, putative (Fragment)	Tc00,1047053423205,10	Q4CL03	15	8,5	1	2	269,4
Trans-sialidase, putative (Fragment)	Tc00,1047053423707,10	Q4CKC5	33,2	6,6	2	4	230,6
Trans-sialidase, putative (Fragment)	Tc00,1047053416041,14	Q4CTI7	64,2	7,1	1	4	197,3
Trans-sialidase, putative (Fragment)	Tc00,1047053511101,11	Q4CVW4	18,9	7,7	3	2	189,4
Trans-sialidase, putative (Fragment)	Tc00,1047053507997,14	Q4D5S4	61,5	9,1	1	5	181,6
Trans-sialidase, putative (Fragment)	Tc00,1047053508849,11	Q4CLZ0	14,6	11	1	1	153,1
Trans-sialidase, putative (Fragment)	Tc00,1047053507995,20	Q4CNP3	31	4,5	1	1	152,4
Trans-sialidase, putative (Fragment)	Tc00,1047053511595,59	Q4CZB9	29	9,1	1	2	53,9
Uncharacteristic							
Uncharacterized protein	Tc00,1047053507511,50	Q4D3H5	16,7	5,9	1	4	443,6
Uncharacterized protein	Tc00,1047053504277,20	Q4CNL1	30,9	6,9	1	5	430,8
Uncharacterized protein	Tc00,1047053506871,190	Q4DNJ6	16,8	6,3	1	4	428,8
Uncharacterized protein	Tc00,1047053511675,3	Q4CUB2	36,5	4,6	1	2	337,6
Uncharacterized protein	Tc00,1047053511735,70	Q4CUQ4	23	6,2	1	3	319,9
Uncharacterized protein	Tc00,1047053509601,140	Q4DK71	20,9	8,4	1	3	301,5

Uncharacterized protein	Tc00,1047053509047,40	Q4D6D8	21,1	6,9	2	2	285,7
Uncharacterized protein (Fragment)	Tc00,1047053510717,10	Q4CTF0	21,3	7,5	4	2	229,2
Uncharacterized protein (Fragment)	Tc00,1047053511491,170	Q4DHN4	38,1	5,6	2	3	223
Uncharacterized protein	Tc00,1047053504087,20	Q4D1D9	323	5,8	2	8	218,4
Uncharacterized protein (Fragment)	Tc00,1047053506701,29	Q4CQX1	41,3	5,5	1	3	209
Uncharacterized protein	Tc00,1047053511521,27	Q4CPX4	23,1	5,8	1	2	187,2
Uncharacterized protein	Tc00,1047053511621,120	Q4DT54	23,6	5,9	2	2	184,4
Uncharacterized protein	Tc00,1047053509767,140	Q4DSC0	18,1	8,9	1	2	176,6
Uncharacterized protein	Tc00,1047053510433,10	Q4CPM9	112,1	10,1	2	2	169,6
Uncharacterized protein	Tc00,1047053510225,40	Q4CXU6	104,4	5	1	1	152,8
Uncharacterized protein	Tc00,1047053510053,30	Q4D472	14,8	4,8	1	1	152,8
Uncharacterized protein	Tc00,1047053511367,290	Q4DYG6	28,1	9,3	1	1	152,8
Uncharacterized protein	Tc00,1047053510257,20	Q4DBL2	23	9,3	1	1	152,3
Uncharacterized protein	Tc00,1047053504001,20	Q4CW22	13	7,8	1	3	110
Uncharacterized protein	Tc00,1047053511523,20	Q4D4V4	13,1	5,6	1	1	101,4
Uncharacterized protein	Tc00,1047053511743,30	Q4CTP1	50,7	6,7	1	1	99,4
Uncharacterized protein	Tc00,1047053506441,20	Q4CSI2	123,4	5,2	1	1	96,7
Uncharacterized protein	Tc00,1047053504199,20	Q4CVJ1	16,4	9,3	2	3	94,3
Uncharacterized protein	Tc00,1047053506753,230	Q4DLW7	108,1	8,9	2	3	93,8
Uncharacterized protein	Tc00,1047053509233,170	Q4DTX8	54,8	8,6	1	3	91,4
Uncharacterized protein	Tc00,1047053506885,370	Q4E4G4	26	6,9	2	1	91
Uncharacterized protein	Tc00,1047053506635,130	Q4DIP8	16,3	9	2	3	88,4
Uncharacterized protein	Tc00,1047053504001,10	Q4CW23	13,5	9,1	2	2	84,7
Uncharacterized protein	Tc00,1047053509253,10	Q4CX39	77,5	9,6	3	1	82,4
Uncharacterized protein	Tc00,1047053506993,140	Q4DN18	31,5	6,4	1	1	78
Putative uncharacterized protein	Tc00,1047053506471,19	Q4DKM7	17,6	5,7	1	2	73,3
Uncharacterized protein	Tc00,1047053511649,50	Q4DL12	64,5	5,8	1	1	71,9
Uncharacterized protein	Tc00,1047053507707,20	Q4CNH1	66	6,5	1	1	71,1
Uncharacterized protein	Tc00,1047053510877,40	Q4DPV6	24,4	9,7	2	2	64,3
Uncharacterized protein	Tc00,1047053506605,120	Q4DUX0	22,3	4,7	1	1	64,1
Uncharacterized protein	Tc00,1047053504035,100	Q4DI81	95,2	9,8	5	2	62,9
Uncharacterized protein	Tc00,1047053509615,50	Q4D2F7	20,8	4,6	1	2	60,3
Uncharacterized protein	Tc00,1047053509795,70	Q4D3I6	57,1	9,7	1	1	60,1

Uncharacterized protein	Tc00,1047053507625,120	Q4DPE4	16,9	9,6	2	2	59,1
Uncharacterized protein	Tc00,1047053510407,40	Q4D7E1	46,7	5,9	2	1	52,6
Uncharacterized protein	Tc00,1047053511733,90	Q4D6Q6	13	6,3	1	1	51,9
Uncharacterized protein	Tc00,1047053510603,169	Q4DLY9	27,3	6,2	2	1	51
Uncharacterized protein	Tc00,1047053509429,80	Q4E0M2	101,4	5,6	1	2	38,9
Heat Schock Proteins and Chaperones							
Heat shock protein 85, putative	Tc00,1047053507713,30	Q4CQS6	80,7	5,1	4	10	1062,8
10 kDa heat shock protein, putative	Tc00,1047053508209,120	Q4DFA8	10,7	9	3	4	283,5
Chaperonin containing T-complex protein, putative	Tc00,1047053511725,250	Q4DWG6	59,1	5	1	1	58,6
Chaperonin HSP60, mitochondrial	Tc00,1047053507641,290	Q4DYP5	59,1	5,4	4	6	217,7
Glucose-regulated protein 78, putative	Tc00,1047053506585,40	Q4D620	71,3	5,1	1	3	688,6
Heat shock 70 kDa protein, mitochondrial, putative	Tc00,1047053507029,30	Q4CVR9	70,9	5,8	4	3	221,8
Heat shock 70 kDa protein, putative (Fragment)	Tc00,1047053510439,61	Q4CU95	40,8	6,2	2	20	1261,6
Heat shock 70 kDa protein, putative (Fragment)	Tc00,1047053506135,9	Q4DAZ6	30,1	6,2	1	7	778,1
Heat shock protein 70 (HSP70), putative	Tc00,1047053511211,160	Q4DTM9	70,9	5,8	4	19	1716,5
Signaling							
IgE-dependent histamine-releasing factor, putative	Tc00,1047053506207,50	Q4CW52	19,6	4,5	2	3	281,1
14-3-3 protein, putative	Tc00,1047053508851,180	Q4DJB6	29,9	5	1	4	219,1
14-3-3 protein, putative	Tc00,1047053506775,80	Q4DRH6	29,1	5	2	2	171,7
Adenosine kinase, putative	Tc00,1047053509965,370	Q4E0S4	37,8	5,1	2	1	64,1
Calcium-binding protein, putative	Tc00,1047053509391,10	Q4D1Q2	23,7	4,9	9	8	415,8
Calmodulin, putative (Fragment)	Tc00,1047053506389,79	Q4D2S5	9,5	3,9	4	3	192,4
Inositol-1,4,5-trisphosphate 5-phosphatase, putative	Tc00,1047053510101,130	Q4E3P6	63,2	6,3	1	1	64,6
Mitogen-activated protein kinase, putative	Tc00,1047053511491,80	Q4DHP2	57,9	5,9	1	1	152,8
Nucleoside diphosphate kinase	Tc00,1047053508707,200	Q4E256	16,9	8,5	1	3	294,2
Phosphoglycerate kinase	Tc00,1047053511419,40	Q4D193	44,4	6,2	4	2	43,5
Protein kinase, putative	Tc00,1047053507991,20	Q4DJF7	47,9	6,6	1	1	92,3
Protein kinase, putative	Tc00,1047053508641,170	Q4DZH9	42,9	6,2	1	1	55
Ras-related protein rab-2a, putative (Fragment)	Tc00,1047053506425,169	Q4DM40	10,5	9,3	2	2	203
Ras-related protein rab-5, putative (Fragment)	Tc00,1047053511269,4	Q4D504	20,4	5,3	2	1	152,1
Serine/threonine-protein phosphatase (Fragment)	Tc00,1047053508707,340	Q4E243	17,9	5,3	2	2	206,6
Serine/threonine-protein phosphatase	Tc00,1047053508815,110	Q4DMJ3	34,4	5,5	1	3	150,5

Serine/threonine-protein phosphatase	Tc00,1047053510687,40	Q4D9Y4	35	5,2	1	1	138,7
Transcription an protein synthesis							
60S ribosomal protein L11, putative	Tc00,1047053508197,10	Q4CNF4	22,2	10,1	1	2	285,7
Elongation factor 1-alpha	Tc00,1047053511369,10	Q4CRF7	48,3	8,9	8	5	368,7
Elongation factor 1-alpha (Fragment)	Tc00,1047053510119,9	Q4CXI2	42,8	7,6	8	3	223,2
Elongation factor 2, putative	Tc00,1047053510963,90	Q4D3T1	94,1	5,7	5	6	496,6
Ribosomal protein L3, putative	Tc00,1047053510879,110	Q4DJX1	48,5	11,1	2	3	220,5
40S ribosomal protein S18, putative	Tc00,1047053506679,100	Q4E093	17,5	10,8	1	1	152,8
40S ribosomal protein S15a, putative	Tc00,1047053506297,330	Q4E0N6	14,7	9,8	3	1	152,8
Eukaryotic release factor 3, putative	Tc00,1047053506297,300	Q4E0N9	74,9	6,9	2	1	64,6
60S ribosomal protein L27A/L29, putative	Tc00,1047053508461,500	Q4E4Q8	16,2	10,5	1	1	166,3
Proteolysis							
Amastin, putative	Tc00,1047053507485,130	Q4DDC5	19,5	9	1	1	152,7
Calpain-like cysteine peptidase, putative	Tc00,1047053509003,40	Q4CW63	15,4	9,3	1	2	88,8
Calpain-like cysteine peptidase, putative	Tc00,1047053508675,29	Q4D066	12,8	5,1	2	2	43,5
Cruzipain, putative	Tc00,1047053507603,270	Q4DW02	49,9	5,7	3	3	117
Cysteine peptidase C , putative	Tc00,1047053511827,100	Q4DQB0	36,7	5,8	2	2	97,2
Cysteine peptidase, putative	Tc00,1047053507537,20	Q4CV00	46,8	5,5	3	2	55,7
Cysteine peptidase, putative	Tc00,1047053509429,320	Q4E0J7	49,8	5,4	6	3	242,3
Proteasome activator protein PA26, putative (Fragment)	Tc00,1047053503841,10	Q4D086	24,6	5,5	3	2	175,6
Proteasome regulatory ATPase subunit 1, putative	Tc00,1047053506857,90	Q4D9J1	48,5	7,1	1	1	60,7
Proteasome regulatory ATPase subunit 2, putative	Tc00,1047053511047,50	Q4D0B9	49	5,5	1	3	85,4
Proteasome regulatory ATPase subunit 3, putative	Tc00,1047053509429,270	Q4E0K2	44,7	6,7	9	1	139,6
Proteasome regulatory ATPase subunit, putative	Tc00,1047053504147,200	Q4DZM5	45,4	8,8	1	2	318,9
Proteasome regulatory non-ATPase subunit 11, putative (Fragment)	Tc00,1047053503697,9	Q4DKG4	29,5	7,8	2	1	114,9
Proteasome subunit alpha type	Tc00,1047053511145,43	Q4D1H4	27,1	4,9	2	1	152,7
Serine carboxypeptidase (CBP1), putative	Tc00,1047053508671,20	Q4CMQ4	59,5	6,8	2	13	844,7
Serine carboxypeptidase (CBP1), putative	Tc00,1047053509695,210	Q4DTP7	51,5	5	2	11	566,7
Serine carboxypeptidase S28, putative	Tc00,1047053507681,200	Q4DW34	53,8	9,2	1	3	83,3
Serine carboxypeptidase S28, putative	Tc00,1047053506425,10	Q4DM56	72,1	6,2	3	5	386,1
Carbohydrate metabolism							

Fructose-bisphosphate aldolase	Tc00,1047053504163,50	Q4D4R9	40,8	8,8	4	2	73,7
Enolase, putative	Tc00,1047053504105,140	Q4DZ98	46,4	5,9	1	9	786,2
Glucose-6-phosphate 1-dehydrogenase (Fragment)	Tc00,1047053506953,49	Q4CZI4	31,4	7,6	2	1	126,6
Glyceraldehyde 3-phosphate dehydrogenase, putative	Tc00,1047053511461,14	Q4D3Y9	14,7	11,4	6	2	182,6
Glyceraldehyde 3-phosphate dehydrogenase, putative	Tc00,1047053509065,60	Q4DHF0	39	9	2	2	133,9
Isocitrate dehydrogenase [NADP]	Tc00,1047053511575,60	Q4DG65	48,7	8,8	1	1	167,3
Isocitrate dehydrogenase [NADP]	Tc00,1047053506925,319	Q4E4L7	46,8	7,7	1	1	164,4
Lysosomal alpha-mannosidase, putative	Tc00,1047053506195,120	Q4DXL4	111,2	6,5	2	5	108,8
Malate dehydrogenase (Fragment)	Tc00,1047053507883,109	Q4D4A0	31,5	7,6	2	3	218,3
Phosphomannomutase-like protein, putative	Tc00,1047053508257,70	Q4DX52	65	6	2	1	98,1
Oxydo-Reduction							
2,4-dienoyl-coa reductase FADH1, putative	Tc00,1047053509941,100	Q4DKB3	76,2	9	1	1	138
C-1-tetrahydrofolate synthase, cytoplasmic, putative	Tc00,1047053511517,60	Q4DMU5	70,4	6,8	2	1	84,3
Cytochrome c, putative	Tc00,1047053506949,50	Q4D480	12,2	9,4	1	3	135,4
Cytochrome c, putative	Tc00,1047053508959,4	Q4CV48	12,2	9,6	1	2	126,7
Glutamate dehydrogenase	Tc00,1047053508111,30	Q4D5C2	45	8	2	6	424,7
Glutathione peroxidase	Tc00,1047053503899,119	Q4DEJ5	19,7	9	5	6	575
NAD/FAD dependent dehydrogenase, putative	Tc00,1047053505843,40	Q4CVH0	43	5,3	1	1	152,1
P450 reductase, putative	Tc00,1047053506931,80	Q4D2L9	25,5	8,8	2	2	37,9
Superoxide dismutase	Tc00,1047053511715,10	Q4D5A6	23,3	7,1	4	3	301,3
Tryparedoxin peroxidase, putative	Tc00,1047053507259,10	Q4CM56	22,4	6,3	4	8	881,8
Tryparedoxin peroxidase, putative	Tc00,1047053487507,10	Q4CVR5	22,4	6,3	4	8	552,9
Tryparedoxin peroxidase, putative	Tc00,1047053509499,14	Q4CX87	25,5	7,6	1	2	305,4
Tryparedoxin, putative	Tc00,1047053509997,30	Q4D1B8	16	5,2	1	6	486
Trafficking and membrane fusion							
Rab1 small GTP-binding protein, putative	Tc00,1047053503689,4	Q4CV38	24,6	8,7	1	2	240,1
ADP-ribosylation factor 1, putative	Tc00,1047053508415,40	Q4D7Y8	20,7	8,5	2	7	922,4
ADP-ribosylation factor 3, putative	Tc00,1047053504433,10	Q4D2I4	20,1	5,9	1	1	152,6
ADP-ribosylation factor, putative	Tc00,1047053511469,24	Q4DCJ0	20,9	8,8	2	1	152,8
Dynein light chain, putative	Tc00,1047053506925,104	Q4E4N7	10,4	6,3	1	1	152,1
Rab7 GTP binding protein, putative	Tc00,1047053508461,270	Q4E4T4	23,9	5,8	1	2	303,8
Small GTP-binding protein Rab1, putative	Tc00,1047053503715,30	Q4CZR0	22,8	7,6	2	2	314,1

Small Rab GTP binding protein, putative	Tc00,1047053506227,180	Q4DSV2	22,1	8,9	1	1	74,6
Transitional endoplasmic reticulum ATPase, putative	Tc00,1047053509733,170	Q4DWB5	86,1	5,4	1	11	1336,8
Protein and Amino Acid metabolism							
Adenosylhomocysteinase	Tc00,1047053511229,50	Q4D455	48,4	6,2	2	2	74,4
Arginine kinase, putative	Tc00,1047053507241,30	Q4CWA5	40,2	6,3	2	9	852,6
Aspartate aminotransferase	Tc00,1047053503679,10	Q4CRK6	44,9	7,1	2	2	193,6
ATP synthase subunit beta	Tc00,1047053509233,180	Q4DTX7	55,7	5,3	1	2	157,4
Seryl-tRNA synthetase, putative (Fragment)	Tc00,1047053511163,10	Q4CW46	25,7	5,5	2	1	153,1
Threonyl-tRNA synthetase, putative	Tc00,1047053508299,80	Q4D9G6	92,6	6,4	2	1	152,4
Tryptophanyl-tRNA synthetase, putative	Tc00,1047053510647,30	Q4CTS7	44,3	6,6	1	2	42,4
Ubiquitin-activating enzyme E1, putative	Tc00,1047053504427,250	Q4DYM1	114,3	5,6	2	1	152,4
Ubiquitin-conjugating enzyme E2, putative	Tc00,1047053508137,30	Q4CTN0	17,5	7,9	2	2	138,9
Ubiquitin-conjugating enzyme E2, putative	Tc00,1047053508303,4	Q4DE00	16,8	6,1	1	2	126,7
Cell Structure and Motility							
Actin, putative	Tc00,1047053510127,79	Q4D7A6	38,1	5,4	3	2	170,4
Alpha tubulin, putative	Tc00,1047053411235,9	Q4CLA1	49,8	4,9	1	23	2188,8
Beta tubulin, putative	Tc00,1047053506563,40	Q4DQP2	49,7	4,7	1	21	1962,2
Cofilin/actin depolymerizing factor, putative	Tc00,1047053510145,20	Q4CVE9	15,7	5,7	1	4	134,8
Cofilin/actin depolymerizing factor, putative	Tc00,1047053508411,10	Q4D8D3	15,7	5,7	1	3	112,4
Microtubule-associated protein, putative (Fragment)	Tc00,1047053507447,19	Q4CMT2	85,2	4,8	2	4	262
Surface protein TolT (Fragment)	Tc00,1047053508767,10	Q4CM39	24,8	9,4	2	5	437,1
Surface protein TolT, putative	Tc00,1047053504277,11	Q4CNL2	21,5	8,8	1	5	362
Surface protein TolT, putative	Tc00,1047053504157,130	Q4DGM6	33,3	7,5	1	4	259,9
Surface protein TolT	Tc00,1047053506617,20	Q4D0C6	33	8,7	3	4	259,3
Surface protein TolT	Tc00,1047053508767,20	Q4CM38	33	8,5	1	3	214
Nucleic acids binding							
S-adenosylmethionine synthase	Tc00,1047053506945,160	Q4CSC4	43,5	6	1	1	152,6
Arginyl-tRNA synthetase, putative	Tc00,1047053508355,320	Q4E397	78,8	6,1	1	1	147,7
DNA damage repair protein, putative	Tc00,1047053508625,90	Q4DBR5	68,8	6,7	2	2	45,6
GTP-binding nuclear protein rtb2, putative	Tc00,1047053509455,80	Q4DIB9	24,4	6,9	2	4	192,5
Helicase, putative	Tc00,1047053510655,30	Q4DFY7	139,7	7	1	2	37,6
High mobility group protein, putative	Tc00,1047053507951,114	Q4DUS7	30,5	9,6	2	2	55,3

Histone H2B	Tc00,1047053511635,20	Q4CTD7	12,3	11,6	2	2	101,1
PIF1 helicase-like protein, putative (Fragment)	Tc00,1047053509611,10	Q4DKA5	54	5,8	2	1	98,8
RuvB-like DNA helicase, putative	Tc00,1047053510877,60	Q4DPV3	52,7	5,6	1	2	195,2
Small nuclear ribonucleoprotein Sm-F, putative	Tc00,1047053507007,74	Q4DH43	8,2	5,2	1	1	113,7
Lipid metabolism							
Lipase domain protein, putative	Tc00,1047053509999,50	Q4DKK9	139,5	6,2	1	1	151,2
Transporters							
Nuclear transport factor 2, putative	Tc00,1047053511407,10	Q4D7W2	13,9	5,5	2	2	88
V-type ATPase, A subunit, putative	Tc00,1047053506405,120	Q4DED4	67,9	5,7	2	1	86,6
Other							
Aconitase, putative	Tc00,1047053511277,290	Q4E5G5	98,6	6,5	1	1	64,6
Cysteine peptidase inhibitor	Tc00,1047053506801,80	Q4DH32	12	6,6	1	6	801,8
Cysteine peptidase inhibitor, putative	Tc00,1047053511907,200	Q4DY71	12,1	6,6	1	6	818,5
Vacuolar protein sorting 18, putative (Fragment)	Tc00,1047053419833,10	Q4CKH3	46,3	8	1	1	90,9
