# Supplementary material for: Secretome analysis of Trypanosoma cruzi by proteomics studies
Source: PLoS One. 2017 Oct 3;12(10):e0185504. doi: 10.1371/journal.pone.0185504 (PMC5626432; doi:10.1371/journal.pone.0185504)
Supplement: S2 Table — The proteins secreted have selected with LCMSMS score was over 35 and with at least two peptides identified, or with a score over 50 but with only one peptide identified. For each protein, the number of matched proteins and peptides and the highest score are described. (PDF) [file pone.0185504.s002.pdf]

S2 Table

Protein	Accession Number		MW [kDa]	pI	Matching Proteins	Matching Peptides	Highest Scores
		Uniprot					
**Host-Parasite Interaction**							
Calreticulin, putative	Tc00,1047053510685,10	Q4CPZ0	46,2	4,8	2	3	93,7
Calreticulin, putative	Tc00,1047053509011,40	Q4DDX3	46,2	4,8	2	1	58,1
Complement regulatory protein, putative	Tc00,1047053509217,160	Q4DQ07	113,7	4,9	1	13	723,2
Dispersed gene family protein 1 , putative	Tc00,1047053506415,10	Q4CZ77	354,4	7,2	194	15	957,9
Dispersed gene family protein 1 , putative	Tc00,1047053507083,20	Q4DPN1	354,1	7,3	17	17	878,7
Dispersed gene family protein 1 , putative	Tc00,1047053511767,20	Q4DKA8	359,7	5,6	1	3	192,7
Dispersed gene family protein 1 , putative	Tc00,1047053506623,60	Q4D5I6	206,4	6,1	1	3	188
Dispersed gene family protein 1 , putative	Tc00,1047053511797,54	Q4DVS3	276,5	5,7	1	3	187,7
Dispersed gene family protein 1 , putative	Tc00,1047053511469,110	Q4DCI0	328,4	5,6	3	2	178,6
Dispersed gene family protein 1 , putative	Tc00,1047053511851,10	Q4DBZ8	362,2	5,5	1	3	169,7
Dispersed gene family protein 1 , putative	Tc00,1047053511183,460	Q4E5K0	362	5,6	1	3	168,8
Dispersed gene family protein 1 , putative	Tc00,1047053507827,10	Q4D1G6	358,3	5,7	1	2	142
Dispersed gene family protein 1 , putative	Tc00,1047053506961,50	Q4DVI9	360,2	5,9	1	2	141,7
Mucin-associated surface protein , putative	Tc00,1047053506599,100	Q4E1Z5	25,5	4,4	12	2	39
Dispersed gene family protein 1 , putative	Tc00,1047053510355,10	Q4DXG8	357,6	6	1	2	39,5
Mucin-associated surface protein , putative	Tc00,1047053511401,100	Q4DDR2	45	4,5	5	1	152,3
Mucin-associated surface protein , putative	Tc00,1047053506763,260	Q4DZB7	48,6	5,2	7	3	72,2
Mucin-associated surface protein , putative	Tc00,1047053510363,320	Q4DXN7	35,2	4,3	2	2	46,5
Mucin-associated surface protein , putative	Tc00,1047053510477,100	Q4D951	27,4	4,7	3	2	67,3
Dispersed gene family protein 1 , putative	Tc00,1047053510367,10	Q4D6V4	359,9	6	1	2	54,1
Mucin-associated surface protein , putative	Tc00,1047053509905,180	Q4DR50	48,7	4,6	5	2	51,2
Mucin-associated surface protein , putative	Tc00,1047053508873,10	Q4E519	37,8	4,4	2	2	46,8
Mucin-like glycoprotein, putative	Tc00,1047053507359,20	Q4CRJ0	36,5	4,6	1	2	237,8
Mucin-like glycoprotein, putative	Tc00,1047053508741,440	Q4E332	37,9	4,9	2	8	511
Mucin-like glycoprotein, putative	Tc00,1047053508737,10	Q4DTI3	36,7	4,9	1	2	222,8

Mucin-like glycoprotein, putative	Tc00,1047053509123,10	Q4CU27	36,5	4,5	1	5	188,9
Mucin TcMUCI, putative	Tc00,1047053504167,20	Q4CSC0	17,7	9,1	1	2	64,1
Neutral sphingomyelinase activation associated factor-like protein	Tc00,1047053509767,10	Q4DSD4	90,5	5,9	2	7	534,4
Peptidyl-prolyl cis-trans isomerase	Tc00,1047053508577,140	Q4DPB9	21,1	9,1	2	4	113,4
Peptidyl-prolyl cis-trans isomerase	Tc00,1047053506925,300	Q4E4L9	18,8	8,4	1	8	486,6
Surface protease GP63, putative	Tc00,1047053505567,20	Q4CNY8	82,7	5,2	1	42	3531,6
Surface protease GP63, putative	Tc00,1047053504015,30	Q4CPE3	86,6	5,7	8	4	384,8
Surface protease GP63, putative	Tc00,1047053447135,10	Q4CPE5	75,3	5,3	4	5	648,3
Surface protease GP63, putative	Tc00,1047053510233,10	Q4CUU3	11,8	4,2	1	4	130,3
Surface protease GP63, putative	Tc00,1047053510139,20	Q4D273	90,8	5,7	1	26	1851,2
Surface protease GP63, putative	Tc00,1047053511117,50	Q4D292	99,4	5,9	1	39	3670,9
Surface protease GP63, putative	Tc00,1047053508071,50	Q4D5G1	82,7	5,2	11	53	5209,9
Surface protease GP63, putative	Tc00,1047053503479,60	Q4D6Z1	59,9	6,2	1	27	1421,5
Surface protease GP63, putative	Tc00,1047053510477,10	Q4D958	38,6	4,6	1	2	141,1
Surface protease GP63, putative	Tc00,1047053507559,110	Q4DAG2	70,5	6,1	1	29	1613,7
Surface protease GP63, putative	Tc00,1047053509369,50	Q4DCQ9	48,4	8,9	1	13	1176
Surface protease GP63, putative	Tc00,1047053511257,100	Q4DGK2	85,3	5,7	1	8	959,6
Surface protease GP63, putative	Tc00,1047053511257,70	Q4DGK5	85,3	5,3	9	11	1051,2
Surface protease GP63, putative	Tc00,1047053511153,40	Q4DHC2	82,4	6,5	10	32	1755,6
Surface protease GP63, putative	Tc00,1047053509259,40	Q4DM64	82,8	5	1	40	3337,5
Surface protease GP63, putative	Tc00,1047053506779,180	Q4DMD2	80,9	6,5	1	22	1497,9
Surface protease GP63, putative	Tc00,1047053510503,100	Q4DN56	72,6	5,4	5	6	511,9
Surface protease GP63, putative	Tc00,1047053506289,170	Q4DVY6	76,7	5,3	2	15	1071
Surface protease GP63, putative	Tc00,1047053506289,140	Q4DVY9	88,4	5,2	1	15	1105,5
Surface protease GP63, putative	Tc00,1047053508365,100	Q4DWF5	83,5	5,7	1	5	490,5
Surface protease GP63, putative	Tc00,1047053510303,140	Q4DZ07	81,1	6,3	3	22	1628,6
Surface protease GP63, putative	Tc00,1047053510303,20	Q4DZ17	68,3	6,3	2	26	1420,8
Surface protease GP63, putative	Tc00,1047053510307,10	Q4E0N3	90,4	5,6	3	28	2005,3
Surface protease GP63, putative	Tc00,1047053508165,310	Q4E1H6	78,8	5,4	1	4	381,5

Surface protease GP63, putative	Tc00,1047053506855,220	Q4E1S2	60,2	6,1	1	22	1995,8
Surface protease GP63, putative (Fragment)	Tc00,1047053504755,10	Q4CM26	40	4,6	3	17	1240,5
Surface protease GP63, putative (Fragment)	Tc00,1047053511703,19	Q4CMY9	32,9	5,9	3	16	1048,6
Surface protease GP63, putative (Fragment)	Tc00,1047053509325,4	Q4CNB7	21,3	7,5	1	11	999,3
Surface protease GP63, putative (Fragment)	Tc00,1047053508057,11	Q4CNJ0	20,9	8,6	1	15	1243,4
Surface protease GP63, putative (Fragment)	Tc00,1047053509373,39	Q4CP23	24,4	8,9	1	13	922,4
Surface protease GP63, putative	Tc00,1047053511203,29	Q4CTY9	37,7	8,7	3	2	36,7
Surface protease GP63, putative (Fragment)	Tc00,1047053506057,101	Q4D148	22,1	8,4	1	16	1402,4
Trans-sialidase, putative	Tc00,1047053511615,10	Q4CMQ8	62,2	6,6	1	14	729,4
Trans-sialidase, putative	Tc00,1047053504145,10	Q4CP42	81	6,3	1	8	443,3
Trans-sialidase, putative	Tc00,1047053503403,20	Q4CPD1	86,6	8,3	2	18	1287,6
Trans-sialidase, putative	Tc00,1047053510907,10	Q4CPF9	103,3	5	3	19	1464,5
Trans-sialidase, putative	Tc00,1047053511835,10	Q4CPQ2	110,5	5	2	20	1318,7
Trans-sialidase, putative	Tc00,1047053509333,10	Q4CPQ3	108,9	4,8	1	21	790,6
Trans-sialidase, putative	Tc00,1047053507879,10	Q4CPT6	86,4	5,8	1	15	1201,7
Trans-sialidase, putative	Tc00,1047053507411,10	Q4CPU0	97,5	5,1	3	4	366,4
Trans-sialidase, putative	Tc00,1047053398477,10	Q4CPW8	85,6	8,2	1	50	4196,9
Trans-sialidase, putative	Tc00,1047053463279,20	Q4CPX0	80,7	6,3	2	30	2206,4
Trans-sialidase, putative	Tc00,1047053510569,10	Q4CPY4	49,7	5,5	2	24	1821,7
Trans-sialidase, putative	Tc00,1047053506353,10	Q4CQ38	82,7	6,5	1	9	417,7
Trans-sialidase, putative	Tc00,1047053510093,20	Q4CQ85	113,7	4,9	2	14	1160,5
Trans-sialidase, putative	Tc00,1047053508103,30	Q4CQL8	92,3	6,7	5	7	451,7
Trans-sialidase, putative	Tc00,1047053506397,10	Q4CQP5	81	5,8	1	16	833,9
Trans-sialidase, putative	Tc00,1047053472357,10	Q4CQY9	88	7,5	30	20	1078,2
Trans-sialidase, putative	Tc00,1047053503993,10	Q4CQZ7	76,6	8,7	1	17	1398,1
Trans-sialidase, putative	Tc00,1047053504183,20	Q4CR00	88,3	5,4	2	15	1179,2
Trans-sialidase, putative	Tc00,1047053508171,10	Q4CR72	111,3	4,9	1	12	736,5
Trans-sialidase, putative	Tc00,1047053511669,10	Q4CRD4	113	4,8	1	20	1371,1
Trans-sialidase, putative	Tc00,1047053509411,10	Q4CRJ5	89,4	5,4	5	21	1213,4
Trans-sialidase, putative	Tc00,1047053505231,20	Q4CRR3	84,2	6,6	2	5	310,4

Trans-sialidase, putative	Tc00,1047053507283,10	Q4CRR8	78,6	9	2	10	412,5
Trans-sialidase, putative	Tc00,1047053503907,10	Q4CRT3	99,6	4,9	2	42	2950,1
Trans-sialidase, putative	Tc00,1047053511833,10	Q4CRY9	112,5	4,8	1	17	1199
Trans-sialidase, putative	Tc00,1047053509185,30	Q4CRZ2	85,9	6,9	2	11	541,9
Trans-sialidase, putative	Tc00,1047053507759,10	Q4CRZ9	113	4,8	2	36	2276,4
Trans-sialidase, putative	Tc00,1047053504341,10	Q4CSD6	99,9	5,8	4	18	1331,9
Trans-sialidase, putative	Tc00,1047053509629,10	Q4CSG0	101,3	4,8	4	67	5848,3
Trans-sialidase, putative	Tc00,1047053509513,10	Q4CSG5	81,6	8,7	1	25	1480,6
Trans-sialidase, putative	Tc00,1047053507427,10	Q4CSI1	88,8	5,3	1	42	3389,4
Trans-sialidase, putative	Tc00,1047053509281,20	Q4CSS2	79,2	5,9	3	23	1523,6
Trans-sialidase, putative	Tc00,1047053503601,10	Q4CSS7	85,3	8,7	1	6	323,6
Trans-sialidase, putative	Tc00,1047053508563,20	Q4CSV0	90,1	5,1	2	17	937,5
Trans-sialidase, putative	Tc00,1047053506427,10	Q4CSV1	106,5	4,8	3	43	3465,8
Trans-sialidase, putative	Tc00,1047053504479,10	Q4CSZ4	93,4	5,3	2	28	2016,6
Trans-sialidase, putative	Tc00,1047053504343,10	Q4CT07	85,7	5,7	1	11	584,7
Trans-sialidase, putative	Tc00,1047053507125,10	Q4CTA6	85,8	8,7	1	25	2338
Trans-sialidase, putative	Tc00,1047053506021,20	Q4CTT0	85,7	5,9	1	12	979
Trans-sialidase, putative	Tc00,1047053509931,20	Q4CTU4	113,5	4,8	2	26	2318,9
Trans-sialidase, putative	Tc00,1047053506841,20	Q4CTX4	85,9	5,6	1	27	1915,3
Trans-sialidase, putative	Tc00,1047053506455,30	Q4CUA7	81,9	6,9	4	16	736
Trans-sialidase, putative	Tc00,1047053509377,20	Q4CUE8	85,7	5,2	1	26	1925
Trans-sialidase, putative	Tc00,1047053503759,10	Q4CUS9	81,7	8,9	1	30	1895,1
Trans-sialidase, putative	Tc00,1047053509387,10	Q4CUT1	112,3	5,1	1	22	1364,4
Trans-sialidase, putative	Tc00,1047053509187,10	Q4CUU4	81,8	9,2	2	20	853,3
Trans-sialidase, putative	Tc00,1047053510331,10	Q4CUZ0	106,2	5	1	43	2521,5
Trans-sialidase, putative	Tc00,1047053420293,20	Q4CVS4	79,6	5,4	1	9	500,5
Trans-sialidase, putative	Tc00,1047053510787,10	Q4CVS5	124,8	5,8	3	47	3644,7
Trans-sialidase, putative	Tc00,1047053506217,40	Q4CVZ4	113,6	5	2	28	1825,1
Trans-sialidase, putative	Tc00,1047053507313,20	Q4CW78	112,8	5	1	22	1559
Trans-sialidase, putative	Tc00,1047053508455,20	Q4CWC5	95,1	5,1	2	5	299,6

Trans-sialidase, putative	Tc00,1047053507473,30	Q4CWN6	81,3	5,2	2	15	901,9
Trans-sialidase, putative	Tc00,1047053504101,50	Q4CX14	82,5	8,7	27	9	505,1
Trans-sialidase, putative	Tc00,1047053509533,10	Q4CXJ1	80,8	5,1	1	13	944
Trans-sialidase, putative	Tc00,1047053507839,40	Q4CXS5	80,8	8,4	1	44	3875,6
Trans-sialidase, putative	Tc00,1047053511779,60	Q4CXY5	101,1	4,9	1	42	3066,5
Trans-sialidase, putative	Tc00,1047053511551,10	Q4CYA8	85,9	5,2	6	28	2736
Trans-sialidase, putative	Tc00,1047053509843,20	Q4CYS5	95	5,7	1	40	3050
Trans-sialidase, putative	Tc00,1047053506341,50	Q4CYW2	112,2	4,8	1	28	1623
Trans-sialidase, putative	Tc00,1047053509495,30	Q4CZ79	99,4	6	5	49	4088
Trans-sialidase, putative	Tc00,1047053507753,10	Q4CZ80	113,8	5,1	1	30	1483
Trans-sialidase, putative	Tc00,1047053511057,40	Q4CZ90	84	5,3	2	10	560,5
Trans-sialidase, putative	Tc00,1047053508581,40	Q4CZ95	75,4	6,8	2	29	1921,8
Trans-sialidase, putative	Tc00,1047053508581,10	Q4CZ96	90,7	5,7	1	11	499,2
Trans-sialidase, putative	Tc00,1047053506723,20	Q4CZC3	93,3	5,8	2	24	1589,4
Trans-sialidase, putative	Tc00,1047053507979,30	Q4CZE6	89,4	8,4	3	15	925,9
Trans-sialidase, putative	Tc00,1047053507773,10	Q4CZJ4	98,4	5,8	1	2	79,4
Trans-sialidase, putative	Tc00,1047053509685,40	Q4D095	93,7	5,3	6	6	463,7
Trans-sialidase, putative	Tc00,1047053505993,30	Q4D0D9	111,9	4,8	1	26	1786,6
Trans-sialidase, putative	Tc00,1047053509115,20	Q4D0F0	93,4	5	1	3	202,1
Trans-sialidase, putative	Tc00,1047053510125,20	Q4D0T2	113,6	4,8	2	27	1424
Trans-sialidase, putative	Tc00,1047053507121,20	Q4D110	89,1	5,4	1	41	2670,9
Trans-sialidase, putative	Tc00,1047053508627,40	Q4D1A3	113,8	4,9	2	52	4477,9
Trans-sialidase, putative	Tc00,1047053510491,60	Q4D1A6	93,9	5,5	1	5	374,3
Trans-sialidase, putative	Tc00,1047053508055,30	Q4D1Z3	85,2	5,9	2	29	2534,7
Trans-sialidase, putative	Tc00,1047053510635,10	Q4D232	91	5,4	2	36	3237,3
Trans-sialidase, putative	Tc00,1047053511117,30	Q4D293	81,8	6,3	2	17	1492,7
Trans-sialidase, putative	Tc00,1047053505609,40	Q4D2F9	83,3	7,2	11	12	423,3
Trans-sialidase, putative	Tc00,1047053507255,20	Q4D2I1	85,3	6,2	1	33	2985,7
Trans-sialidase, putative	Tc00,1047053510411,10	Q4D2I7	91,9	5,2	1	18	1178,8
Trans-sialidase, putative	Tc00,1047053510979,40	Q4D2L1	91,8	5,9	5	13	688,2

Trans-sialidase, putative	Tc00,1047053505207,10	Q4D308	88,6	5,6	1	19	919,9
Trans-sialidase, putative	Tc00,1047053506603,40	Q4D349	82,4	8,5	4	8	581,3
Trans-sialidase, putative	Tc00,1047053506597,40	Q4D371	82,8	8,5	6	13	555,1
Trans-sialidase, putative	Tc00,1047053505975,20	Q4D3K3	152,1	5,6	8	27	2391,9
Trans-sialidase, putative	Tc00,1047053510205,40	Q4D3Q8	83,9	5,5	1	9	437,6
Trans-sialidase, putative	Tc00,1047053508451,60	Q4D4B5	85,8	5,3	4	18	1246,5
Trans-sialidase, putative	Tc00,1047053506171,60	Q4D5B9	74	5,4	1	19	841,6
Trans-sialidase, putative	Tc00,1047053511219,40	Q4D5K5	86,7	5,7	1	23	1822,5
Trans-sialidase, putative	Tc00,1047053507907,20	Q4D625	79	6,2	1	12	670,4
Trans-sialidase, putative	Tc00,1047053509755,10	Q4D649	82,5	5,7	3	9	447,4
Trans-sialidase, putative	Tc00,1047053507819,30	Q4D6J3	85,1	5,6	1	27	2305,9
Trans-sialidase, putative	Tc00,1047053511885,20	Q4D6K2	84,6	5,6	2	16	1064,9
Trans-sialidase, putative	Tc00,1047053509427,10	Q4D6S1	79,4	5,9	1	8	362,9
Trans-sialidase, putative	Tc00,1047053506053,50	Q4D6Y6	98,9	6,1	2	10	737,4
Trans-sialidase, putative	Tc00,1047053503447,20	Q4D7N9	82,7	8,9	1	11	559,8
Trans-sialidase, putative	Tc00,1047053508285,60	Q4D825	85,1	5,4	3	32	2100,6
Trans-sialidase, putative	Tc00,1047053509251,80	Q4D859	91,5	5	1	2	110,9
Trans-sialidase, putative	Tc00,1047053505919,20	Q4D8B3	78,6	8,8	1	9	940,5
Trans-sialidase, putative	Tc00,1047053511129,40	Q4D8H5	113,3	4,9	1	45	3347,5
Trans-sialidase, putative	Tc00,1047053511349,100	Q4D8S3	90,7	5,1	7	12	695,8
Trans-sialidase, putative	Tc00,1047053509075,50	Q4D8V4	80,2	5,6	1	19	1162,5
Trans-sialidase, putative	Tc00,1047053510403,30	Q4D980	82,2	6,1	3	12	510,6
Trans-sialidase, putative	Tc00,1047053508717,60	Q4D9H3	86,1	5,8	1	28	1743,2
Trans-sialidase, putative	Tc00,1047053511875,20	Q4DA40	99	5,1	1	36	2943,6
Trans-sialidase, putative	Tc00,1047053507059,50	Q4DBC6	96,8	4,9	12	4	100,7
Trans-sialidase, putative	Tc00,1047053504099,50	Q4DBC9	78,1	5,5	1	7	491,3
Trans-sialidase, putative	Tc00,1047053511757,70	Q4DBD3	78,9	6,2	1	12	543,9
Trans-sialidase, putative	Tc00,1047053509765,50	Q4DBF1	112,9	4,9	1	26	1859,8
Trans-sialidase, putative	Tc00,1047053506345,90	Q4DCE1	91,6	5	1	18	1135,5
Trans-sialidase, putative	Tc00,1047053507479,20	Q4DCG7	89	5,5	2	43	3865

Trans-sialidase, putative	Tc00,1047053507357,100	Q4DCY7	85,7	8,6	1	21	1115,5
Trans-sialidase, putative	Tc00,1047053505949,30	Q4DDV0	85,4	6,3	1	6	403,1
Trans-sialidase, putative	Tc00,1047053509529,40	Q4DDZ1	94,2	5,3	1	31	2188,3
Trans-sialidase, putative	Tc00,1047053508521,100	Q4DE10	119,9	4,9	1	20	1213,3
Trans-sialidase, putative	Tc00,1047053508521,20	Q4DE13	112,4	4,9	1	26	1793,2
Trans-sialidase, putative	Tc00,1047053509663,50	Q4DE15	112,4	4,7	1	25	1228,4
Trans-sialidase, putative	Tc00,1047053505365,60	Q4DEB7	113,3	5	1	42	2981,1
Trans-sialidase, putative	Tc00,1047053509785,50	Q4DEK8	87,5	6	5	10	737,2
Trans-sialidase, putative	Tc00,1047053507233,10	Q4DEP9	112,3	5	1	25	1256,8
Trans-sialidase, putative	Tc00,1047053508247,90	Q4DEZ7	87,3	5,6	6	7	219,9
Trans-sialidase, putative	Tc00,1047053510847,10	Q4DF05	108,5	4,7	1	32	2095,2
Trans-sialidase, putative	Tc00,1047053511587,90	Q4DFH1	112,9	4,8	1	34	2626,9
Trans-sialidase, putative	Tc00,1047053504425,10	Q4DFP2	108	4,8	2	39	1876,5
Trans-sialidase, putative	Tc00,1047053506129,50	Q4DFT5	159,2	6,6	3	19	1495,9
Trans-sialidase, putative	Tc00,1047053506129,30	Q4DFT6	78,8	6,1	4	20	1627,9
Trans-sialidase, putative	Tc00,1047053510095,20	Q4DGR6	83,4	6,5	1	5	181,6
Trans-sialidase, putative	Tc00,1047053506737,90	Q4DGS3	94,8	5,2	19	6	411,1
Trans-sialidase, putative	Tc00,1047053509265,120	Q4DGT1	80,5	5,5	1	17	1263,6
Trans-sialidase, putative	Tc00,1047053509265,110	Q4DGT2	185,2	6,4	2	19	1357,3
Trans-sialidase, putative	Tc00,1047053509265,90	Q4DGT3	86,2	6,1	1	39	3436,3
Trans-sialidase, putative	Tc00,1047053506975,90	Q4DGV8	112,5	4,9	1	34	2812,5
Trans-sialidase, putative	Tc00,1047053506975,80	Q4DGV9	78	5,1	3	24	1880,2
Trans-sialidase, putative	Tc00,1047053507653,20	Q4DI36	85,8	5,5	1	11	438,7
Trans-sialidase, putative	Tc00,1047053506965,170	Q4DIE5	81,2	5,4	10	17	1122,6
Trans-sialidase, putative	Tc00,1047053506001,40	Q4DII4	94	5,2	1	3	220
Trans-sialidase, putative	Tc00,1047053508045,120	Q4DIN5	108,8	4,7	1	22	916,1
Trans-sialidase, putative	Tc00,1047053510853,40	Q4DJD5	101	5,8	4	16	1366,7
Trans-sialidase, putative	Tc00,1047053508061,20	Q4DKB6	89,8	5,4	1	17	1072,5
Trans-sialidase, putative	Tc00,1047053508607,50	Q4DKL5	93,2	6,7	2	15	1136,7
Trans-sialidase, putative	Tc00,1047053506471,120	Q4DKM3	101,7	5	4	32	2766,1

Trans-sialidase, putative	Tc00,1047053508523,30	Q4DL67	87,4	5,5	1	9	500,2
Trans-sialidase, putative	Tc00,1047053509549,10	Q4DLR5	85,4	6,6	7	23	1451,4
Trans-sialidase, putative	Tc00,1047053508593,10	Q4DMW7	97,5	6	1	8	346,4
Trans-sialidase, putative	Tc00,1047053511861,50	Q4DMZ9	114,2	4,7	1	13	968,2
Trans-sialidase, putative	Tc00,1047053511861,20	Q4DN00	57,4	5,6	1	2	59,4
Trans-sialidase, putative	Tc00,1047053506967,60	Q4DP77	103,2	5	1	6	304,4
Trans-sialidase, putative	Tc00,1047053511185,80	Q4DPD2	91,7	4,9	1	3	226
Trans-sialidase, putative	Tc00,1047053507047,40	Q4DPT3	82	8,6	6	31	1704,9
Trans-sialidase, putative	Tc00,1047053507085,30	Q4DQ76	94,9	6,3	1	45	3544,8
Trans-sialidase, putative	Tc00,1047053506757,120	Q4DQA0	87,5	5,6	16	31	1746,7
Trans-sialidase, putative	Tc00,1047053506757,60	Q4DQA2	87,2	5,8	15	36	2282,4
Trans-sialidase, putative	Tc00,1047053511827,110	Q4DQA9	78,4	6,1	1	2	52,7
Trans-sialidase, putative	Tc00,1047053508871,10	Q4DQS8	83,5	8,1	49	7	360,3
Trans-sialidase, putative	Tc00,1047053511911,60	Q4DQV8	113,6	4,9	1	47	2827,2
Trans-sialidase, putative	Tc00,1047053511911,10	Q4DQW0	80,3	6,3	1	15	770,5
Trans-sialidase, putative	Tc00,1047053507953,140	Q4DR16	82,2	5,9	8	11	342,3
Trans-sialidase, putative	Tc00,1047053507953,100	Q4DR18	83,7	8,6	1	11	712,1
Trans-sialidase, putative	Tc00,1047053509905,200	Q4DR49	82	5,5	2	17	1396,2
Trans-sialidase, putative	Tc00,1047053509905,170	Q4DR51	113,1	5	1	29	1322,1
Trans-sialidase, putative	Tc00,1047053506577,80	Q4DR84	99,1	5	1	16	1371,3
Trans-sialidase, putative	Tc00,1047053511487,90	Q4DRZ7	82,9	6,5	1	12	585,6
Trans-sialidase, putative	Tc00,1047053507949,210	Q4DS03	81,9	8,2	1	18	1203,6
Trans-sialidase, putative	Tc00,1047053503861,40	Q4DSI1	112,8	4,8	2	25	1729,1
Trans-sialidase, putative	Tc00,1047053504769,100	Q4DSY8	83,3	5,8	1	17	1212,6
Trans-sialidase, putative	Tc00,1047053510025,50	Q4DTC3	93,5	8,2	2	7	588,9
Trans-sialidase, putative	Tc00,1047053509157,170	Q4DTD3	107,3	4,9	1	46	3730,4
Trans-sialidase, putative	Tc00,1047053506499,170	Q4DU07	82,9	8,8	4	13	728,1
Trans-sialidase, putative	Tc00,1047053506241,30	Q4DU42	82,3	6,2	2	8	396,1
Trans-sialidase, putative	Tc00,1047053510713,30	Q4DUP3	90,3	5,5	3	34	2832,8
Trans-sialidase, putative	Tc00,1047053510021,180	Q4DUY4	90,3	8,2	2	11	595,4

Trans-sialidase, putative	Tc00,1047053510021,120	Q4DUY7	86,9	5,7	1	25	1110,8
Trans-sialidase, putative	Tc00,1047053506331,130	Q4DV16	80,9	6,4	1	33	2804,2
Trans-sialidase, putative	Tc00,1047053506331,90	Q4DV17	83,2	5,5	1	13	709,8
Trans-sialidase, putative	Tc00,1047053506409,170	Q4DV25	98,7	4,9	1	5	203,4
Trans-sialidase, putative	Tc00,1047053506961,150	Q4DVI7	114,1	4,7	1	35	2536
Trans-sialidase, putative	Tc00,1047053506961,25	Q4DVJ1	107,8	4,7	1	30	2577,9
Trans-sialidase, putative	Tc00,1047053505997,80	Q4DWA9	111,4	4,9	2	31	1842,4
Trans-sialidase, putative	Tc00,1047053510643,40	Q4DWU9	113	4,9	2	24	1932,1
Trans-sialidase, putative	Tc00,1047053507875,220	Q4DWV1	86,1	5,3	3	21	1600,9
Trans-sialidase, putative	Tc00,1047053507875,70	Q4DWV4	94,4	5,3	1	29	2487,8
Trans-sialidase, putative	Tc00,1047053506717,80	Q4DX60	113,7	5,1	1	42	2711,2
Trans-sialidase, putative	Tc00,1047053506067,300	Q4DX98	97,5	5,2	1	7	304,9
Trans-sialidase, putative	Tc00,1047053506813,140	Q4DY88	89,5	5,5	1	25	2179,7
Trans-sialidase, putative	Tc00,1047053506683,240	Q4DYC7	80,9	6,2	1	12	854,7
Trans-sialidase, putative	Tc00,1047053506683,190	Q4DYC8	80,8	5,4	1	6	195,8
Trans-sialidase, putative	Tc00,1047053506683,110	Q4DYD0	113,7	4,8	1	46	2858
Trans-sialidase, putative	Tc00,1047053504427,230	Q4DYM3	108,6	4,8	1	50	3258,5
Trans-sialidase, putative	Tc00,1047053507237,10	Q4DYY9	81,5	6,1	1	17	863
Trans-sialidase, putative	Tc00,1047053506763,110	Q4DZC4	114,9	4,8	7	25	1027,6
Trans-sialidase, putative	Tc00,1047053506537,200	Q4DZD4	120,1	5,2	1	45	4291,2
Trans-sialidase, putative	Tc00,1047053506537,80	Q4DZD8	85,6	5,5	4	25	1672,3
Trans-sialidase, putative	Tc00,1047053507063,70	Q4E026	78	6	1	3	125,1
Trans-sialidase, putative	Tc00,1047053509875,80	Q4E0D0	81,7	8,9	3	14	971,9
Trans-sialidase, putative	Tc00,1047053508325,230	Q4E0H9	161,1	8,2	2	28	2604,3
Trans-sialidase, putative	Tc00,1047053510307,240	Q4E0M8	113,5	4,9	1	36	2203,2
Trans-sialidase, putative	Tc00,1047053508139,240	Q4E1E4	83,9	6,4	1	44	4172,4
Trans-sialidase, putative	Tc00,1047053510483,360	Q4E1F4	92,1	5,4	1	26	1328,3
Trans-sialidase, putative	Tc00,1047053511603,450	Q4E2A1	83,2	8,1	3	11	464,4
Trans-sialidase, putative	Tc00,1047053511603,90	Q4E2C0	97,2	5,4	14	10	586
Trans-sialidase, putative	Tc00,1047053509979,320	Q4E2C9	82,8	8,5	5	9	518,7

Trans-sialidase, putative	Tc00,1047053510279,320	Q4E373	98,5	5,4	2	5	266,7
Trans-sialidase, putative	Tc00,1047053508355,80	Q4E3C3	101,8	4,9	1	19	1588,4
Trans-sialidase, putative	Tc00,1047053507611,170	Q4E3G9	85,4	6,1	1	18	945
Trans-sialidase, putative	Tc00,1047053510377,330	Q4E3V0	86,9	5,4	9	23	1060,5
Trans-sialidase, putative	Tc00,1047053510377,10	Q4E3W2	83,3	8,6	8	14	743,5
Trans-sialidase, putative	Tc00,1047053511173,470	Q4E424	98,7	5	31	6	191,5
Trans-sialidase, putative	Tc00,1047053511173,440	Q4E426	82,5	5,4	43	15	719,2
Trans-sialidase, putative	Tc00,1047053511173,280	Q4E433	96,8	5,1	3	6	413,8
Trans-sialidase, putative	Tc00,1047053511173,130	Q4E440	98,9	5,1	3	12	730,5
Trans-sialidase, putative	Tc00,1047053506885,210	Q4E4H9	106,2	5	3	30	1302,4
Trans-sialidase, putative	Tc00,1047053510359,530	Q4E533	86,5	8,1	1	6	233,9
Trans-sialidase, putative	Tc00,1047053511183,430	Q4E5K1	91,9	4,8	2	5	400,6
Trans-sialidase, putative	Tc00,1047053426675,9	Q4CKC3	29,9	7,1	2	8	455,1
Trans-sialidase, putative	Tc00,1047053423707,10	Q4CKC5	33,2	6,6	3	5	514,2
Trans-sialidase, putative	Tc00,1047053455171,9	Q4CKG0	44,5	5,9	1	4	248,8
Trans-sialidase, putative	Tc00,1047053433733,10	Q4CKI9	37,8	5,5	1	14	1142,5
Trans-sialidase, putative	Tc00,1047053432995,9	Q4CKL4	48,3	5,9	1	8	234,1
Trans-sialidase, putative	Tc00,1047053402919,10	Q4CKZ3	20,6	4,2	1	2	64,6
Trans-sialidase, putative	Tc00,1047053483183,10	Q4CKZ4	58	4,8	1	10	701,2
Trans-sialidase, putative	Tc00,1047053423205,10	Q4CL03	15	8,5	1	2	174,4
Trans-sialidase, putative	Tc00,1047053401569,10	Q4CL29	38,5	5,3	2	11	1104,8
Trans-sialidase, putative	Tc00,1047053508639,10	Q4CL30	28,9	4,5	1	30	2447,6
Trans-sialidase, putative	Tc00,1047053404431,10	Q4CL45	37,8	5,1	1	4	294,1
Trans-sialidase, putative	Tc00,1047053425435,10	Q4CLC2	67,2	5	1	5	273,7
Trans-sialidase, putative	Tc00,1047053432997,10	Q4CLF9	67,2	5,9	1	37	2450,6
Trans-sialidase, putative	Tc00,1047053459061,10	Q4CLG7	28,1	9,3	2	10	729,3
Trans-sialidase, putative	Tc00,1047053508849,11	Q4CLZ0	14,6	11	1	1	75,6
Trans-sialidase, putative	Tc00,1047053510049,10	Q4CN26	67,6	6,1	1	19	1415,1
Trans-sialidase, putative	Tc00,1047053510881,9	Q4CN34	78,7	6,1	1	30	2242,1
Trans-sialidase, putative	Tc00,1047053506377,10	Q4CNE7	20,1	4,2	1	4	198,5

Trans-sialidase, putative	Tc00,1047053507995,20	Q4CNP3	31	4,5	1	3	258,9
Trans-sialidase, putative	Tc00,1047053421173,4	Q4CP99	89,5	5,8	1	24	1457,9
Trans-sialidase, putative	Tc00,1047053511831,10	Q4CPA0	110,1	4,9	1	16	860,1
Trans-sialidase, putative	Tc00,1047053505155,4	Q4CPN2	27,8	8,7	2	7	855,5
Trans-sialidase, putative	Tc00,1047053504593,10	Q4CPN3	80,5	4,9	1	28	1565,3
Trans-sialidase, putative	Tc00,1047053506515,29	Q4CPR9	29,7	8,4	1	9	639,1
Trans-sialidase, putative	Tc00,1047053510981,20	Q4CQ20	81,4	4,6	1	30	2317
Trans-sialidase, putative	Tc00,1047053510843,19	Q4CQ58	28,4	9,5	1	3	175,4
Trans-sialidase, putative	Tc00,1047053506003,39	Q4CQC9	66,5	7,6	1	11	758,9
Trans-sialidase, putative	Tc00,1047053507505,10	Q4CR17	74,8	4,8	1	52	4334,6
Trans-sialidase, putative	Tc00,1047053503441,5	Q4CSM7	38,3	8,5	1	4	432,9
Trans-sialidase, putative	Tc00,1047053506253,39	Q4CSN1	60,7	5,9	1	26	1263,3
Trans-sialidase, putative	Tc00,1047053416041,14	Q4CTI7	64,2	7,1	1	15	958,2
Trans-sialidase, putative	Tc00,1047053511123,11	Q4CTZ4	53,7	8,4	1	11	710
Trans-sialidase, putative	Tc00,1047053506231,10	Q4CUC3	35,7	5	1	8	642
Trans-sialidase, putative	Tc00,1047053510233,39	Q4CUU0	75,5	8	2	24	1726,6
Trans-sialidase, putative	Tc00,1047053511101,11	Q4CVW4	18,9	7,7	3	3	242,6
Trans-sialidase, putative	Tc00,1047053509417,9	Q4CW35	70,8	6,6	1	20	1778,3
Trans-sialidase, putative	Tc00,1047053503667,10	Q4CW39	68,5	5,1	1	5	190,4
Trans-sialidase, putative	Tc00,1047053504193,15	Q4CWT8	29,6	9,6	1	2	171,3
Trans-sialidase, putative	Tc00,1047053509031,20	Q4CX76	54,3	8,9	3	9	510,1
Trans-sialidase, putative	Tc00,1047053511105,60	Q4CX91	85,7	4,6	2	30	2468,3
Trans-sialidase, putative	Tc00,1047053506413,89	Q4CXV0	76,7	6,1	4	1	166,3
Trans-sialidase, putative	Tc00,1047053506373,39	Q4CXZ5	19,4	8,9	1	5	151,9
Trans-sialidase, putative	Tc00,1047053511595,59	Q4CZB9	29	9,1	1	2	57,2
Trans-sialidase, putative	Tc00,1047053507121,11	Q4D111	78,8	8,7	2	20	1363,1
Trans-sialidase, putative	Tc00,1047053506895,80	Q4D266	36,4	9	4	23	2010,7
Trans-sialidase, putative	Tc00,1047053510411,40	Q4D2I6	62,3	4,9	2	18	1089
Trans-sialidase, putative	Tc00,1047053508297,7	Q4D3D0	49,5	5,5	1	18	1347
Trans-sialidase, putative	Tc00,1047053511855,10	Q4D6K1	46,7	8,9	1	8	240,1

Trans-sialidase, putative	Tc00,1047053508903,110	Q4DAA5	111,5	4,8	2	52	4049,6
Trans-sialidase, putative	Tc00,1047053511757,100	Q4DBD2	62,3	8,9	2	21	1208,3
Trans-sialidase, putative	Tc00,1047053509765,129	Q4DBF0	81,2	6	1	26	1830,4
Trans-sialidase, putative	Tc00,1047053508061,154	Q4DKB4	66,6	8,6	1	22	1220,5
Trans-sialidase, putative	Tc00,1047053507035,11	Q4DNL8	60,5	7,2	2	20	1665,7
Trans-sialidase, putative	Tc00,1047053508165,430	Q4E1H2	41,2	6	7	11	538,4
**Uncharacteristic**							
Uncharacterized protein	Tc00,1047053507511,50	Q4D3H5	16,7	5,9	1	3	331,8
Uncharacterized protein	Tc00,1047053506871,190	Q4DNJ6	16,8	6,3	1	3	324,3
Uncharacterized protein	Tc00,1047053511675,3	Q4CUB2	36,5	4,6	1	6	246,7
Uncharacterized protein	Tc00,1047053509047,40	Q4D6D8	21,1	6,9	2	2	185
Uncharacterized protein	Tc00,1047053504001,10	Q4CW23	13,5	9,1	2	3	180,8
Uncharacterized protein	Tc00,1047053510433,10	Q4CPM9	112,1	10,1	2	2	166,5
Uncharacterized protein	Tc00,1047053504001,20	Q4CW22	13	7,8	1	3	105,2
Uncharacterized protein	Tc00,1047053504199,20	Q4CVJ1	16,4	9,3	2	3	99,4
Uncharacterized protein	Tc00,1047053510877,40	Q4DPV6	24,4	9,7	2	2	92,1
Uncharacterized protein	Tc00,1047053511735,70	Q4CUQ4	23	6,2	1	2	91,9
Uncharacterized protein	Tc00,1047053504087,20	Q4D1D9	323	5,8	2	2	86,4
Uncharacterized protein	Tc00,1047053510717,10	Q4CTF0	21,3	7,5	4	2	82,4
Uncharacterized protein	Tc00,1047053511521,27	Q4CPX4	23,1	5,8	1	2	78,5
Uncharacterized protein	Tc00,1047053511621,120	Q4DT54	23,6	5,9	4	2	75,8
Uncharacterized protein	Tc00,1047053507707,20	Q4CNH1	66	6,5	1	1	73,9
Uncharacterized protein	Tc00,1047053506605,120	Q4DUX0	22,3	4,7	1	1	64,1
Uncharacterized protein	Tc00,1047053506701,29	Q4CQX1	41,3	5,5	1	2	56,3
Uncharacterized protein	Tc00,1047053511491,170	Q4DHN4	38,1	5,6	2	2	52,6
Uncharacterized protein	Tc00,1047053507711,20	Q4DZS6	14,3	4,9	2	2	43,8
Uncharacterized protein	Tc00,1047053505989,110	Q4D5L9	28,7	5,2	2	2	39,3
**Heat Schock Proteins and Chaperones**							
Heat shock 70 kDa protein, mitochondrial, putative	Tc00,1047053507029,30	Q4CVR9	70,9	5,8	1	2	127,5
Heat shock 70 kDa protein, putative	Tc00,1047053510439,61	Q4CU95	40,8	6,2	2	18	1064,5

Heat shock 70 kDa protein, putative	Tc00,1047053506135,9	Q4DAZ6	30,1	6,2	1	10	896,1
Heat shock protein 70 , putative	Tc00,1047053511211,170	Q4DTM8	73,3	5,4	2	22	1705
Heat shock protein 70 , putative	Tc00,1047053511211,160	Q4DTM9	70,9	5,8	2	22	1858,6
Heat shock protein 85, putative	Tc00,1047053507713,30	Q4CQS6	80,7	5,1	4	14	1029,1
10 kDa heat shock protein, putative	Tc00,1047053508209,120	Q4DFA8	10,7	9	3	2	190,4
Chaperonin HSP60, mitochondrial	Tc00,1047053507641,290	Q4DYP5	59,1	5,4	4	5	213,2
Glucose-regulated protein 78, putative	Tc00,1047053506585,40	Q4D620	71,3	5,1	1	8	608,3
**Signaling**							
IgE-dependent histamine-releasing factor, putative	Tc00,1047053506207,50	Q4CW52	19,6	4,5	2	3	114,6
14-3-3 protein, putative	Tc00,1047053508851,180	Q4DJB6	29,9	5	1	4	173,5
14-3-3 protein, putative	Tc00,1047053506775,80	Q4DRH6	29,1	5	2	4	120,3
Calcium-binding protein, putative	Tc00,1047053509391,10	Q4D1Q2	23,7	4,9	9	8	427
Calmodulin, putative	Tc00,1047053506389,79	Q4D2S5	9,5	3,9	4	3	191,7
Nucleoside diphosphate kinase	Tc00,1047053508707,200	Q4E256	16,9	8,5	1	3	220,8
Phosphoglycerate kinase	Tc00,1047053511419,40	Q4D193	44,4	6,2	4	2	48,7
Ras-related protein rab-2a, putative	Tc00,1047053506425,169	Q4DM40	10,5	9,3	2	2	69,3
Ras-related protein rab-5, putative	Tc00,1047053511269,4	Q4D504	20,4	5,3	2	1	152,1
Serine/threonine-protein phosphatase	Tc00,1047053510687,40	Q4D9Y4	35	5,2	1	1	67,1
Pyruvate phosphate dikinase, putative	Tc00,1047053506297,190	Q4E0Q0	100,8	8,4	2	2	66,7
Serine/threonine-protein phosphatase	Tc00,1047053508815,110	Q4DMJ3	34,4	5,5	1	2	115,5
**Transcription an protein synthesis**							
40S ribosomal protein S15a, putative	Tc00,1047053506297,330	Q4E0N6	14,7	9,8	3	1	53,3
40S ribosomal protein S18, putative	Tc00,1047053506679,100	Q4E093	17,5	10,8	1	2	49,2
60S ribosomal protein L10a, putative	Tc00,1047053511233,7	Q4DN74	12,3	9,7	3	2	47,4
60S ribosomal protein L11, putative	Tc00,1047053508197,10	Q4CNF4	22,2	10,1	1	1	138,6
Elongation factor 1-alpha	Tc00,1047053510119,9	Q4CXI2	42,8	7,6	8	6	169,6
Elongation factor 2, putative	Tc00,1047053510963,90	Q4D3T1	94,1	5,7	5	5	286,1
Elongation factor 1-alpha , putative	Tc00,1047053511369,30	Q4CRF5	30,7	9	6	3	196,5
**Proteolysis**							
Thimet oligopeptidase, putative	Tc00,1047053511237,10	Q4D7D2	77,6	5,6	2	3	204,2

Calpain-like cysteine peptidase, putative	Tc00,1047053508675,29	Q4D066	12,8	5,1	2	2	120
Cruzipain, putative	Tc00,1047053507603,270	Q4DW02	49,9	5,7	3	2	95,4
Cysteine peptidase C , putative	Tc00,1047053511827,100	Q4DQB0	36,7	5,8	2	2	46,9
Proteasome regulatory ATPase subunit 1, putative	Tc00,1047053506857,90	Q4D9J1	48,5	7,1	1	1	45,5
Proteasome regulatory ATPase subunit 2, putative	Tc00,1047053511047,50	Q4D0B9	49	5,5	1	1	54,8
Proteasome subunit alpha type	Tc00,1047053504069,10	Q4DAW6	25	5,7	2	2	55,6
Proteasome subunit beta type	Tc00,1047053503781,70	Q4D8U7	34,9	5,6	2	2	46,6
Proteasome subunit beta type	Tc00,1047053508461,430	Q4E4R6	33,2	9,1	1	1	75,5
Cysteine peptidase, putative	Tc00,1047053509429,320	Q4E0J7	49,8	5,4	5	4	161,3
Serine carboxypeptidase , putative	Tc00,1047053508671,20	Q4CMQ4	59,5	6,8	2	12	694
Serine carboxypeptidase , putative	Tc00,1047053509695,210	Q4DTP7	51,5	5	1	13	754,4
Serine carboxypeptidase , putative	Tc00,1047053509695,230	Q4DTP6	26,6	5,1	1	5	172,1
Serine carboxypeptidase S28, putative	Tc00,1047053506425,10	Q4DM56	72,1	6,2	3	5	145,8
**Carbohydrate metabolism**							
Fructose-bisphosphate aldolase	Tc00,1047053510301,20	Q4D0Q0	40,8	8,8	4	5	153,2
Enolase, putative	Tc00,1047053504105,140	Q4DZ98	46,4	5,9	1	11	382,3
Glyceraldehyde 3-phosphate dehydrogenase, putative	Tc00,1047053511461,14	Q4D3Y9	14,7	11,4	6	1	108,2
Glyceraldehyde 3-phosphate dehydrogenase, putative	Tc00,1047053509065,60	Q4DHF0	39	9	2	2	74,2
Lysosomal alpha-mannosidase, putative	Tc00,1047053506195,120	Q4DXL4	111,2	6,5	2	3	224
Malate dehydrogenase	Tc00,1047053507883,109	Q4D4A0	31,5	7,6	2	1	152,1
Isocitrate dehydrogenase [NADP]	Tc00,1047053506925,319	Q4E4L7	46,8	7,7	1	2	181
**Oxydo-Reduction**							
NAD/FAD dependent dehydrogenase, putative	Tc00,1047053505843,40	Q4CVH0	43	5,3	1	1	152,1
Cytochrome c, putative	Tc00,1047053508959,4	Q4CV48	12,2	9,6	2	3	79,2
Cytochrome c, putative	Tc00,1047053506949,50	Q4D480	12,2	9,4	2	3	95,1
Glutamate dehydrogenase	Tc00,1047053508111,30	Q4D5C2	45	8	2	2	81,2
Glutathione peroxidase	Tc00,1047053503899,119	Q4DEJ5	19,7	9	5	3	73,6
Superoxide dismutase	Tc00,1047053511715,10	Q4D5A6	23,3	7,1	4	3	301,3
Superoxide dismutase	Tc00,1047053511019,90	Q4DI29	21,9	6,6	4	5	197,2
Tryparedoxin peroxidase, putative	Tc00,1047053507259,10	Q4CM56	22,4	6,3	4	6	279,6

Tryparedoxin peroxidase, putative	Tc00,1047053509499,14	Q4CX87	25,5	7,6	1	2	195,6
Malic enzyme	Tc00,1047053508647,280	Q4DV36	62,7	6,5	2	2	64,7
Tryparedoxin, putative	Tc00,1047053509997,30	Q4D1B8	16	5,2	1	5	155,9
**Trafficking and membrane fusion**							
Dynein light chain, putative	Tc00,1047053506925,104	Q4E4N7	10,4	6,3	1	1	151
ADP-ribosylation factor 1, putative	Tc00,1047053508415,40	Q4D7Y8	20,7	8,5	2	4	193,1
Transitional endoplasmic reticulum ATPase, putative	Tc00,1047053509733,170	Q4DWB5	86,1	5,4	1	8	725,8
Small GTP-binding protein Rab1, putative	Tc00,1047053503715,30	Q4CZR0	22,8	7,6	5	2	173,7
Rab7 GTP binding protein, putative	Tc00,1047053508461,270	Q4E4T4	23,9	5,8	1	2	165,9
**Protein and Amino Acid metabolism**							
Adenosylhomocysteinase	Tc00,1047053511229,50	Q4D455	48,4	6,2	2	3	81,9
Arginine kinase, putative	Tc00,1047053507241,30	Q4CWA5	40,2	6,3	2	11	415,2
Aspartate aminotransferase	Tc00,1047053503841,70	Q4D080	44,8	7,6	2	1	116,2
ATP synthase subunit beta	Tc00,1047053509233,180	Q4DTX7	55,7	5,3	1	2	69,1
Ubiquitin/ribosomal protein S27a, putative	Tc00,1047053510409,39	Q4D1T6	16,6	9,6	3	1	45,4
Ubiquitin-activating enzyme E1, putative	Tc00,1047053504427,250	Q4DYM1	114,3	5,6	2	1	57,8
Seryl-tRNA synthetase, putative	Tc00,1047053511163,10	Q4CW46	25,7	5,5	2	2	102,2
Ubiquitin-conjugating enzyme E2, putative	Tc00,1047053508137,30	Q4CTN0	17,5	7,9	2	2	41,1
**Cell Structure and Motility**							
Profilin	Tc00,1047053510911,10	Q4CZF5	16,1	5	2	2	36,5
Surface protein TolT	Tc00,1047053506617,20	Q4D0C6	33	8,7	4	2	83,8
Surface protein TolT	Tc00,1047053508767,10	Q4CM39	24,8	9,4	3	3	107,6
Surface protein TolT	Tc00,1047053508767,20	Q4CM38	33	8,5	3	2	83,9
Surface protein TolT, putative	Tc00,1047053504277,11	Q4CNL2	21,5	8,8	1	3	66,4
Cofilin/actin depolymerizing factor, putative	Tc00,1047053510145,20	Q4CVE9	15,7	5,7	1	3	84
Cofilin/actin depolymerizing factor, putative	Tc00,1047053508411,10	Q4D8D3	15,7	5,7	1	3	84,7
Actin, putative	Tc00,1047053510127,79	Q4D7A6	38,1	5,4	3	2	180,8
Alpha tubulin, putative	Tc00,1047053411235,9	Q4CLA1	49,8	4,9	1	31	1609,4
Beta tubulin, putative	Tc00,1047053506563,40	Q4DQP2	49,7	4,7	1	19	1204,4

Microtubule-associated protein, putative	Tc00,1047053507447,19	Q4CMT2	85,2	4,8	2	7	372,5
**Nucleic acids binding**							
S-adenosylmethionine synthase	Tc00,1047053506945,160	Q4CSC4	43,5	6	1	2	81,9
Arginyl-tRNA synthetase, putative	Tc00,1047053508355,320	Q4E397	78,8	6,1	1	2	65,2
**Lipid metabolism**							
Lipase, putative	Tc00,1047053510679,70	Q4CYC4	23,9	6	7	2	43,6
**Transporters**							
GTP-binding nuclear protein rtb2, putative	Tc00,1047053509455,80	Q4DIB9	24,4	6,9	2	1	59,2
**Other**							
Cysteine peptidase inhibitor	Tc00,1047053506801,80	Q4DH32	12	6,6	1	8	554,4
Cysteine peptidase inhibitor, putative	Tc00,1047053511907,200	Q4DY71	12,1	6,6	1	7	637,9
Lectin, putative	Tc00,1047053506177,20	Q4DHU0	66	5,2	1	2	71,8
