# Supplementary material for: Secretome analysis of Trypanosoma cruzi by proteomics studies
Source: PLoS One. 2017 Oct 3;12(10):e0185504. doi: 10.1371/journal.pone.0185504 (PMC5626432; doi:10.1371/journal.pone.0185504)
Supplement: S3 Table — For each protein, the number of matched proteins and peptides and the highest score for CL Brener (white box) and VD (gray box) strain are described. (PDF) [file pone.0185504.s003.pdf]

S3 Table

| Rang | Protein | Accession Number | | MW | pI | Matching | Matching | Highest |
|---|---|---|---|---|---|---|---|---|
| | | Gene ID | UniprotKB | [kDa] | | Proteins | Peptides | Scores |
| 1 | Trans-sialidase, putative | Tc00.1047053510907.10 | Q4CPF9 | 103,3 | 5 | 3 | 22 | 2206,2 |
| | | | | | | 3 | 19 | 1464,5 |
| 2 | Trans-sialidase, putative | Tc00.1047053511835.10 | Q4CPQ2 | 110,5 | 5 | 2 | 25 | 1684,8 |
| | | | | | | 2 | 20 | 1318,7 |
| 3 | Trans-sialidase, putative | Tc00.1047053509333.10 | Q4CPQ3 | 108,9 | 4,8 | 1 | 18 | 1144 |
| | | | | | | 1 | 21 | 790,6 |
| 4 | Trans-sialidase, putative | Tc00.1047053509533.10 | Q4CXJ1 | 80,8 | 5,1 | 1 | 10 | 821 |
| | | | | | | 1 | 13 | 944 |
| 5 | Trans-sialidase, putative | Tc00.1047053507839.40 | Q4CXS5 | 80,8 | 8,4 | 1 | 32 | 2000,6 |
| | | | | | | 1 | 44 | 3875,6 |
| 6 | Trans-sialidase, putative | Tc00.1047053506353.10 | Q4CQ38 | 82,7 | 6,5 | 2 | 9 | 486,7 |
| | | | | | | 1 | 9 | 417,7 |
| 7 | Trans-sialidase, putative | Tc00.1047053510093.20 | Q4CQ85 | 113,7 | 4,9 | 1 | 15 | 1128 |
| | | | | | | 2 | 14 | 1160,5 |
| 8 | Trans-sialidase, putative | Tc00.1047053508103.30 | Q4CQL8 | 92,3 | 6,7 | 4 | 14 | 905,3 |
| | | | | | | 5 | 7 | 451,7 |
| 9 | Trans-sialidase, putative | Tc00.1047053506397.10 | Q4CQP5 | 81 | 5,8 | 1 | 17 | 910,3 |
| | | | | | | 1 | 16 | 833,9 |
| 10 | Trans-sialidase, putative | Tc00.1047053472357.10 | Q4CQY9 | 88 | 7,5 | 10 | 15 | 1448 |
| | | | | | | 30 | 20 | 1078,2 |
| 11 | Trans-sialidase, putative | Tc00.1047053503993.10 | Q4CQZ7 | 76,6 | 8,7 | 2 | 17 | 1546 |
| | | | | | | 1 | 17 | 1398,1 |
| 12 | Trans-sialidase, putative | Tc00.1047053504183.20 | Q4CR00 | 88,3 | 5,4 | 3 | 15 | 1487,2 |
| | | | | | | 2 | 15 | 1179,2 |
| 13 | Trans-sialidase, putative | Tc00.1047053507879.10 | Q4CPT6 | 86,4 | 5,8 | 2 | 12 | 842,1 |
| | | | | | | 1 | 15 | 1201,7 |
| 14 | Trans-sialidase, putative | Tc00.1047053507411.10 | Q4CPU0 | 97,5 | 5,1 | 3 | 2 | 220,5 |
| | | | | | | 3 | 4 | 366,4 |
| 15 | Trans-sialidase, putative | Tc00.1047053398477.10 | Q4CPW8 | 85,6 | 8,2 | 2 | 32 | 2748,6 |
| | | | | | | 1 | 50 | 4196,9 |
| 16 | Trans-sialidase, putative | Tc00.1047053463279.20 | Q4CPX0 | 80,7 | 6,3 | 2 | 25 | 2070,1 |
| | | | | | | 2 | 30 | 2206,4 |
| 17 | Trans-sialidase, putative | Tc00.1047053504145.10 | Q4CP42 | 81 | 6,3 | 1 | 3 | 149,6 |
| | | | | | | 1 | 8 | 443,3 |
| 18 | Trans-sialidase, putative | Tc00.1047053510569.10 | Q4CPY4 | 49,7 | 5,5 | 1 | 18 | 2035,5 |
| | | | | | | 2 | 24 | 1821,7 |
| 19 | Trans-sialidase, putative | Tc00.1047053509411.10 | Q4CRJ5 | 89,4 | 5,4 | 3 | 17 | 1461,4 |
| | | | | | | 5 | 21 | 1213,4 |
| 20 | Trans-sialidase, putative | Tc00.1047053505231.20 | Q4CRR3 | 84,2 | 6,6 | 2 | 9 | 445,8 |
| | | | | | | 2 | 5 | 310,4 |
| 21 | Trans-sialidase, putative | Tc00.1047053503907.10 | Q4CRT3 | 99,6 | 4,9 | 2 | 37 | 3234,8 |

| | | | | | | | | |
|---|---|---|---|---|---|---|---|---|
| | | | | | | 2 | 42 | 2950,1 |
| 22 | Trans-sialidase, putative | Tc00.1047053511833.10 | Q4CRY9 | 112,5 | 4,8 | 1 | 20 | 2099,4 |
| | | | | | | 1 | 17 | 1199 |
| 23 | Trans-sialidase, putative | Tc00.1047053509185.30 | Q4CRZ2 | 85,9 | 6,9 | 2 | 13 | 450,3 |
| | | | | | | 2 | 11 | 541,9 |
| 24 | Trans-sialidase, putative | Tc00.1047053507759.10 | Q4CRZ9 | 113 | 4,8 | 2 | 25 | 2596,3 |
| | | | | | | 2 | 36 | 2276,4 |
| 25 | Trans-sialidase, putative | Tc00.1047053511669.10 | Q4CRD4 | 113 | 4,8 | 2 | 18 | 1532,1 |
| | | | | | | 1 | 20 | 1371,1 |
| 26 | Trans-sialidase, putative | Tc00.1047053504341.10 | Q4CSD6 | 99,9 | 5,8 | 10 | 8 | 925,6 |
| | | | | | | 4 | 18 | 1331,9 |
| 27 | Trans-sialidase, putative | Tc00.1047053509629.10 | Q4CSG0 | 101,3 | 4,8 | 2 | 51 | 4628,2 |
| | | | | | | 4 | 67 | 5848,3 |
| 28 | Trans-sialidase, putative | Tc00.1047053509513.10 | Q4CSG5 | 81,6 | 8,7 | 1 | 34 | 2870,4 |
| | | | | | | 1 | 25 | 1480,6 |
| 29 | Trans-sialidase, putative | Tc00.1047053507427.10 | Q4CSI1 | 88,8 | 5,3 | 1 | 34 | 3676,4 |
| | | | | | | 1 | 42 | 3389,4 |
| 30 | Trans-sialidase, putative | Tc00.1047053509281.20 | Q4CSS2 | 79,2 | 5,9 | 3 | 16 | 864,7 |
| | | | | | | 3 | 23 | 1523,6 |
| 31 | Trans-sialidase, putative | Tc00.1047053503601.10 | Q4CSS7 | 85,3 | 8,7 | 21 | 15 | 988,3 |
| | | | | | | 1 | 6 | 323,6 |
| 32 | Trans-sialidase, putative | Tc00.1047053506427.10 | Q4CSV1 | 106,5 | 4,8 | 2 | 49 | 5043,9 |
| | | | | | | 3 | 43 | 3465,8 |
| 33 | Trans-sialidase, putative | Tc00.1047053504479.10 | Q4CSZ4 | 93,4 | 5,3 | 6 | 15 | 1173,5 |
| | | | | | | 2 | 28 | 2016,6 |
| 34 | Trans-sialidase, putative | Tc00.1047053507125.10 | Q4CTA6 | 85,8 | 8,7 | 1 | 20 | 1073,1 |
| | | | | | | 1 | 25 | 2338 |
| 35 | Trans-sialidase, putative | Tc00.1047053506021.20 | Q4CTT0 | 85,7 | 5,9 | 2 | 14 | 1097,9 |
| | | | | | | 1 | 12 | 979 |
| 36 | Trans-sialidase, putative | Tc00.1047053509931.20 | Q4CTU4 | 113,5 | 4,8 | 2 | 17 | 1827,2 |
| | | | | | | 2 | 26 | 2318,9 |
| 37 | Trans-sialidase, putative | Tc00.1047053506841.20 | Q4CTX4 | 85,9 | 5,6 | 1 | 35 | 2877,9 |
| | | | | | | 1 | 27 | 1915,3 |
| 38 | Trans-sialidase, putative | Tc00.1047053506455.30 | Q4CUA7 | 81,9 | 6,9 | 4 | 18 | 1321 |
| | | | | | | 4 | 16 | 736 |
| 39 | Trans-sialidase, putative | Tc00.1047053509377.20 | Q4CUE8 | 85,7 | 5,2 | 1 | 20 | 1920,1 |
| | | | | | | 1 | 26 | 1925 |
| 40 | Trans-sialidase, putative | Tc00.1047053509387.10 | Q4CUT1 | 112,3 | 5,1 | 1 | 21 | 1361,9 |
| | | | | | | 1 | 22 | 1364,4 |
| 41 | Trans-sialidase, putative | Tc00.1047053510787.10 | Q4CVS5 | 124,8 | 5,8 | 4 | 38 | 3060,9 |
| | | | | | | 3 | 47 | 3644,7 |
| 42 | Trans-sialidase, putative | Tc00.1047053509187.10 | Q4CUU4 | 81,8 | 9,2 | 1 | 13 | 783,5 |
| | | | | | | 2 | 20 | 853,3 |
| 43 | Trans-sialidase, putative | Tc00.1047053510331.10 | Q4CUZ0 | 106,2 | 5 | 1 | 29 | 2152,1 |
| | | | | | | 1 | 43 | 2521,5 |

| | | | | | | | | |
|---|---|---|---|---|---|---|---|---|
| 44 | Trans-sialidase, putative | Tc00.1047053506217.40 | Q4CVZ4 | 113,6 | 5 | 3 | 28 | 3181,3 |
| | | | | | | 2 | 28 | 1825,1 |
| 45 | Trans-sialidase, putative | Tc00.1047053507313.20 | Q4CW78 | 112,8 | 5 | 1 | 22 | 1618,6 |
| | | | | | | 1 | 22 | 1559 |
| 46 | Trans-sialidase, putative | Tc00.1047053508455.20 | Q4CWC5 | 95,1 | 5,1 | 4 | 5 | 304,3 |
| | | | | | | 2 | 5 | 299,6 |
| 47 | Trans-sialidase, putative | Tc00.1047053507473.30 | Q4CWN6 | 81,3 | 5,2 | 2 | 19 | 1531,8 |
| | | | | | | 2 | 15 | 901,9 |
| 48 | Trans-sialidase, putative | Tc00.1047053504193.15 | Q4CWT8 | 29,6 | 9,6 | 1 | 3 | 240,8 |
| | | | | | | 1 | 2 | 171,3 |
| 49 | Trans-sialidase, putative | Tc00.1047053504101.50 | Q4CX14 | 82,5 | 8,7 | 1 | 4 | 387,3 |
| | | | | | | 27 | 9 | 505,1 |
| 50 | Trans-sialidase, putative | Tc00.1047053511779.60 | Q4CXY5 | 101,1 | 4,9 | 1 | 28 | 2198 |
| | | | | | | 1 | 42 | 3066,5 |
| 51 | Trans-sialidase, putative | Tc00.1047053511551.10 | Q4CYA8 | 85,9 | 5,2 | 4 | 26 | 3013,4 |
| | | | | | | 6 | 28 | 2736 |
| 52 | Trans-sialidase, putative | Tc00.1047053509843.20 | Q4CYS5 | 95 | 5,7 | 1 | 34 | 3805,2 |
| | | | | | | 1 | 40 | 3050 |
| 53 | Trans-sialidase, putative | Tc00.1047053506341.50 | Q4CYW2 | 112,2 | 4,8 | 2 | 21 | 1620,3 |
| | | | | | | 1 | 28 | 1623 |
| 54 | Trans-sialidase, putative | Tc00.1047053509495.30 | Q4CZ79 | 99,4 | 6 | 7 | 39 | 3286,1 |
| | | | | | | 5 | 49 | 4088 |
| 55 | Trans-sialidase, putative | Tc00.1047053507753.10 | Q4CZ80 | 113,8 | 5,1 | 1 | 19 | 1668,1 |
| | | | | | | 1 | 30 | 1483 |
| 56 | Trans-sialidase, putative | Tc00.1047053511057.40 | Q4CZ90 | 84 | 5,3 | 4 | 9 | 390,5 |
| | | | | | | 2 | 10 | 560,5 |
| 57 | Trans-sialidase, putative | Tc00.1047053508581.40 | Q4CZ95 | 75,4 | 6,8 | 2 | 21 | 1902,2 |
| | | | | | | 2 | 29 | 1921,8 |
| 58 | Trans-sialidase, putative | Tc00.1047053508581.10 | Q4CZ96 | 90,7 | 5,7 | 1 | 10 | 614 |
| | | | | | | 1 | 11 | 499,2 |
| 59 | Trans-sialidase, putative | Tc00.1047053506723.20 | Q4CZC3 | 93,3 | 5,8 | 1 | 25 | 2361,6 |
| | | | | | | 2 | 24 | 1589,4 |
| 60 | Trans-sialidase, putative | Tc00.1047053507979.30 | Q4CZE6 | 89,4 | 8,4 | 3 | 16 | 985,1 |
| | | | | | | 3 | 15 | 925,9 |
| 61 | Trans-sialidase, putative | Tc00.1047053509685.40 | Q4D095 | 93,7 | 5,3 | 5 | 6 | 428,3 |
| | | | | | | 6 | 6 | 463,7 |
| 62 | Trans-sialidase, putative | Tc00.1047053505993.30 | Q4D0D9 | 111,9 | 4,8 | 4 | 26 | 2279,4 |
| | | | | | | 1 | 26 | 1786,6 |
| 63 | Trans-sialidase, putative | Tc00.1047053510125.20 | Q4D0T2 | 113,6 | 4,8 | 1 | 23 | 2143,9 |
| | | | | | | 2 | 27 | 1424 |
| 64 | Trans-sialidase, putative | Tc00.1047053507121.20 | Q4D110 | 89,1 | 5,4 | 2 | 35 | 2201,1 |
| | | | | | | 1 | 41 | 2670,9 |
| 65 | Trans-sialidase, putative | Tc00.1047053508627.40 | Q4D1A3 | 113,8 | 4,9 | 3 | 48 | 4719,9 |
| | | | | | | 2 | 52 | 4477,9 |
| 66 | Trans-sialidase, putative | Tc00.1047053510491.60 | Q4D1A6 | 93,9 | 5,5 | 1 | 4 | 234,2 |

| | | | | | | | | |
|---|---|---|---|---|---|---|---|---|
| | | | | | | 1 | 5 | 374,3 |
| 67 | Trans-sialidase, putative | Tc00.1047053508055.30 | Q4D1Z3 | 85,2 | 5,9 | 9 | 7 | 855,2 |
| | | | | | | 2 | 29 | 2534,7 |
| 68 | Trans-sialidase, putative | Tc00.1047053510635.10 | Q4D232 | 91 | 5,4 | 2 | 45 | 5240,6 |
| | | | | | | 2 | 36 | 3237,3 |
| 69 | Trans-sialidase, putative | Tc00.1047053511117.30 | Q4D293 | 81,8 | 6,3 | 2 | 12 | 948,8 |
| | | | | | | 2 | 17 | 1492,7 |
| 70 | Trans-sialidase, putative | Tc00.1047053505609.40 | Q4D2F9 | 83,3 | 7,2 | 8 | 11 | 797,4 |
| | | | | | | 11 | 12 | 423,3 |
| 71 | Trans-sialidase, putative | Tc00.1047053507255.20 | Q4D2I1 | 85,3 | 6,2 | 1 | 28 | 2332,3 |
| | | | | | | 1 | 33 | 2985,7 |
| 72 | Trans-sialidase, putative | Tc00.1047053510411.10 | Q4D2I7 | 91,9 | 5,2 | 3 | 22 | 1957,2 |
| | | | | | | 1 | 18 | 1178,8 |
| 73 | Trans-sialidase, putative | Tc00.1047053510979.40 | Q4D2L1 | 91,8 | 5,9 | 2 | 23 | 1065,1 |
| | | | | | | 5 | 13 | 688,2 |
| 74 | Trans-sialidase, putative | Tc00.1047053505207.10 | Q4D308 | 88,6 | 5,6 | 1 | 17 | 1444,1 |
| | | | | | | 1 | 19 | 919,9 |
| 75 | Trans-sialidase, putative | Tc00.1047053506603.40 | Q4D349 | 82,4 | 8,5 | 8 | 17 | 1169,8 |
| | | | | | | 4 | 8 | 581,3 |
| 76 | Trans-sialidase, putative | Tc00.1047053506597.40 | Q4D371 | 82,8 | 8,5 | 5 | 14 | 1044,6 |
| | | | | | | 6 | 13 | 555,1 |
| 77 | Trans-sialidase, putative | Tc00.1047053505975.20 | Q4D3K3 | 152,1 | 5,6 | 9 | 12 | 520,6 |
| | | | | | | 8 | 27 | 2391,9 |
| 78 | Trans-sialidase, putative | Tc00.1047053510205.40 | Q4D3Q8 | 83,9 | 5,5 | 1 | 6 | 343,6 |
| | | | | | | 1 | 9 | 437,6 |
| 79 | Trans-sialidase, putative | Tc00.1047053508451.60 | Q4D4B5 | 85,8 | 5,3 | 3 | 15 | 1416,3 |
| | | | | | | 4 | 18 | 1246,5 |
| 80 | Trans-sialidase, putative | Tc00.1047053506171.60 | Q4D5B9 | 74 | 5,4 | 1 | 9 | 991,4 |
| | | | | | | 1 | 19 | 841,6 |
| 81 | Trans-sialidase, putative | Tc00.1047053511219.40 | Q4D5K5 | 86,7 | 5,7 | 1 | 17 | 630,1 |
| | | | | | | 1 | 23 | 1822,5 |
| 82 | Trans-sialidase, putative | Tc00.1047053507907.20 | Q4D625 | 79 | 6,2 | 1 | 17 | 1375,5 |
| | | | | | | 1 | 12 | 670,4 |
| 83 | Trans-sialidase, putative | Tc00.1047053509755.10 | Q4D649 | 82,5 | 5,7 | 3 | 8 | 364,7 |
| | | | | | | 3 | 9 | 447,4 |
| 84 | Trans-sialidase, putative | Tc00.1047053507819.30 | Q4D6J3 | 85,1 | 5,6 | 1 | 19 | 928,9 |
| | | | | | | 1 | 27 | 2305,9 |
| 85 | Trans-sialidase, putative | Tc00.1047053511885.20 | Q4D6K2 | 84,6 | 5,6 | 2 | 15 | 1253 |
| | | | | | | 2 | 16 | 1064,9 |
| 86 | Trans-sialidase, putative | Tc00.1047053509427.10 | Q4D6S1 | 79,4 | 5,9 | 2 | 9 | 601,7 |
| | | | | | | 1 | 8 | 362,9 |
| 87 | Trans-sialidase, putative | Tc00.1047053506053.50 | Q4D6Y6 | 98,9 | 6,1 | 1 | 4 | 176,9 |
| | | | | | | 2 | 10 | 737,4 |
| 88 | Trans-sialidase, putative | Tc00.1047053503447.20 | Q4D7N9 | 82,7 | 8,9 | 1 | 10 | 515,6 |
| | | | | | | 1 | 11 | 559,8 |

| | | | | | | | | |
|---|---|---|---|---|---|---|---|---|
| 89 | Trans-sialidase, putative | Tc00.1047053508285.60 | Q4D825 | 85,1 | 5,4 | 4 | 24 | 1094,3 |
| | | | | | | 3 | 32 | 2100,6 |
| 90 | Trans-sialidase, putative | Tc00.1047053505919.20 | Q4D8B3 | 78,6 | 8,8 | 1 | 9 | 951,9 |
| | | | | | | 1 | 9 | 940,5 |
| 91 | Trans-sialidase, putative | Tc00.1047053511129.40 | Q4D8H5 | 113,3 | 4,9 | 1 | 37 | 3777,9 |
| | | | | | | 1 | 45 | 3347,5 |
| 92 | Trans-sialidase, putative | Tc00.1047053509075.50 | Q4D8V4 | 80,2 | 5,6 | 1 | 16 | 1104,8 |
| | | | | | | 1 | 19 | 1162,5 |
| 93 | Trans-sialidase, putative | Tc00.1047053509529.40 | Q4DDZ1 | 94,2 | 5,3 | 1 | 25 | 2374,1 |
| | | | | | | 1 | 31 | 2188,3 |
| 94 | Trans-sialidase, putative | Tc00.1047053508521.100 | Q4DE10 | 119,9 | 4,9 | 1 | 19 | 1530,8 |
| | | | | | | 1 | 20 | 1213,3 |
| 95 | Trans-sialidase, putative | Tc00.1047053508521.20 | Q4DE13 | 112,4 | 4,9 | 2 | 22 | 1693,1 |
| | | | | | | 1 | 26 | 1793,2 |
| 96 | Trans-sialidase, putative | Tc00.1047053509663.50 | Q4DE15 | 112,4 | 4,7 | 1 | 18 | 1297,5 |
| | | | | | | 1 | 25 | 1228,4 |
| 97 | Trans-sialidase, putative | Tc00.1047053505365.60 | Q4DEB7 | 113,3 | 5 | 1 | 34 | 1905,6 |
| | | | | | | 1 | 42 | 2981,1 |
| 98 | Trans-sialidase, putative | Tc00.1047053509785.50 | Q4DEK8 | 87,5 | 6 | 7 | 10 | 696,9 |
| | | | | | | 5 | 10 | 737,2 |
| 99 | Trans-sialidase, putative | Tc00.1047053507233.10 | Q4DEP9 | 112,3 | 5 | 1 | 22 | 1767,8 |
| | | | | | | 1 | 25 | 1256,8 |
| 100 | Trans-sialidase, putative | Tc00.1047053510847.10 | Q4DF05 | 108,5 | 4,7 | 1 | 31 | 2222,5 |
| | | | | | | 1 | 32 | 2095,2 |
| 101 | Trans-sialidase, putative | Tc00.1047053511587.90 | Q4DFH1 | 112,9 | 4,8 | 2 | 30 | 2417,2 |
| | | | | | | 1 | 34 | 2626,9 |
| 102 | Trans-sialidase, putative | Tc00.1047053504425.10 | Q4DFP2 | 108 | 4,8 | 2 | 26 | 2102 |
| | | | | | | 2 | 39 | 1876,5 |
| 103 | Trans-sialidase, putative | Tc00.1047053506129.50 | Q4DFT5 | 159,2 | 6,6 | 1 | 8 | 498,8 |
| | | | | | | 3 | 19 | 1495,9 |
| 104 | Trans-sialidase, putative | Tc00.1047053510403.30 | Q4D980 | 82,2 | 6,1 | 1 | 11 | 589,4 |
| | | | | | | 3 | 12 | 510,6 |
| 105 | Trans-sialidase, putative | Tc00.1047053508717.60 | Q4D9H3 | 86,1 | 5,8 | 1 | 35 | 3110,9 |
| | | | | | | 1 | 28 | 1743,2 |
| 106 | Trans-sialidase, putative | Tc00.1047053511875.20 | Q4DA40 | 99 | 5,1 | 1 | 39 | 3491,6 |
| | | | | | | 1 | 36 | 2943,6 |
| 107 | Trans-sialidase, putative | Tc00.1047053504099.50 | Q4DBC9 | 78,1 | 5,5 | 1 | 7 | 526,1 |
| | | | | | | 1 | 7 | 491,3 |
| 108 | Trans-sialidase, putative | Tc00.1047053511757.70 | Q4DBD3 | 78,9 | 6,2 | 1 | 12 | 529,2 |
| | | | | | | 1 | 12 | 543,9 |
| 109 | Trans-sialidase, putative | Tc00.1047053509765.50 | Q4DBF1 | 112,9 | 4,9 | 1 | 23 | 1490,5 |
| | | | | | | 1 | 26 | 1859,8 |
| 110 | Trans-sialidase, putative | Tc00.1047053506345.90 | Q4DCE1 | 91,6 | 5 | 4 | 24 | 1890,7 |
| | | | | | | 1 | 18 | 1135,5 |
| 111 | Trans-sialidase, putative | Tc00.1047053507479.20 | Q4DCG7 | 89 | 5,5 | 2 | 28 | 2368,6 |

| | | | | | | | | |
|---|---|---|---|---|---|---|---|---|
| | | | | | | 2 | 43 | 3865 |
| 112 | Trans-sialidase, putative | Tc00.1047053510095.20 | Q4DGR6 | 83,4 | 6,5 | 1 | 6 | 476,7 |
| | | | | | | 1 | 5 | 181,6 |
| 113 | Trans-sialidase, putative | Tc00.1047053509265.120 | Q4DGT1 | 80,5 | 5,5 | 7 | 11 | 1131,9 |
| | | | | | | 1 | 17 | 1263,6 |
| 114 | Trans-sialidase, putative | Tc00.1047053509265.110 | Q4DGT2 | 185,2 | 6,4 | 4 | 6 | 653,5 |
| | | | | | | 2 | 19 | 1357,3 |
| 115 | Trans-sialidase, putative | Tc00.1047053509265.90 | Q4DGT3 | 86,2 | 6,1 | 3 | 42 | 3882 |
| | | | | | | 1 | 39 | 3436,3 |
| 116 | Trans-sialidase, putative | Tc00.1047053506975.90 | Q4DGV8 | 112,5 | 4,9 | 1 | 42 | 4503,5 |
| | | | | | | 1 | 34 | 2812,5 |
| 117 | Trans-sialidase, putative | Tc00.1047053506975.80 | Q4DGV9 | 78 | 5,1 | 1 | 16 | 1179,5 |
| | | | | | | 3 | 24 | 1880,2 |
| 118 | Trans-sialidase, putative | Tc00.1047053509905.170 | Q4DR51 | 113,1 | 5 | 1 | 19 | 1615 |
| | | | | | | 1 | 29 | 1322,1 |
| 119 | Trans-sialidase, putative | Tc00.1047053506577.80 | Q4DR84 | 99,1 | 5 | 1 | 18 | 1701,6 |
| | | | | | | 1 | 16 | 1371,3 |
| 120 | Trans-sialidase, putative | Tc00.1047053507653.20 | Q4DI36 | 85,8 | 5,5 | 1 | 13 | 668,9 |
| | | | | | | 1 | 11 | 438,7 |
| 121 | Trans-sialidase, putative | Tc00.1047053506965.170 | Q4DIE5 | 81,2 | 5,4 | 1 | 11 | 454,3 |
| | | | | | | 10 | 17 | 1122,6 |
| 122 | Trans-sialidase, putative | Tc00.1047053508045.120 | Q4DIN5 | 108,8 | 4,7 | 1 | 17 | 1419,7 |
| | | | | | | 1 | 22 | 916,1 |
| 123 | Trans-sialidase, putative | Tc00.1047053507357.100 | Q4DCY7 | 85,7 | 8,6 | 3 | 21 | 1828 |
| | | | | | | 1 | 21 | 1115,5 |
| 124 | Trans-sialidase, putative | Tc00.1047053508061.20 | Q4DKB6 | 89,8 | 5,4 | 1 | 14 | 1338,7 |
| | | | | | | 1 | 17 | 1072,5 |
| 125 | Trans-sialidase, putative | Tc00.1047053508607.50 | Q4DKL5 | 93,2 | 6,7 | 2 | 13 | 930,9 |
| | | | | | | 2 | 15 | 1136,7 |
| 126 | Trans-sialidase, putative | Tc00.1047053506471.120 | Q4DKM3 | 101,7 | 5 | 8 | 31 | 2823,7 |
| | | | | | | 4 | 32 | 2766,1 |
| 127 | Trans-sialidase, putative | Tc00.1047053508523.30 | Q4DL67 | 87,4 | 5,5 | 1 | 8 | 365,9 |
| | | | | | | 1 | 9 | 500,2 |
| 128 | Trans-sialidase, putative | Tc00.1047053509549.10 | Q4DLR5 | 85,4 | 6,6 | 6 | 22 | 2191,1 |
| | | | | | | 7 | 23 | 1451,4 |
| 129 | Trans-sialidase, putative | Tc00.1047053511861.50 | Q4DMZ9 | 114,2 | 4,7 | 1 | 13 | 1223,5 |
| | | | | | | 1 | 13 | 968,2 |
| 130 | Trans-sialidase, putative | Tc00.1047053507085.30 | Q4DQ76 | 94,9 | 6,3 | 1 | 37 | 2918,6 |
| | | | | | | 1 | 45 | 3544,8 |
| 131 | Trans-sialidase, putative | Tc00.1047053506757.120 | Q4DQA0 | 87,5 | 5,6 | 25 | 27 | 2410,5 |
| | | | | | | 16 | 31 | 1746,7 |
| 132 | Trans-sialidase, putative | Tc00.1047053506757.60 | Q4DQA2 | 87,2 | 5,8 | 9 | 30 | 2537,1 |
| | | | | | | 15 | 36 | 2282,4 |
| 133 | Trans-sialidase, putative | Tc00.1047053511827.110 | Q4DQA9 | 78,4 | 6,1 | 1 | 5 | 281,6 |
| | | | | | | 1 | 2 | 52,7 |

| | | | | | | | | |
|---|---|---|---|---|---|---|---|---|
| 134 | Trans-sialidase, putative | Tc00.1047053508871.10 | Q4DQS8 | 83,5 | 8,1 | 29 | 10 | 792,2 |
| | | | | | | 49 | 7 | 360,3 |
| 135 | Trans-sialidase, putative | Tc00.1047053511911.60 | Q4DQV8 | 113,6 | 4,9 | 1 | 37 | 3942,8 |
| | | | | | | 1 | 47 | 2827,2 |
| 136 | Trans-sialidase, putative | Tc00.1047053511911.10 | Q4DQW0 | 80,3 | 6,3 | 1 | 10 | 467,5 |
| | | | | | | 1 | 15 | 770,5 |
| 137 | Trans-sialidase, putative | Tc00.1047053509905.200 | Q4DR49 | 82 | 5,5 | 2 | 20 | 2314,7 |
| | | | | | | 2 | 17 | 1396,2 |
| 138 | Trans-sialidase, putative | Tc00.1047053507949.210 | Q4DS03 | 81,9 | 8,2 | 1 | 16 | 1237,4 |
| | | | | | | 1 | 18 | 1203,6 |
| 139 | Trans-sialidase, putative | Tc00.1047053511185.80 | Q4DPD2 | 91,7 | 4,9 | 1 | 5 | 369,3 |
| | | | | | | 1 | 3 | 226 |
| 140 | Trans-sialidase, putative | Tc00.1047053507047.40 | Q4DPT3 | 82 | 8,6 | 8 | 21 | 1607,4 |
| | | | | | | 6 | 31 | 1704,9 |
| 141 | Trans-sialidase, putative | Tc00.1047053503861.40 | Q4DSI1 | 112,8 | 4,8 | 1 | 18 | 1141,8 |
| | | | | | | 2 | 25 | 1729,1 |
| 142 | Trans-sialidase, putative | Tc00.1047053510025.50 | Q4DTC3 | 93,5 | 8,2 | 4 | 8 | 555,9 |
| | | | | | | 2 | 7 | 588,9 |
| 143 | Trans-sialidase, putative | Tc00.1047053509157.170 | Q4DTD3 | 107,3 | 4,9 | 2 | 46 | 4447,4 |
| | | | | | | 1 | 46 | 3730,4 |
| 144 | Trans-sialidase, putative | Tc00.1047053506499.170 | Q4DU07 | 82,9 | 8,8 | 2 | 15 | 963,8 |
| | | | | | | 4 | 13 | 728,1 |
| 145 | Trans-sialidase, putative | Tc00.1047053506241.30 | Q4DU42 | 82,3 | 6,2 | 3 | 9 | 423,8 |
| | | | | | | 2 | 8 | 396,1 |
| 146 | Trans-sialidase, putative | Tc00.1047053510713.30 | Q4DUP3 | 90,3 | 5,5 | 3 | 33 | 2946,4 |
| | | | | | | 3 | 34 | 2832,8 |
| 147 | Trans-sialidase, putative | Tc00.1047053510021.120 | Q4DUY7 | 86,9 | 5,7 | 1 | 20 | 1360,3 |
| | | | | | | 1 | 25 | 1110,8 |
| 148 | Trans-sialidase, putative | Tc00.1047053506331.130 | Q4DV16 | 80,9 | 6,4 | 1 | 29 | 3262,8 |
| | | | | | | 1 | 33 | 2804,2 |
| 149 | Trans-sialidase, putative | Tc00.1047053506331.90 | Q4DV17 | 83,2 | 5,5 | 1 | 12 | 1064,5 |
| | | | | | | 1 | 13 | 709,8 |
| 150 | Trans-sialidase, putative | Tc00.1047053506409.170 | Q4DV25 | 98,7 | 4,9 | 1 | 7 | 449,3 |
| | | | | | | 1 | 5 | 203,4 |
| 151 | Trans-sialidase, putative | Tc00.1047053506961.150 | Q4DVI7 | 114,1 | 4,7 | 1 | 32 | 2687,5 |
| | | | | | | 1 | 35 | 2536 |
| 152 | Trans-sialidase, putative | Tc00.1047053506961.25 | Q4DVJ1 | 107,8 | 4,7 | 1 | 30 | 3626,7 |
| | | | | | | 1 | 30 | 2577,9 |
| 153 | Trans-sialidase, putative | Tc00.1047053511183.430 | Q4E5K1 | 91,9 | 4,8 | 2 | 5 | 421,1 |
| | | | | | | 2 | 5 | 400,6 |
| 154 | Trans-sialidase, putative | Tc00.1047053505997.80 | Q4DWA9 | 111,4 | 4,9 | 1 | 15 | 933,7 |
| | | | | | | 2 | 31 | 1842,4 |
| 155 | Trans-sialidase, putative | Tc00.1047053510643.40 | Q4DWU9 | 113 | 4,9 | 1 | 26 | 2370,5 |
| | | | | | | 2 | 24 | 1932,1 |
| 156 | Trans-sialidase, putative | Tc00.1047053507875.220 | Q4DWV1 | 86,1 | 5,3 | 1 | 15 | 632 |

| | | | | | | | | |
|---|---|---|---|---|---|---|---|---|
| | | | | | | 3 | 21 | 1600,9 |
| 157 | Trans-sialidase, putative | Tc00.1047053507875.70 | Q4DWV4 | 94,4 | 5,3 | 1 | 25 | 2391,4 |
| | | | | | | 1 | 29 | 2487,8 |
| 158 | Trans-sialidase, putative | Tc00.1047053506717.80 | Q4DX60 | 113,7 | 5,1 | 1 | 34 | 2411,4 |
| | | | | | | 1 | 42 | 2711,2 |
| 159 | Trans-sialidase, putative | Tc00.1047053506813.140 | Q4DY88 | 89,5 | 5,5 | 1 | 23 | 2328,4 |
| | | | | | | 1 | 25 | 2179,7 |
| 160 | Trans-sialidase, putative | Tc00.1047053506683.240 | Q4DYC7 | 80,9 | 6,2 | 1 | 8 | 540,4 |
| | | | | | | 1 | 12 | 854,7 |
| 161 | Trans-sialidase, putative | Tc00.1047053506683.110 | Q4DYD0 | 113,7 | 4,8 | 2 | 36 | 2891,5 |
| | | | | | | 1 | 46 | 2858 |
| 162 | Trans-sialidase, putative | Tc00.1047053504427.230 | Q4DYM3 | 108,6 | 4,8 | 1 | 52 | 4973,7 |
| | | | | | | 1 | 50 | 3258,5 |
| 163 | Trans-sialidase, putative | Tc00.1047053507237.10 | Q4DYY9 | 81,5 | 6,1 | 2 | 10 | 321,1 |
| | | | | | | 1 | 17 | 863 |
| 164 | Trans-sialidase, putative | Tc00.1047053506763.110 | Q4DZC4 | 114,9 | 4,8 | 4 | 21 | 1101,5 |
| | | | | | | 7 | 25 | 1027,6 |
| 165 | Trans-sialidase, putative | Tc00.1047053506537.200 | Q4DZD4 | 120,1 | 5,2 | 1 | 42 | 4240,4 |
| | | | | | | 1 | 45 | 4291,2 |
| 166 | Trans-sialidase, putative | Tc00.1047053506537.80 | Q4DZD8 | 85,6 | 5,5 | 2 | 27 | 2313,8 |
| | | | | | | 4 | 25 | 1672,3 |
| 167 | Trans-sialidase, putative | Tc00.1047053509875.80 | Q4E0D0 | 81,7 | 8,9 | 3 | 16 | 1135,5 |
| | | | | | | 3 | 14 | 971,9 |
| 168 | Trans-sialidase, putative | Tc00.1047053508325.230 | Q4E0H9 | 161,1 | 8,2 | 3 | 13 | 669,8 |
| | | | | | | 2 | 28 | 2604,3 |
| 169 | Trans-sialidase, putative | Tc00.1047053510307.240 | Q4E0M8 | 113,5 | 4,9 | 1 | 31 | 2847 |
| | | | | | | 1 | 36 | 2203,2 |
| 170 | Trans-sialidase, putative | Tc00.1047053508139.240 | Q4E1E4 | 83,9 | 6,4 | 2 | 47 | 5304 |
| | | | | | | 1 | 44 | 4172,4 |
| 171 | Trans-sialidase, putative | Tc00.1047053510483.360 | Q4E1F4 | 92,1 | 5,4 | 2 | 30 | 2641,8 |
| | | | | | | 1 | 26 | 1328,3 |
| 172 | Trans-sialidase, putative | Tc00.1047053511603.450 | Q4E2A1 | 83,2 | 8,1 | 2 | 6 | 721,8 |
| | | | | | | 3 | 11 | 464,4 |
| 173 | Trans-sialidase, putative | Tc00.1047053511603.90 | Q4E2C0 | 97,2 | 5,4 | 1 | 11 | 517,9 |
| | | | | | | 14 | 10 | 586 |
| 174 | Trans-sialidase, putative | Tc00.1047053509979.320 | Q4E2C9 | 82,8 | 8,5 | 2 | 11 | 707,3 |
| | | | | | | 5 | 9 | 518,7 |
| 175 | Trans-sialidase, putative | Tc00.1047053510279.320 | Q4E373 | 98,5 | 5,4 | 6 | 7 | 488,9 |
| | | | | | | 2 | 5 | 266,7 |
| 176 | Trans-sialidase, putative | Tc00.1047053508355.80 | Q4E3C3 | 101,8 | 4,9 | 1 | 22 | 1864,4 |
| | | | | | | 1 | 19 | 1588,4 |
| 177 | Trans-sialidase, putative | Tc00.1047053507611.170 | Q4E3G9 | 85,4 | 6,1 | 1 | 19 | 1848,7 |
| | | | | | | 1 | 18 | 945 |
| 178 | Trans-sialidase, putative | Tc00.1047053510377.330 | Q4E3V0 | 86,9 | 5,4 | 8 | 18 | 1207,3 |
| | | | | | | 9 | 23 | 1060,5 |

| | | | | | | | | |
|---|---|---|---|---|---|---|---|---|
| 179 | Trans-sialidase, putative | Tc00.1047053510377.10 | Q4E3W2 | 83,3 | 8,6 | 4 | 15 | 1123,5 |
| | | | | | | 8 | 14 | 743,5 |
| 180 | Trans-sialidase, putative | Tc00.1047053511173.470 | Q4E424 | 98,7 | 5 | 57 | 11 | 1037,5 |
| | | | | | | 31 | 6 | 191,5 |
| 181 | Trans-sialidase, putative | Tc00.1047053511173.440 | Q4E426 | 82,5 | 5,4 | 1 | 10 | 291,8 |
| | | | | | | 43 | 15 | 719,2 |
| 182 | Trans-sialidase, putative | Tc00.1047053511173.280 | Q4E433 | 96,8 | 5,1 | 3 | 4 | 418,4 |
| | | | | | | 3 | 6 | 413,8 |
| 183 | Trans-sialidase, putative | Tc00.1047053511173.130 | Q4E440 | 98,9 | 5,1 | 32 | 14 | 1148,8 |
| | | | | | | 3 | 12 | 730,5 |
| 184 | Trans-sialidase, putative | Tc00.1047053506885.210 | Q4E4H9 | 106,2 | 5 | 2 | 30 | 2497,9 |
| | | | | | | 3 | 30 | 1302,4 |
| 185 | Trans-sialidase, putative (Fragment) | Tc00.1047053505155.4 | Q4CPN2 | 27,8 | 8,7 | 2 | 6 | 685,6 |
| | | | | | | 2 | 7 | 855,5 |
| 186 | Trans-sialidase, putative (Fragment) | Tc00.1047053504593.10 | Q4CPN3 | 80,5 | 4,9 | 1 | 18 | 1388,1 |
| | | | | | | 1 | 28 | 1565,3 |
| 187 | Trans-sialidase, putative (Fragment) | Tc00.1047053426675.9 | Q4CKC3 | 29,9 | 7,1 | 2 | 10 | 578,2 |
| | | | | | | 2 | 8 | 455,1 |
| 188 | Trans-sialidase, putative (Fragment) | Tc00.1047053423707.10 | Q4CKC5 | 33,2 | 6,6 | 2 | 4 | 230,6 |
| | | | | | | 3 | 5 | 514,2 |
| 189 | Trans-sialidase, putative (Fragment) | Tc00.1047053455171.9 | Q4CKG0 | 44,5 | 5,9 | 2 | 12 | 759,2 |
| | | | | | | 1 | 4 | 248,8 |
| 190 | Trans-sialidase, putative (Fragment) | Tc00.1047053433733.10 | Q4CKI9 | 37,8 | 5,5 | 1 | 13 | 1515,3 |
| | | | | | | 1 | 14 | 1142,5 |
| 191 | Trans-sialidase, putative (Fragment) | Tc00.1047053432995.9 | Q4CKL4 | 48,3 | 5,9 | 1 | 6 | 407,8 |
| | | | | | | 1 | 8 | 234,1 |
| 192 | Trans-sialidase, putative (Fragment) | Tc00.1047053423205.10 | Q4CL03 | 15 | 8,5 | 1 | 2 | 269,4 |
| | | | | | | 1 | 2 | 174,4 |
| 193 | Trans-sialidase, putative (Fragment) | Tc00.1047053401569.10 | Q4CL29 | 38,5 | 5,3 | 2 | 9 | 940,7 |
| | | | | | | 2 | 11 | 1104,8 |
| 194 | Trans-sialidase, putative (Fragment) | Tc00.1047053508639.10 | Q4CL30 | 28,9 | 4,5 | 1 | 30 | 2032,7 |
| | | | | | | 1 | 30 | 2447,6 |
| 195 | Trans-sialidase, putative (Fragment) | Tc00.1047053506003.39 | Q4CQC9 | 66,5 | 7,6 | 1 | 7 | 475,2 |
| | | | | | | 1 | 11 | 758,9 |
| 196 | Trans-sialidase, putative (Fragment) | Tc00.1047053432997.10 | Q4CLF9 | 67,2 | 5,9 | 1 | 34 | 3033,9 |
| | | | | | | 1 | 37 | 2450,6 |
| 197 | Trans-sialidase, putative (Fragment) | Tc00.1047053459061.10 | Q4CLG7 | 28,1 | 9,3 | 2 | 14 | 1189,3 |
| | | | | | | 2 | 10 | 729,3 |
| 198 | Trans-sialidase, putative (Fragment) | Tc00.1047053508849.11 | Q4CLZ0 | 14,6 | 11 | 1 | 1 | 153,1 |
| | | | | | | 1 | 1 | 75,6 |
| 199 | Trans-sialidase, putative (Fragment) | Tc00.1047053510049.10 | Q4CN26 | 67,6 | 6,1 | 1 | 15 | 1206,4 |
| | | | | | | 1 | 19 | 1415,1 |
| 200 | Trans-sialidase, putative (Fragment) | Tc00.1047053510881.9 | Q4CN34 | 78,7 | 6,1 | 2 | 23 | 1998,9 |
| | | | | | | 1 | 30 | 2242,1 |
| 201 | Trans-sialidase, putative (Fragment) | Tc00.1047053506377.10 | Q4CNE7 | 20,1 | 4,2 | 1 | 6 | 295 |

| | | | | | | | | |
|---|---|---|---|---|---|---|---|---|
| | | | | | | 1 | 4 | 198,5 |
| 202 | Trans-sialidase, putative (Fragment) | Tc00.1047053507995.20 | Q4CNP3 | 31 | 4,5 | 1 | 1 | 152,4 |
| | | | | | | 1 | 3 | 258,9 |
| 203 | Trans-sialidase, putative (Fragment) | Tc00.1047053421173.4 | Q4CP99 | 89,5 | 5,8 | 1 | 23 | 1906,6 |
| | | | | | | 1 | 24 | 1457,9 |
| 204 | Trans-sialidase, putative (Fragment) | Tc00.1047053510843.19 | Q4CQ58 | 28,4 | 9,5 | 1 | 4 | 565,6 |
| | | | | | | 1 | 3 | 175,4 |
| 205 | Trans-sialidase, putative (Fragment) | Tc00.1047053510981.20 | Q4CQ20 | 81,4 | 4,6 | 2 | 25 | 2257,1 |
| | | | | | | 1 | 30 | 2317 |
| 206 | Trans-sialidase, putative (Fragment) | Tc00.1047053416041.14 | Q4CTI7 | 64,2 | 7,1 | 1 | 4 | 197,3 |
| | | | | | | 1 | 15 | 958,2 |
| 207 | Trans-sialidase, putative (Fragment) | Tc00.1047053506231.10 | Q4CUC3 | 35,7 | 5 | 1 | 7 | 740,1 |
| | | | | | | 1 | 8 | 642 |
| 208 | Trans-sialidase, putative (Fragment) | Tc00.1047053510233.39 | Q4CUU0 | 75,5 | 8 | 2 | 17 | 1368,8 |
| | | | | | | 2 | 24 | 1726,6 |
| 209 | Trans-sialidase, putative (Fragment) | Tc00.1047053507121.11 | Q4D111 | 78,8 | 8,7 | 8 | 12 | 514 |
| | | | | | | 2 | 20 | 1363,1 |
| 210 | Trans-sialidase, putative (Fragment) | Tc00.1047053506895.80 | Q4D266 | 36,4 | 9 | 4 | 20 | 1881,8 |
| | | | | | | 4 | 23 | 2010,7 |
| 211 | Trans-sialidase, putative (Fragment) | Tc00.1047053510411.40 | Q4D2I6 | 62,3 | 4,9 | 1 | 23 | 1764,8 |
| | | | | | | 2 | 18 | 1089 |
| 212 | Trans-sialidase, putative (Fragment) | Tc00.1047053508297.7 | Q4D3D0 | 49,5 | 5,5 | 1 | 16 | 1344,8 |
| | | | | | | 1 | 18 | 1347 |
| 213 | Trans-sialidase, putative (Fragment) | Tc00.1047053511855.10 | Q4D6K1 | 46,7 | 8,9 | 3 | 11 | 649,5 |
| | | | | | | 1 | 8 | 240,1 |
| 214 | Trans-sialidase, putative (Fragment) | Tc00.1047053508903.110 | Q4DAA5 | 111,5 | 4,8 | 1 | 45 | 4843,2 |
| | | | | | | 2 | 52 | 4049,6 |
| 215 | Trans-sialidase, putative (Fragment) | Tc00.1047053511757.100 | Q4DBD2 | 62,3 | 8,9 | 2 | 17 | 1424,5 |
| | | | | | | 2 | 21 | 1208,3 |
| 216 | Trans-sialidase, putative (Fragment) | Tc00.1047053509765.129 | Q4DBF0 | 81,2 | 6 | 1 | 14 | 936,4 |
| | | | | | | 1 | 26 | 1830,4 |
| 217 | Trans-sialidase, putative (Fragment) | Tc00.1047053507505.10 | Q4CR17 | 74,8 | 4,8 | 1 | 41 | 3688,3 |
| | | | | | | 1 | 52 | 4334,6 |
| 218 | Trans-sialidase, putative (Fragment) | Tc00.1047053511101.11 | Q4CVW4 | 18,9 | 7,7 | 3 | 2 | 189,4 |
| | | | | | | 3 | 3 | 242,6 |
| 219 | Trans-sialidase, putative (Fragment) | Tc00.1047053506253.39 | Q4CSN1 | 60,7 | 5,9 | 1 | 23 | 1675,3 |
| | | | | | | 1 | 26 | 1263,3 |
| 220 | Trans-sialidase, putative (Fragment) | Tc00.1047053511831.10 | Q4CPA0 | 110,1 | 4,9 | 1 | 21 | 1809,3 |
| | | | | | | 1 | 16 | 860,1 |
| 221 | Trans-sialidase, putative (Fragment) | Tc00.1047053509417.9 | Q4CW35 | 70,8 | 6,6 | 2 | 15 | 1188,6 |
| | | | | | | 1 | 20 | 1778,3 |
| 222 | Trans-sialidase, putative (Fragment) | Tc00.1047053511105.60 | Q4CX91 | 85,7 | 4,6 | 1 | 25 | 2143,5 |
| | | | | | | 2 | 30 | 2468,3 |
| 223 | Trans-sialidase, putative (Fragment) | Tc00.1047053506413.89 | Q4CXV0 | 76,7 | 6,1 | 5 | 7 | 611,1 |
| | | | | | | 4 | 1 | 166,3 |

| | | | | | | | | |
|---|---|---|---|---|---|---|---|---|
| 224 | Trans-sialidase, putative (Fragment) | Tc00.1047053508165.430 | Q4E1H2 | 41,2 | 6 | 11 | 14 | 1208,2 |
| | | | | | | 7 | 11 | 538,4 |
| 225 | Trans-sialidase, putative (Fragment) | Tc00.1047053508061.154 | Q4DKB4 | 66,6 | 8,6 | 3 | 23 | 1631,6 |
| | | | | | | 1 | 22 | 1220,5 |
| 226 | Trans-sialidase, putative (Fragment) | Tc00.1047053507035.11 | Q4DNL8 | 60,5 | 7,2 | 2 | 13 | 759,4 |
| | | | | | | 2 | 20 | 1665,7 |
| 225 | Surface protease GP63, putative | Tc00.1047053510303.140 | Q4DZ07 | 81,1 | 6,3 | 1 | 24 | 2923,8 |
| | | | | | | 3 | 22 | 1628,6 |
| 226 | Surface protease GP63, putative | Tc00.1047053510303.20 | Q4DZ17 | 68,3 | 6,3 | 2 | 24 | 3022,2 |
| | | | | | | 2 | 26 | 1420,8 |
| 227 | Surface protease GP63, putative | Tc00.1047053510233.10 | Q4CUU3 | 11,8 | 4,2 | 1 | 3 | 110,1 |
| | | | | | | 1 | 4 | 130,3 |
| 228 | Surface protease GP63, putative | Tc00.1047053505567.20 | Q4CNY8 | 82,7 | 5,2 | 1 | 30 | 2692,5 |
| | | | | | | 1 | 42 | 3531,6 |
| 229 | Surface protease GP63, putative | Tc00.1047053510139.20 | Q4D273 | 90,8 | 5,7 | 1 | 13 | 1423,5 |
| | | | | | | 1 | 26 | 1851,2 |
| 230 | Surface protease GP63, putative | Tc00.1047053511117.50 | Q4D292 | 99,4 | 5,9 | 2 | 29 | 2659,3 |
| | | | | | | 1 | 39 | 3670,9 |
| 231 | Surface protease GP63, putative | Tc00.1047053508071.50 | Q4D5G1 | 82,7 | 5,2 | 11 | 38 | 3221,5 |
| | | | | | | 11 | 53 | 5209,9 |
| 232 | Surface protease GP63, putative | Tc00.1047053510477.10 | Q4D958 | 38,6 | 4,6 | 1 | 2 | 183,2 |
| | | | | | | 1 | 2 | 141,1 |
| 233 | Surface protease GP63, putative | Tc00.1047053503479.60 | Q4D6Z1 | 59,9 | 6,2 | 1 | 22 | 1783,9 |
| | | | | | | 1 | 27 | 1421,5 |
| 234 | Surface protease GP63, putative | Tc00.1047053507559.110 | Q4DAG2 | 70,5 | 6,1 | 1 | 25 | 3090,7 |
| | | | | | | 1 | 29 | 1613,7 |
| 235 | Surface protease GP63, putative | Tc00.1047053511257.100 | Q4DGK2 | 85,3 | 5,7 | 1 | 10 | 841,9 |
| | | | | | | 1 | 8 | 959,6 |
| 236 | Surface protease GP63, putative | Tc00.1047053511257.70 | Q4DGK5 | 85,3 | 5,3 | 10 | 12 | 978,6 |
| | | | | | | 9 | 11 | 1051,2 |
| 237 | Surface protease GP63, putative | Tc00.1047053510503.100 | Q4DN56 | 72,6 | 5,4 | 14 | 4 | 503,8 |
| | | | | | | 5 | 6 | 511,9 |
| 238 | Surface protease GP63, putative | Tc00.1047053509259.40 | Q4DM64 | 82,8 | 5 | 1 | 35 | 2791,8 |
| | | | | | | 1 | 40 | 3337,5 |
| 239 | Surface protease GP63, putative | Tc00.1047053506779.180 | Q4DMD2 | 80,9 | 6,5 | 1 | 20 | 2096,1 |
| | | | | | | 1 | 22 | 1497,9 |
| 240 | Surface protease GP63, putative | Tc00.1047053509369.50 | Q4DCQ9 | 48,4 | 8,9 | 1 | 8 | 843,7 |
| | | | | | | 1 | 13 | 1176 |
| 241 | Surface protease GP63, putative | Tc00.1047053506289.170 | Q4DVY6 | 76,7 | 5,3 | 1 | 11 | 596,8 |
| | | | | | | 2 | 15 | 1071 |
| 242 | Surface protease GP63, putative | Tc00.1047053506289.140 | Q4DVY9 | 88,4 | 5,2 | 3 | 12 | 873,7 |
| | | | | | | 1 | 15 | 1105,5 |
| 243 | Surface protease GP63, putative | Tc00.1047053511153.40 | Q4DHC2 | 82,4 | 6,5 | 9 | 27 | 3320,9 |
| | | | | | | 10 | 32 | 1755,6 |
| 244 | Surface protease GP63, putative | Tc00.1047053508365.100 | Q4DWF5 | 83,5 | 5,7 | 1 | 5 | 494,5 |

| | | | | | | | | |
|---|---|---|---|---|---|---|---|---|
| | | | | | | 1 | 5 | 490,5 |
| 245 | Surface protease GP63, putative | Tc00.1047053510307.10 | Q4E0N3 | 90,4 | 5,6 | 3 | 16 | 1303,4 |
| | | | | | | 3 | 28 | 2005,3 |
| 246 | Surface protease GP63, putative | Tc00.1047053506855.220 | Q4E1S2 | 60,2 | 6,1 | 1 | 21 | 1904,1 |
| | | | | | | 1 | 22 | 1995,8 |
| 247 | Surface protease GP63, putative (Fragment) | Tc00.1047053509373.39 | Q4CP23 | 24,4 | 8,9 | 1 | 9 | 1029,4 |
| | | | | | | 1 | 13 | 922,4 |
| 248 | Surface protease GP63, putative (Fragment) | Tc00.1047053508057.11 | Q4CNJ0 | 20,9 | 8,6 | 1 | 5 | 725,8 |
| | | | | | | 1 | 15 | 1243,4 |
| 249 | Surface protease GP63, putative (Fragment) | Tc00.1047053504755.10 | Q4CM26 | 40 | 4,6 | 3 | 11 | 1012,9 |
| | | | | | | 3 | 17 | 1240,5 |
| 250 | Surface protease GP63, putative (Fragment) | Tc00.1047053511703.19 | Q4CMY9 | 32,9 | 5,9 | 3 | 15 | 1784,7 |
| | | | | | | 3 | 16 | 1048,6 |
| 251 | Surface protease GP63, putative (Fragment) | Tc00.1047053509325.4 | Q4CNB7 | 21,3 | 7,5 | 1 | 9 | 1116,7 |
| | | | | | | 1 | 11 | 999,3 |
| 252 | Surface protease GP63, putative (Fragment) | Tc00.1047053506057.101 | Q4D148 | 22,1 | 8,4 | 1 | 12 | 965,7 |
| | | | | | | 1 | 16 | 1402,4 |
| 253 | Dispersed gene family protein 1 (DGF-1), putative | Tc00.1047053506415.10 | Q4CZ77 | 354,4 | 7,2 | 1 | 24 | 802,5 |
| | | | | | | 194 | 15 | 957,9 |
| 254 | Dispersed gene family protein 1 , putative | Tc00.1047053507083.20 | Q4DPN1 | 354,1 | 7,3 | 150 | 28 | 942 |
| | | | | | | 17 | 17 | 878,7 |
| 255 | Dispersed gene family protein 1 , putative | Tc00.1047053511767.20 | Q4DKA8 | 359,7 | 5,6 | 1 | 2 | 195,3 |
| | | | | | | 1 | 3 | 192,7 |
| 256 | Mucin TcMUCI, putative | Tc00.1047053504167.20 | Q4CSC0 | 17,7 | 9,1 | 1 | 2 | 86,1 |
| | | | | | | 1 | 2 | 64,1 |
| 257 | Mucin-like glycoprotein, putative | Tc00.1047053507359.20 | Q4CRJ0 | 36,5 | 4,6 | 1 | 2 | 337,6 |
| | | | | | | 1 | 2 | 237,8 |
| 258 | Mucin-like glycoprotein, putative | Tc00.1047053509123.10 | Q4CU27 | 36,5 | 4,5 | 1 | 3 | 239,2 |
| | | | | | | 1 | 5 | 188,9 |
| 259 | Mucin-like glycoprotein, putative | Tc00.1047053508737.10 | Q4DTI3 | 36,7 | 4,9 | 1 | 3 | 220,5 |
| | | | | | | 1 | 2 | 222,8 |
| 260 | Mucin-like glycoprotein, putative | Tc00.1047053508741.440 | Q4E332 | 37,9 | 4,9 | 2 | 8 | 780 |
| | | | | | | 2 | 8 | 511 |
| 261 | Mucin-associated surface protein (MASP) | Tc00.1047053510477.100 | Q4D951 | 27,4 | 4,7 | 3 | 2 | 67,3 |
| | | | | | | 3 | 2 | 67,3 |
| 262 | Mucin-associated surface protein (MASP) | Tc00.1047053511401.100 | Q4DDR2 | 45 | 4,5 | 5 | 1 | 55,5 |
| | | | | | | 5 | 1 | 152,3 |
| 263 | Mucin-associated surface protein , putative | Tc00.1047053509905.180 | Q4DR50 | 48,7 | 4,6 | 5 | 3 | 200,5 |
| | | | | | | 5 | 2 | 51,2 |
| 264 | Mucin-associated surface protein , putative | Tc00.1047053510363.320 | Q4DXN7 | 35,2 | 4,3 | 2 | 2 | 56,5 |
| | | | | | | 2 | 2 | 46,5 |
| 265 | Mucin-associated surface protein (MASP) | Tc00.1047053506763.260 | Q4DZB7 | 48,6 | 5,2 | 6 | 3 | 459,4 |
| | | | | | | 7 | 3 | 72,2 |
| 266 | Mucin-associated surface protein (MASP) | Tc00.1047053508873.10 | Q4E519 | 37,8 | 4,4 | 2 | 2 | 46,8 |
| | | | | | | 2 | 2 | 46,8 |

| | | | | | | | | |
|---|---|---|---|---|---|---|---|---|
| 267 | Mucin-associated surface protein , putative | Tc00.1047053506599.100 | Q4E1Z5 | 25,5 | 4,4 | 13 | 2 | 196,1 |
| | | | | | | 12 | 2 | 39 |
| 268 | Calreticulin, putative | Tc00.1047053509011.40 | Q4DDX3 | 46,2 | 4,8 | 2 | 2 | 38 |
| | | | | | | 2 | 1 | 58,1 |
| 269 | Neutral sphingomyelinase activation associated factor-like protein | Tc00.1047053509767.10 | Q4DSD4 | 90,5 | 5,9 | 1 | 9 | 750,8 |
| | | | | | | 2 | 7 | 534,4 |
| 270 | Peptidyl-prolyl cis-trans isomerase | Tc00.1047053508577.140 | Q4DPB9 | 21,1 | 9,1 | 2 | 6 | 569,8 |
| | | | | | | 2 | 4 | 113,4 |
| 271 | Peptidyl-prolyl cis-trans isomerase | Tc00.1047053506925.300 | Q4E4L9 | 18,8 | 8,4 | 1 | 7 | 975,6 |
| | | | | | | 1 | 8 | 486,6 |
| 272 | Complement regulatory protein, putative | Tc00.1047053509217.160 | Q4DQ07 | 113,7 | 4,9 | 2 | 10 | 573,8 |
| | | | | | | 1 | 13 | 723,2 |
| 273 | Uncharacterized protein | Tc00.1047053507707.20 | Q4CNH1 | 66 | 6,5 | 1 | 1 | 71,1 |
| | | | | | | 1 | 1 | 73,9 |
| 274 | Uncharacterized protein | Tc00.1047053510433.10 | Q4CPM9 | 112,1 | 10,1 | 2 | 2 | 169,6 |
| | | | | | | 2 | 2 | 166,5 |
| 275 | Uncharacterized protein | Tc00.1047053511521.27 | Q4CPX4 | 23,1 | 5,8 | 1 | 2 | 187,2 |
| | | | | | | 1 | 2 | 78,5 |
| 276 | Uncharacterized protein (Fragment) | Tc00.1047053506701.29 | Q4CQX1 | 41,3 | 5,5 | 1 | 3 | 209 |
| | | | | | | 1 | 2 | 56,3 |
| 277 | Uncharacterized protein (Fragment) | Tc00.1047053510717.10 | Q4CTF0 | 21,3 | 7,5 | 4 | 2 | 229,2 |
| | | | | | | 4 | 2 | 82,4 |
| 278 | Uncharacterized protein | Tc00.1047053511675.3 | Q4CUB2 | 36,5 | 4,6 | 1 | 2 | 337,6 |
| | | | | | | 1 | 6 | 246,7 |
| 279 | Uncharacterized protein | Tc00.1047053511735.70 | Q4CUQ4 | 23 | 6,2 | 1 | 3 | 319,9 |
| | | | | | | 1 | 2 | 91,9 |
| 280 | Uncharacterized protein | Tc00.1047053504199.20 | Q4CVJ1 | 16,4 | 9,3 | 2 | 3 | 94,3 |
| | | | | | | 2 | 3 | 99,4 |
| 281 | Uncharacterized protein | Tc00.1047053504001.20 | Q4CW22 | 13 | 7,8 | 1 | 3 | 110 |
| | | | | | | 1 | 3 | 105,2 |
| 282 | Uncharacterized protein | Tc00.1047053504001.10 | Q4CW23 | 13,5 | 9,1 | 2 | 2 | 84,7 |
| | | | | | | 2 | 3 | 180,8 |
| 283 | Uncharacterized protein | Tc00.1047053504087.20 | Q4D1D9 | 323 | 5,8 | 2 | 8 | 218,4 |
| | | | | | | 2 | 2 | 86,4 |
| 284 | Uncharacterized protein | Tc00.1047053507511.50 | Q4D3H5 | 16,7 | 5,9 | 1 | 4 | 443,6 |
| | | | | | | 1 | 3 | 331,8 |
| 285 | Uncharacterized protein | Tc00.1047053509047.40 | Q4D6D8 | 21,1 | 6,9 | 2 | 2 | 285,7 |
| | | | | | | 2 | 2 | 185 |
| 286 | Uncharacterized protein (Fragment) | Tc00.1047053511491.170 | Q4DHN4 | 38,1 | 5,6 | 2 | 3 | 223 |
| | | | | | | 2 | 2 | 52,6 |
| 287 | Uncharacterized protein | Tc00.1047053506871.190 | Q4DNJ6 | 16,8 | 6,3 | 1 | 4 | 428,8 |
| | | | | | | 1 | 3 | 324,3 |
| 288 | Uncharacterized protein | Tc00.1047053510877.40 | Q4DPV6 | 24,4 | 9,7 | 2 | 2 | 64,3 |
| | | | | | | 2 | 2 | 92,1 |
| 289 | Uncharacterized protein | Tc00.1047053511621.120 | Q4DT54 | 23,6 | 5,9 | 2 | 2 | 184,4 |

| | | | | | | | | |
|---|---|---|---|---|---|---|---|---|
| | | | | | | 4 | 2 | 75,8 |
| 290 | Uncharacterized protein | Tc00.1047053506605.120 | Q4DUX0 | 22,3 | 4,7 | 1 | 1 | 64,1 |
| | | | | | | 1 | 1 | 64,1 |
| 291 | Heat shock protein 85, putative | Tc00.1047053507713.30 | Q4CQS6 | 80,7 | 5,1 | 4 | 10 | 1062,8 |
| | | | | | | 4 | 14 | 1029,1 |
| 292 | Heat shock 70 kDa protein, putative (Fragment) | Tc00.1047053510439.61 | Q4CU95 | 40,8 | 6,2 | 2 | 20 | 1261,6 |
| | | | | | | 2 | 18 | 1064,5 |
| 293 | Heat shock 70 kDa protein, mitochondrial | Tc00.1047053507029.30 | Q4CVR9 | 70,9 | 5,8 | 4 | 3 | 221,8 |
| | | | | | | 1 | 2 | 127,5 |
| 294 | Glucose-regulated protein 78, putative | Tc00.1047053506585.40 | Q4D620 | 71,3 | 5,1 | 1 | 3 | 688,6 |
| | | | | | | 1 | 8 | 608,3 |
| 295 | Heat shock 70 kDa protein, putative (Fragment) | Tc00.1047053506135.9 | Q4DAZ6 | 30,1 | 6,2 | 1 | 7 | 778,1 |
| | | | | | | 1 | 10 | 896,1 |
| 296 | 10 kDa heat shock protein, putative | Tc00.1047053508209.120 | Q4DFA8 | 10,7 | 9 | 3 | 4 | 283,5 |
| | | | | | | 3 | 2 | 190,4 |
| 297 | Heat shock protein 70 (HSP70), putative | Tc00.1047053511211.160 | Q4DTM9 | 70,9 | 5,8 | 4 | 19 | 1716,5 |
| | | | | | | 2 | 22 | 1858,6 |
| 298 | Chaperonin HSP60, mitochondrial | Tc00.1047053507641.290 | Q4DYP5 | 59,1 | 5,4 | 4 | 6 | 217,7 |
| | | | | | | 4 | 5 | 213,2 |
| 299 | IgE-dependent histamine-releasing factor | Tc00.1047053506207.50 | Q4CW52 | 19,6 | 4,5 | 2 | 3 | 281,1 |
| | | | | | | 2 | 3 | 114,6 |
| 300 | Phosphoglycerate kinase | Tc00.1047053511419.40 | Q4D193 | 44,4 | 6,2 | 4 | 2 | 43,5 |
| | | | | | | 4 | 2 | 48,7 |
| 301 | Calcium-binding protein, putative | Tc00.1047053509391.10 | Q4D1Q2 | 23,7 | 4,9 | 9 | 8 | 415,8 |
| | | | | | | 9 | 8 | 427 |
| 302 | Calmodulin, putative (Fragment) | Tc00.1047053506389.79 | Q4D2S5 | 9,5 | 3,9 | 4 | 3 | 192,4 |
| | | | | | | 4 | 3 | 191,7 |
| 303 | Ras-related protein rab-5, putative (Fragment) | Tc00.1047053511269.4 | Q4D504 | 20,4 | 5,3 | 2 | 1 | 152,1 |
| | | | | | | 2 | 1 | 152,1 |
| 304 | Serine/threonine-protein phosphatase | Tc00.1047053510687.40 | Q4D9Y4 | 35 | 5,2 | 1 | 1 | 138,7 |
| | | | | | | 1 | 1 | 67,1 |
| 305 | 14-3-3 protein, putative | Tc00.1047053508851.180 | Q4DJB6 | 29,9 | 5 | 1 | 4 | 219,1 |
| | | | | | | 1 | 4 | 173,5 |
| 306 | Ras-related protein rab-2a, putative (Fragment) | Tc00.1047053506425.169 | Q4DM40 | 10,5 | 9,3 | 2 | 2 | 203 |
| | | | | | | 2 | 2 | 69,3 |
| 307 | Serine/threonine-protein phosphatase | Tc00.1047053508815.110 | Q4DMJ3 | 34,4 | 5,5 | 1 | 3 | 150,5 |
| | | | | | | 1 | 2 | 115,5 |
| 308 | 14-3-3 protein, putative | Tc00.1047053506775.80 | Q4DRH6 | 29,1 | 5 | 2 | 2 | 171,7 |
| | | | | | | 2 | 4 | 120,3 |
| 309 | Nucleoside diphosphate kinase | Tc00.1047053508707.200 | Q4E256 | 16,9 | 8,5 | 1 | 3 | 294,2 |
| | | | | | | 1 | 3 | 220,8 |
| 310 | Elongation factor 1-alpha (Fragment) | Tc00.1047053510119.9 | Q4CXI2 | 42,8 | 7,6 | 8 | 3 | 223,2 |
| | | | | | | 8 | 6 | 169,6 |
| 311 | Elongation factor 2, putative | Tc00.1047053510963.90 | Q4D3T1 | 94,1 | 5,7 | 5 | 6 | 496,6 |
| | | | | | | 5 | 5 | 286,1 |

| | | | | | | | | |
|---|---|---|---|---|---|---|---|---|
| 312 | 40S ribosomal protein S18, putative | Tc00.1047053506679.100 | Q4E093 | 17,5 | 10,8 | 1 | 1 | 152,8 |
| | | | | | | 1 | 2 | 49,2 |
| 313 | 40S ribosomal protein S15a, putative | Tc00.1047053506297.330 | Q4E0N6 | 14,7 | 9,8 | 3 | 1 | 152,8 |
| | | | | | | 3 | 1 | 53,3 |
| 314 | Calpain-like cysteine peptidase, putative | Tc00.1047053508675.29 | Q4D066 | 12,8 | 5,1 | 2 | 2 | 43,5 |
| | | | | | | 2 | 2 | 120 |
| 315 | Cruzipain, putative | Tc00.1047053507603.270 | Q4DW02 | 49,9 | 5,7 | 3 | 3 | 117 |
| | | | | | | 3 | 2 | 95,4 |
| 316 | Cysteine peptidase, putative | Tc00.1047053509429.320 | Q4E0J7 | 49,8 | 5,4 | 6 | 3 | 242,3 |
| | | | | | | 5 | 4 | 161,3 |
| 317 | Cysteine peptidase C , putative | Tc00.1047053511827.100 | Q4DQB0 | 36,7 | 5,8 | 2 | 2 | 97,2 |
| | | | | | | 2 | 2 | 46,9 |
| 318 | Proteasome regulatory ATPase subunit 1 | Tc00.1047053506857.90 | Q4D9J1 | 48,5 | 7,1 | 1 | 1 | 60,7 |
| | | | | | | 1 | 1 | 45,5 |
| 319 | Proteasome regulatory ATPase subunit 2 | Tc00.1047053511047.50 | Q4D0B9 | 49 | 5,5 | 1 | 3 | 85,4 |
| | | | | | | 1 | 1 | 54,8 |
| 320 | Serine carboxypeptidase (CBP1), putative | Tc00.1047053508671.20 | Q4CMQ4 | 59,5 | 6,8 | 2 | 13 | 844,7 |
| | | | | | | 2 | 12 | 694 |
| 321 | Serine carboxypeptidase (CBP1), putative | Tc00.1047053509695.210 | Q4DTP7 | 51,5 | 5 | 2 | 11 | 566,7 |
| | | | | | | 1 | 13 | 754,4 |
| 322 | Serine carboxypeptidase S28, putative | Tc00.1047053506425.10 | Q4DM56 | 72,1 | 6,2 | 3 | 5 | 386,1 |
| | | | | | | 3 | 5 | 145,8 |
| 323 | Enolase, putative | Tc00.1047053504105.140 | Q4DZ98 | 46,4 | 5,9 | 1 | 9 | 786,2 |
| | | | | | | 1 | 11 | 382,3 |
| 324 | Glyceraldehyde 3-phosphate dehydrogenase | Tc00.1047053511461.14 | Q4D3Y9 | 14,7 | 11,4 | 6 | 2 | 182,6 |
| | | | | | | 6 | 1 | 108,2 |
| 325 | Glyceraldehyde 3-phosphate dehydrogenase | Tc00.1047053509065.60 | Q4DHF0 | 39 | 9 | 2 | 2 | 133,9 |
| | | | | | | 2 | 2 | 74,2 |
| 326 | Isocitrate dehydrogenase [NADP] | Tc00.1047053506925.319 | Q4E4L7 | 46,8 | 7,7 | 1 | 1 | 164,4 |
| | | | | | | 1 | 2 | 181 |
| 327 | Lysosomal alpha-mannosidase, putative | Tc00.1047053506195.120 | Q4DXL4 | 111,2 | 6,5 | 2 | 5 | 108,8 |
| | | | | | | 2 | 3 | 224 |
| 328 | Malate dehydrogenase (Fragment) | Tc00.1047053507883.109 | Q4D4A0 | 31,5 | 7,6 | 2 | 3 | 218,3 |
| | | | | | | 2 | 1 | 152,1 |
| 329 | Fructose-bisphosphate aldolase | Tc00.1047053510301.20 | Q4D0Q0 | 40,8 | 8,8 | 4 | 5 | 153,2 |
| | | | | | | 4 | 2 | 73,7 |
| 330 | Cytochrome c, putative | Tc00.1047053506949.50 | Q4D480 | 12,2 | 9,4 | 1 | 3 | 135,4 |
| | | | | | | 2 | 3 | 95,1 |
| 331 | Cytochrome c, putative | Tc00.1047053508959.4 | Q4CV48 | 12,2 | 9,6 | 1 | 2 | 126,7 |
| | | | | | | 2 | 3 | 79,2 |
| 332 | Glutamate dehydrogenase | Tc00.1047053508111.30 | Q4D5C2 | 45 | 8 | 2 | 6 | 424,7 |
| | | | | | | 2 | 2 | 81,2 |
| 333 | Glutathione peroxidase | Tc00.1047053503899.119 | Q4DEJ5 | 19,7 | 9 | 5 | 6 | 575 |
| | | | | | | 5 | 3 | 73,6 |
| 334 | NAD/FAD dependent dehydrogenase | Tc00.1047053505843.40 | Q4CVH0 | 43 | 5,3 | 1 | 1 | 152,1 |

| | | | | | | | | |
|---|---|---|---|---|---|---|---|---|
| | | | | | | 1 | 1 | 152,1 |
| 335 | Tryparedoxin peroxidase, putative | Tc00.1047053507259.10 | Q4CM56 | 22,4 | 6,3 | 4 | 8 | 881,8 |
| | | | | | | 4 | 6 | 279,6 |
| 336 | Tryparedoxin peroxidase, putative | Tc00.1047053509499.14 | Q4CX87 | 25,5 | 7,6 | 1 | 2 | 305,4 |
| | | | | | | 1 | 2 | 195,6 |
| 337 | Tryparedoxin, putative | Tc00.1047053509997.30 | Q4D1B8 | 16 | 5,2 | 1 | 6 | 486 |
| | | | | | | 1 | 5 | 155,9 |
| 338 | Superoxide dismutase | Tc00.1047053511715.10 | Q4D5A6 | 23,3 | 7,1 | 4 | 3 | 301,3 |
| | | | | | | 4 | 3 | 301,3 |
| 339 | ADP-ribosylation factor 1, putative | Tc00.1047053508415.40 | Q4D7Y8 | 20,7 | 8,5 | 2 | 7 | 922,4 |
| | | | | | | 2 | 4 | 193,1 |
| 340 | Dynein light chain, putative | Tc00.1047053506925.104 | Q4E4N7 | 10,4 | 6,3 | 1 | 1 | 152,1 |
| | | | | | | 1 | 1 | 151 |
| 341 | Rab7 GTP binding protein, putative | Tc00.1047053508461.270 | Q4E4T4 | 23,9 | 5,8 | 1 | 2 | 303,8 |
| | | | | | | 1 | 2 | 165,9 |
| 342 | Small GTP-binding protein Rab1, putative | Tc00.1047053503715.30 | Q4CZR0 | 22,8 | 7,6 | 2 | 2 | 314,1 |
| | | | | | | 5 | 2 | 173,7 |
| 343 | Transitional endoplasmic reticulum ATPase | Tc00.1047053509733.170 | Q4DWB5 | 86,1 | 5,4 | 1 | 11 | 1336,8 |
| | | | | | | 1 | 8 | 725,8 |
| 344 | Adenosylhomocysteinase | Tc00.1047053511229.50 | Q4D455 | 48,4 | 6,2 | 2 | 2 | 74,4 |
| | | | | | | 2 | 3 | 81,9 |
| 345 | Arginine kinase, putative | Tc00.1047053507241.30 | Q4CWA5 | 40,2 | 6,3 | 2 | 9 | 852,6 |
| | | | | | | 2 | 11 | 415,2 |
| 346 | ATP synthase subunit beta | Tc00.1047053509233.180 | Q4DTX7 | 55,7 | 5,3 | 1 | 2 | 157,4 |
| | | | | | | 1 | 2 | 69,1 |
| 347 | Seryl-tRNA synthetase, putative (Fragment) | Tc00.1047053511163.10 | Q4CW46 | 25,7 | 5,5 | 2 | 1 | 153,1 |
| | | | | | | 2 | 2 | 102,2 |
| 348 | Ubiquitin-conjugating enzyme E2, putative | Tc00.1047053508137.30 | Q4CTN0 | 17,5 | 7,9 | 2 | 2 | 138,9 |
| | | | | | | 2 | 2 | 41,1 |
| 349 | Ubiquitin-activating enzyme E1, putative | Tc00.1047053504427.250 | Q4DYM1 | 114,3 | 5,6 | 2 | 1 | 152,4 |
| | | | | | | 2 | 1 | 57,8 |
| 350 | Alpha tubulin, putative | Tc00.1047053411235.9 | Q4CLA1 | 49,8 | 4,9 | 1 | 23 | 2188,8 |
| | | | | | | 1 | 31 | 1609,4 |
| 351 | Beta tubulin, putative | Tc00.1047053506563.40 | Q4DQP2 | 49,7 | 4,7 | 1 | 21 | 1962,2 |
| | | | | | | 1 | 19 | 1204,4 |
| 352 | Actin, putative | Tc00.1047053510127.79 | Q4D7A6 | 38,1 | 5,4 | 3 | 2 | 170,4 |
| | | | | | | 3 | 2 | 180,8 |
| 353 | Cofilin/actin depolymerizing factor, putative | Tc00.1047053508411.10 | Q4D8D3 | 15,7 | 5,7 | 1 | 3 | 112,4 |
| | | | | | | 1 | 3 | 84,7 |
| 354 | Cofilin/actin depolymerizing factor, putative | Tc00.1047053510145.20 | Q4CVE9 | 15,7 | 5,7 | 1 | 4 | 134,8 |
| | | | | | | 1 | 3 | 84 |
| 355 | Microtubule-associated protein, putative (Fragment) | Tc00.1047053507447.19 | Q4CMT2 | 85,2 | 4,8 | 2 | 4 | 262 |
| | | | | | | 2 | 7 | 372,5 |
| 356 | Surface protein TolT | Tc00.1047053508767.20 | Q4CM38 | 33 | 8,5 | 1 | 3 | 214 |
| | | | | | | 3 | 2 | 83,9 |

| | | | | | | | | |
|---|---|---|---|---|---|---|---|---|
| 357 | Surface protein TolT | Tc00.1047053506617.20 | Q4D0C6 | 33 | 8,7 | 3 | 4 | 259,3 |
| | | | | | | 4 | 2 | 83,8 |
| 358 | Surface protein TolT, putative | Tc00.1047053504277.11 | Q4CNL2 | 21,5 | 8,8 | 1 | 5 | 362 |
| | | | | | | 1 | 3 | 66,4 |
| 359 | Surface protein TolT (Fragment) | Tc00.1047053508767.10 | Q4CM39 | 24,8 | 9,4 | 2 | 5 | 437,1 |
| | | | | | | 3 | 3 | 107,6 |
| 360 | S-adenosylmethionine synthase | Tc00.1047053506945.160 | Q4CSC4 | 43,5 | 6 | 1 | 1 | 152,6 |
| | | | | | | 1 | 2 | 81,9 |
| 361 | Arginyl-tRNA synthetase, putative | Tc00.1047053508355.320 | Q4E397 | 78,8 | 6,1 | 1 | 1 | 147,7 |
| | | | | | | 1 | 2 | 65,2 |
| 362 | Cysteine peptidase inhibitor | Tc00.1047053506801.80 | Q4DH32 | 12 | 6,6 | 1 | 6 | 801,8 |
| | | | | | | 1 | 8 | 554,4 |
| 363 | Cysteine peptidase inhibitor, putative | Tc00.1047053511907.200 | Q4DY71 | 12,1 | 6,6 | 1 | 6 | 818,5 |
| | | | | | | 1 | 7 | 637,9 |
